# Integrating Solid-State NMR and Computational Modeling to Investigate the Structure and Dynamics of Membrane-Associated Ghrelin

**DOI:** 10.1371/journal.pone.0122444

**Published:** 2015-03-24

**Authors:** Gerrit Vortmeier, Stephanie H. DeLuca, Sylvia Els-Heindl, Constance Chollet, Holger A. Scheidt, Annette G. Beck-Sickinger, Jens Meiler, Daniel Huster

**Affiliations:** 1 Institute of Medical Physics and Biophysics, University of Leipzig, Leipzig, Germany; 2 Center for Structural Biology, Vanderbilt University, Nashville, Tennessee, 37232, United States of America; 3 Institute of Biochemistry, University of Leipzig, Leipzig, Germany; University of Pittsburgh School of Medicine, UNITED STATES

## Abstract

The peptide hormone ghrelin activates the growth hormone secretagogue receptor 1a, also known as the ghrelin receptor. This 28-residue peptide is acylated at Ser3 and is the only peptide hormone in the human body that is lipid-modified by an octanoyl group. Little is known about the structure and dynamics of membrane-associated ghrelin. We carried out solid-state NMR studies of ghrelin in lipid vesicles, followed by computational modeling of the peptide using Rosetta. Isotropic chemical shift data of isotopically labeled ghrelin provide information about the peptide’s secondary structure. Spin diffusion experiments indicate that ghrelin binds to membranes via its lipidated Ser3. Further, Phe4, as well as electrostatics involving the peptide’s positively charged residues and lipid polar headgroups, contribute to the binding energy. Other than the lipid anchor, ghrelin is highly flexible and mobile at the membrane surface. This observation is supported by our predicted model ensemble, which is in good agreement with experimentally determined chemical shifts. In the final ensemble of models, residues 8–17 form an α-helix, while residues 21–23 and 26–27 often adopt a polyproline II helical conformation. These helices appear to assist the peptide in forming an amphipathic conformation so that it can bind to the membrane.

## Introduction

Ghrelin, a 28-amino acid peptide hormone, is the endogenous ligand of the growth hormone secretagogue receptor 1a (GHSR), a G protein-coupled receptor (GPCR) [1–3]. In addition to stimulating the release of growth hormone from the pituitary [1–3], it has been implicated in appetite stimulation [4], insulin and glucagon secretion levels [5], decreased blood pressure [6], inhibition of apoptosis in cardiomyocytes and endothelial cells, and cell proliferation and differentiation [7]. Further, circulating ghrelin levels have been found to change in patients with diseases involving perturbed energy balance, such as obesity [8–12] and diabetes [13], see reference [14] for thorough review. Given the current prevalence and rapidly increasing rates of obesity and related conditions, it is important to understand the mechanism of action of ghrelin in order to eventually contribute to the understanding of the molecular basis of these diseases.

Interestingly, ghrelin carries a unique *n*-octanoyl group at position Ser3, the catalysis for which is performed post-translationnally by the ghrelin-O-acyltransferase. Ghrelin represents the only hormone in the human body that is lipid modified. Although the desacylated form of ghrelin is the most abundant in the bloodstream, the fatty acid modification proves necessary for receptor binding and activation. The initial identification of acylated ghrelin revealed an octanoyl group at Ser3 [2], but ghrelin O-acyltransferase can add fatty acid moieties of varying lengths to the peptide [15–17]. Remarkably, the length of the lipid side chain has a demonstrated effect on the ability of ghrelin to activate GHSR and on levels of adiposity in mice [18].

Bednarek, *et al*. identified a short N-terminal segment, spanning from Gly1 to Phe4, including the octanoylated Ser3, that is able to activate the GHSR *in vitro* [19] but this active core neither displaces ghrelin from its receptor nor stimulates growth hormone release *in vivo* [20]. This may be due to the influence of the membrane surface on transport and receptor binding. Membrane binding of a ligand is a crucial step for membrane-receptor activation. The limitation of ligand diffusion to two dimensions, as well as structural pre-orientation and pre-organization of the ligand, may lead to enhanced peptide-receptor interaction probability [21]. However, more structural and dynamic information of the peptide in solution, membrane-bound, and receptor-bound states is needed to further examine this so-called “membrane catalysis”.

Our current understanding of the structure of membrane-bound ghrelin is fragmentary at best. Spectroscopic studies from solution ^1^H nuclear magnetic resonance (^1^H NMR) and circular dichrosim (CD) spectroscopy of ghrelin in solution revealed a highly flexible peptide without a distinct structure, regardless of whether or not Ser3 was acylated [22]. CD experiments conducted in the membrane mimics, sodium dodecyl sulfate (SDS) and trifluoroethanol (TFE), showed formation of an α-helix with increasing TFE content [23], and molecular dynamics (MD) simulations in water and in 1,2-dihexanoyl-*sn*-glycero-3-phosphocholine/DMPC bilayer/water systems suggest that this helix extends from Pro7 to Gln13 [24]. Chemical shift (CS) data from ^1^H NMR experiments performed in phosphate buffered saline and in live cells also indicated a putative α-helix between residues Glu8 and Lys20, while the peptide remained seemingly unstructured in water [25]. A structure of desacyl-ghrelin solved with CS data from solution ^1^H NMR data performed in a water/hexafluoroacetone mixture supports the presence of a stable α-helix spanning from Pro7 to Gln14 [26]. Furthermore, controversial results were published about the membrane binding segment. While simulations propose a C-terminal loop that mediates binding, with the octanoyl moiety pointing towards the aqueous phase [24], solution NMR experiments suggested that the peptide binds to detergent micelles via Phe4 and the lipid-modified Ser3 [27].

Lipid modifications typically serve as membrane anchors [28,29]. However, a short octanoyl chain is only weakly hydrophobic, and the strength of its interaction with the membrane has yet to be determined. In order to characterize the structure and dynamics of octanoylated ghrelin and how it interacts with the membrane, we employed solid-state NMR spectroscopy (ssNMR), which has been demonstrated to be a useful and versatile tool for studying membrane-associated proteins and peptides [30–32]. We show that ghrelin binds to large unilamellar vesicles (LUVs) via its octanoyl chain and assumes a highly mobile structure at the membrane surface.

Previous research indicates that ghrelin is highly flexible, even in the presence of membranes, and its secondary structure propensities in LUVs remain unknown. Therefore, the CSs obtained from ssNMR were used in combination with the Rosetta molecular modeling software [33–35], which has been widely used for protein structure prediction. NMR CSs can be used to enhance Rosetta’s ability to sample native-like structures [36–39] with modeling of membrane and membrane-associated proteins becoming more feasible. For instance, the structure of hepatitis C virus protein p7, a small, helical membrane protein, was determined using the RosettaMembrane environment [40,41] with NMR CS, residual dipolar coupling, and paramagnetic relaxation enhancement structural data [42]. In addition to the extensive ssNMR studies mentioned above, we present a new, detailed protocol for elucidating the structural ensemble of membrane-associated ghrelin that is consistent with sparse CS data.

## Materials and Methods

### Materials

1,2-Dimyristoyl-sn-glycero–3-phosphocholine (DMPC), 1,2-dimyristoyl(d_54_)-sn-glycero–3-phosphocholine (DMPC-d_54_), 1,2-dimyristoyl(d_54_)-sn-glycero–3-phosphocholine–1,1,2,2-d_4_-N,N,N-trimethyl-d_9_ (DMPC-d_67_), 1,2-dimyristoyl-sn-glycero–3-phosphatidylserine (DMPS), and 1,2-dimyristoyl(d_54_)-sn-glycero–3-phosphatidylserine (DMPS-d_54_) were purchased from Avanti Polar Lipids, Inc. (Alabaster, AL) and used without further purification. ^13^C/^15^N Fmoc-protected amino acids and deuterated octanoic acid were obtained from Euriso-Top GmbH, Saarbrücken, Germany. All other materials were purchased from Sigma, Deisenhofen, Germany.

### Peptide Synthesis

Ghrelin analogs were synthesized automatically on a Wang resin by solid-phase peptide synthesis using Fmoc/tBu protection group strategy on a robot system (SyroI, MultiSynTech, Bochum, Germany), as described previously [43]. ^13^C/^15^N-labeled amino acids were introduced via manual peptide coupling using 5 equiv Fmoc-amino acid, 5 equiv DIC and 5 equiv HOBt in DMF. To enable the incorporation of octanoic acid or perdeuterated octanoic acid, Ser3 was introduced with the labile Trt side chain protecting group. Ester bond was formed by incubation of 5 equiv octanoic acid, 5 equiv DMAP, 5 equiv DCC in NMP with the resin. The final peptides were cleaved from the resin in one step, and purification was achieved by preparative HPLC on a reversed-phase C18 column (Phenomenex Jupiter 10u Proteo 90 Å: 250 × 21.2 mm^2^; 7.8 μm; 90 Å). Peptides were analyzed by MALDI-TOF MS (UltraflexII, Bruker, Bremen, Germany) and by analytical reversed-phase HPLC on columns VariTide RPC (Varian: 250 × 4.6 mm^2^; 6 μm; 200 Å) and Phenomenex Jupiter 4u Proteo 300 Å (Phenomenex: 250 × 4.6 mm^2^; 4 μm; 300 Å). The observed masses were in full agreement with the calculated masses, and peptide purity ≥ 95% could be obtained according to the analytical RP-HPLC.

### Sample Preparation

Aliquots of lipids were co-dissolved in chloroform; the solvent was evaporated, and the lipid film was suspended in 10 mM MES buffer (100 mM NaCl, pH 6) to reach a final concentration of 20 mM. After freeze-thaw cycles, the suspension was extruded across 100 nM polycarbonate membranes to produce LUVs [44]. Aliquots of ghrelin were added to the LUVs to reach the desired peptide/lipid ratio. Samples were incubated for 2 h while shaking it at 190 rpm at 37°C. A more homogeneous distribution of ghrelin between the outer and the inner membrane leaflet was achieved by performing another five freeze-thaw cycles. The sample was ultracentrifuged at ~90,000 × g for 8 h. After lyophilization, the precipitate was hydrated to 35 wt% water content, mixed with 5 freeze-thaw cycles, and transferred into 4 mm MAS rotors with Teflon inserts.

### Membrane Binding Assay

For membrane binding analysis of ghrelin, 5 μM peptide solutions were ultracentrifuged with various amounts of 176 mM sucrose-loaded DMPC/DMPG vesicles (5:1, mol/mol). For each vesicle concentration, 10 μL of a 50 μM peptide solution in H_2_O were added to 740 μL of iso-osmolar 1 mM MOPS buffer at pH 7, containing 100 mM KCl. Vesicle solutions of various lipid concentrations ranging from 0 to 10 mM were added to reach a final volume of 1 mL. Each concentration was prepared in duplicate, and lipid-only samples were taken to determine background signals. After vortexing and 30 min incubation at room temperature, samples were ultracentrifuged overnight at ~90,000 × g and 4°C. Immediately after centrifugation, ~900 μL supernatant were transferred into Eppendorf tubes. Pellets were resuspended in the remaining solution (~100 μL) and diluted by adding 900 μL buffer. 600 μl of both the supernatant and the pellet solutions were used to determine peptide concentration using a fluorescamine assay [45]. The remaining volumes were used to measure the lipid concentration.

The pH of the samples was elevated to 10 using 5 μL of 0.1 M KOH. 250 μL of a fluorescamine stock solution in acetone (1 mg/mL) was added to the samples, and the fluorescence was measured after ~5 min with excitation at 390 nm and emission at 475 nm. Background fluorescence determined from the lipid-only samples was subtracted, and the percentage of bound peptide was calculated according to the equation:

$$\text{\%}peptidebound=\frac{\text{1}-I_{supernatant}}{I_{supernatant}+I_{pellet}}\cdot \text{100}$$ (1)

The final lipid concentration was determined by phosphate analysis. Approximately 90% of the lipids were in the pellet fraction. Further, half of the lipids are not accessible for the peptide because the molecules are on the inside of the vesicles. Accordingly, the lipid concentrations were corrected with the factor 0.45 to deliver the effective lipid concentration [*L*]_eff_ [46]. No freeze-thaw cycles were applied in the binding assay.

### Solid-State NMR Spectroscopy

^2^H NMR spectra were acquired using an Avance 750 MHz NMR spectrometer (Bruker Biospin, Rheinstetten, Germany) operating at a resonance frequency of 115.0 MHz for ^2^H using a quadrupolar-echo pulse sequence, a 90°-pulse length of 2.8 μs, an echo time of 60 μs, and a relaxation delay of 0.75 s. Smoothed chain order parameter profiles were calculated from the quadrupolar splittings after dePaking, as described in reference [47]. Standard ^31^P NMR spectra were acquired on a Bruker DRX300 NMR spectrometer operating at a resonance frequency of 121.4 MHz using a standard Hahn echo pulse sequence with a 90° pulse length of 10.75 μs, a delay between pulses of 50 μs, and a relaxation delay of 2.5 s. The ^13^C magic angle spinning (MAS) NMR spectra were acquired using a Bruker Avance III 600 NMR spectrometer at resonance frequencies of 600.1 MHz and 150.9 MHz for ^1^H and ^13^C, respectively. Typical ^1^H and ^13^C 90° pulse lengths were 4 and 5 μs, respectively, while the decoupling filed during acquisition was ~65 kHz using Spinal64. Standard CP (contact time 700 μs), directly excited, and INEPT excitation schemes were used. All CSs were referenced to external crystalline glycine at 176.46 ppm (relative to TMS) at 30°C. Standard 2D HetCor [48] and proton-driven spin diffusion (PDSD) [49] spectra were acquired, with a total evolution time of 7.1 ms and 1.7 ms in the ^1^H and ^13^C indirect dimensions, respectively. Constant time DIPSHIFT experiments [50] were carried out at a MAS frequency of 4 kHz with FSLG homonuclear decoupling. Dipolar dephasing curves were simulated as described in the literature [51]. The ratio of the motional averaged and full dipolar coupling [52] defined the molecular order parameter, S.

Spin diffusion experiments from the lipid into ghrelin were carried out using the pulse sequence from the literature [53]. A *T*_*2*_ filter of 6 ms and spin diffusion times from 0.01 to 900 ms were used. Peak intensities were corrected for relaxation using the measured *T*_1_ relaxation times. Subsequently, the intensities were normalized to 1 for the longest spin diffusion time of 900 ms as described in [53,54]. Spin diffusion build-up curves were simulated as a function of mixing time using a one-dimensional lattice model [54]. In this model, the magnetization of a given spin (*M*_i_) is transferred to the neighboring spins (*M*_i−1_ and *M*_i+1_) according to:

$$\Delta M_{i}/\Delta t_{m}=-2\Omega M_{i}+\Omega M_{i+1}+\Omega M_{i-1}$$ (2)

The rate of magnetization transfer, Ω = *D* / *a* depends on the spin diffusion coefficient, *D*, and the distance between spins, *a*. Simulations were carried out using *D* = 0.001 nm^2^/s and *a* = 2 Å.

### Overview of Structure Determination Using Rosetta

**Fig 1:**
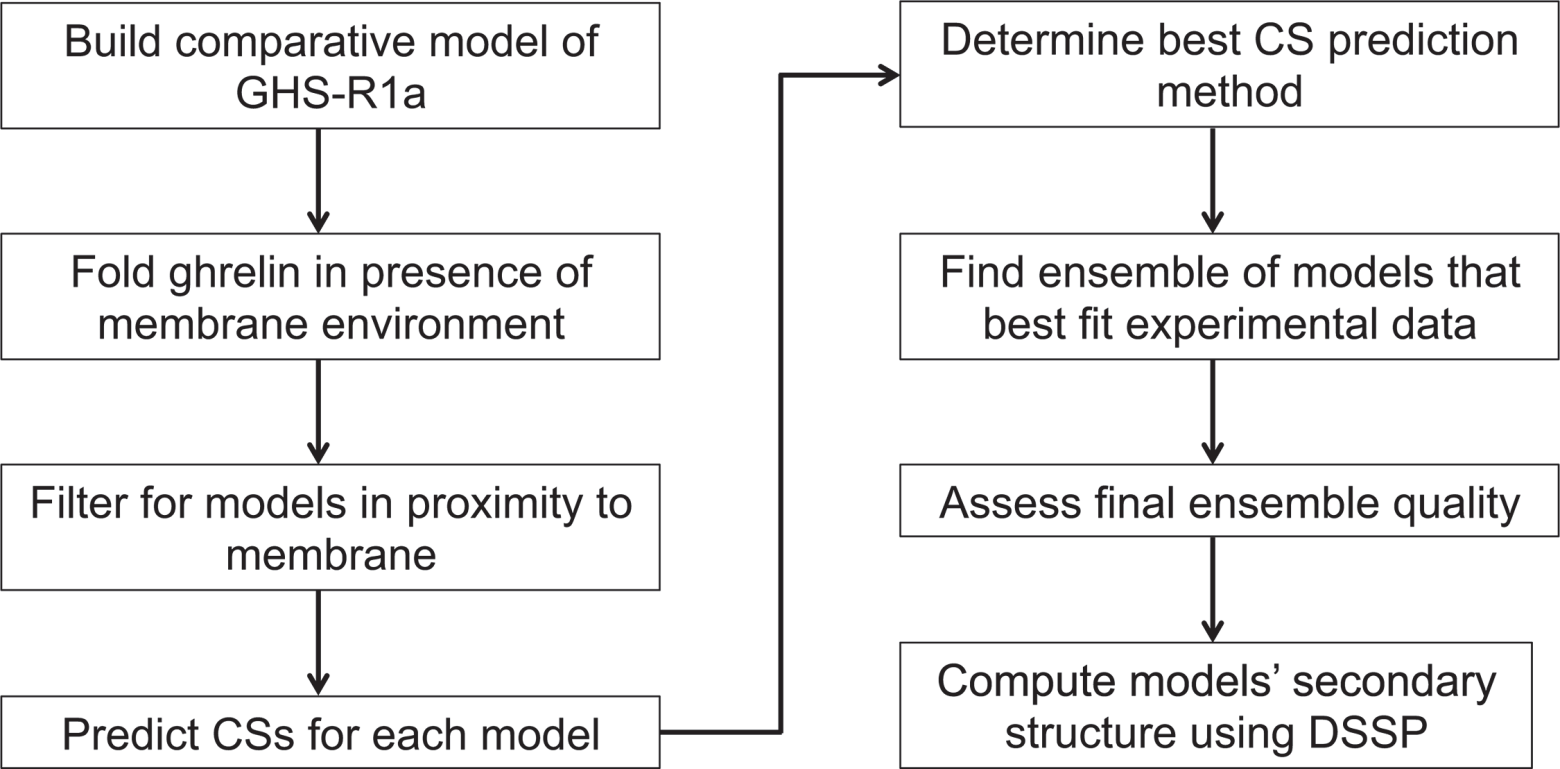
Flowchart of computational modeling and analysis protocol. The flowchart outlines the protocol used to elucidate the structure of ghrelin based on ssNMR CS data.

The Rosetta Topology Broker framework [38,55,56] was employed to fold ghrelin *de novo*, or from the sequence, in the presence of the implicit RosettaMembrane scoring environment [40,41] For three-dimensional structure prediction, the traditional Rosetta fragment-based assembly algorithm for soluble proteins was employed [33,57] The modeling and analysis protocol is summarized in Fig. 1, and full details are available in the S1 File and S2 File.

### Definition of Membrane Location in Rosetta

In order to fold membrane-associated proteins using Rosetta, transmembrane helical (TMH) regions must be specified. Therefore, because the modeling objective was to fold ghrelin at the membrane interface, a comparative model of GHSR was created based on an alignment of 19 different GHSR sequences and the sequences of twenty GPCRs of known structure (S1 Fig., S1 File). This receptor model was only used as a proxy to define the membrane location; that is, no interaction between receptor and peptide occurs. In the starting conformation for peptide folding, the receptor was placed more than 50 Å away from the peptide. During selection of the final ensemble, only models having a minimum interatomic receptor-to-peptide distance of 5 Å were analyzed and compared to experimental CSs. The full protocol for generation of the comparative model is described in S1 File and S2 File.

### Fragment Selection of Ghrelin in Rosetta

A complete set of CSs can greatly increase the quality of fragments selected for Rosetta *de novo* structure prediction [36,58]. Fragment selection for *de novo* folding in Rosetta heavily prioritizes peptide fragment conformations that have the same secondary structure as that indicated by CS analysis [36,59,60] In the case of ghrelin, however, the CS dataset is incomplete, i.e. CS assignments are not available for every residue. This leads to inconsistencies in fragment selection, where CSs of a few residues can determine the secondary structure of the entire fragment. In the present case, the CS data suggest that residues 2–5 have β-strand torsion angles. Accordingly, these residues are often constructed from fragments that stem from β-hairpins (S2 Fig.). In result, even though the fewer CSs obtained for residues 8–28 are indicative of a random coil region with a slight helical tendency, the vast majority of Rosetta models generated from fragments selected based on the sparse CS dataset exhibited a β-hairpin fold (data not shown). Therefore, we elected to fold with fragments not generated using CS data, thereby sampling the complete conformational space reasonable for a peptide of this sequence. We then employed CS data to filter from a large pool of models an ensemble that agreed best with the CS data. This approach has another advantage in the case of highly flexible peptides in that the ensemble average CSs, not the CS of a single model, must conform to the experimental data.

### *De Novo* Folding of Ghrelin in Rosetta

During folding in the Topology Broker framework, 3- and 9-amino acid peptide fragments were inserted into an extended backbone of the peptide in a Monte Carlo fashion. The resulting conformations were scored with the RosettaMembrane [40,61] potentials according to the Metropolis criterion [62]. Ten thousand models were generated in the presence of the membrane and relaxed within the all-atom membrane potential (for detail protocol capture see S1 File and S2 File).

### Prediction of Chemical Shifts of *De Novo*-Folded Models

Predicted CSs for the models generated from *de novo* folding were obtained by running PROSHIFT [63], SPARTA+ [64], SHIFTX [65], and SHIFTX2 [66]. When running PROSHIFT, the temperature and pH were set to 30°C and 6.0, respectively. SPARTA+, SHIFTX, and SHIFTX2 were run using default settings. During CS analysis, CSs obtained for Gly1 were disregarded. (see Protocol Capture in S1 File and S2 File).

### Selection of Low Energy Models in Contact with the Membrane

To maintain close contact between Ser3 and the membrane, all 10,000 models were filtered so that the Ser3 Cα of the filtered models were within the polar region of the RosettaMembrane implicit membrane environment. The remaining pool of 3,692 models was screened to filter out those models in which any peptide atoms found within 5 Å of any receptor atoms. All 3,692 of these models passed the filter and were further culled by keeping only those models whose Rosetta energies were within the top 10% of all 10,000 model energies, leaving a fully filtered pool of 355 models. This percentage was chosen after testing various ensemble sizes (S1 Table).

### Generation of Model Ensembles in Agreement with Experimental Chemical Shifts

**Fig 2:**
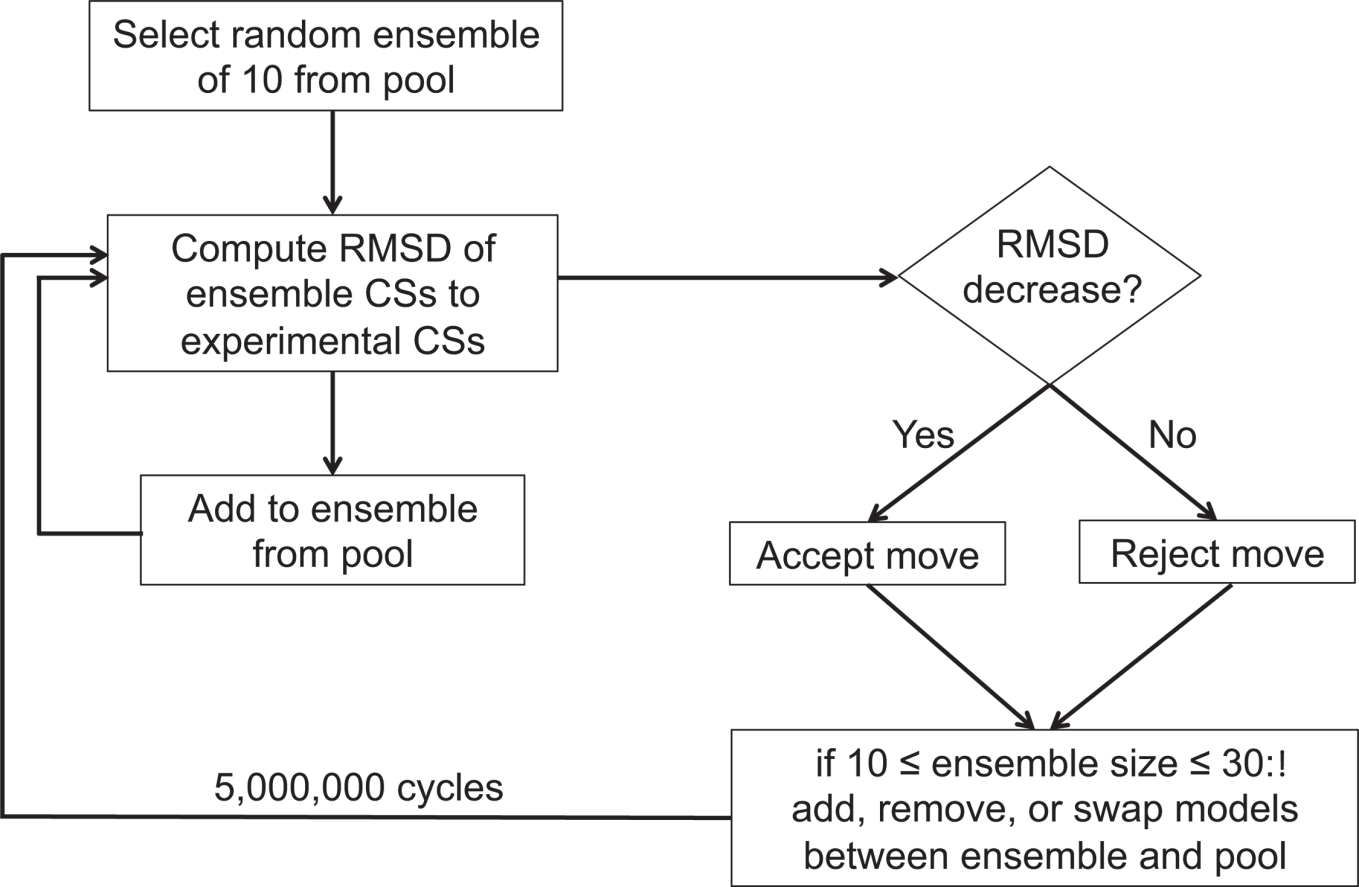
Outline of model ensemble selection algorithm. The flowchart outlines the process by which the agreement with experimental data is determined for an ensemble of models selected from a large pool.

Ensembles of 10–30 models consistent with the experimental CSs were constructed from the resulting low-energy pool according to the algorithm summarized in Fig. 2. PROSHIFT [63] was used to predict CSs for all models. The selection algorithm generates a random ensemble of 10 models. It then computes the average CS of each Cα, Cβ, CO, and Hα atom for which an experimental CS was determined (excluding those for Gly1). After all average CS values are determined, the root mean square deviation (RMSD) of the ensemble average-predicted CSs relative to the experimental CSs is calculated and reported. In order to avoid the average and RMSD being dominated by the larger magnitude of carbon CS values, carbon CSs were scaled down by a factor of 4. Next, the algorithm randomly chooses to either add another model from the bigger pool to the ensemble (if not at the specified maximum ensemble size of 30), swap models between the ensemble and the pool, or remove a model from the ensemble (if not at the minimum ensemble size of 10. The process is repeated for 5,000,000 cycles.

### Structural Analysis of Final Ensemble

The final ensemble of models was initially evaluated by the Protein Structure Validation Software suite (PSVS, http://psvs-1_5-dev.nesg.org/). The secondary structure information, including φ/ψ torsion angles, was obtained by running Define Secondary Structure of Proteins (DSSP [67], http://swift.cmbi.ru.nl/gv/dssp/). In addition, the DSSP analysis was modified to take into account polyproline II (PPII) helical structure using the same parameters presented by Adzhubei, Sternberg, and Makarav [68]. Briefly, residues were only assigned PPII structure if they met the following conditions: 1) formerly assigned random coil (−) by DSSP, 2) φ = −75 ± 29 degrees, 3) ψ = 145 ± 29 degrees, 3) conditions 1) and 2) were met for two sequential residues.

## Results

### Ghrelin Binds to Negatively Charged Membranes

**Fig 3:**
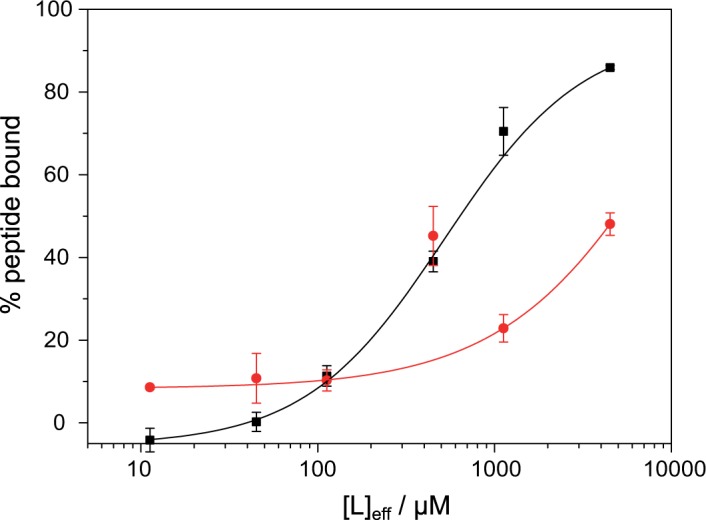
Binding isotherms of ghrelin and desacyl ghrelin to DMPC/DMPG membranes. The amount of bound ghrelin (black squares) and desacyl ghrelin (red circles) as a function of lipid concentration is given. The binding data were fitted according to Eq. (1).

First, binding of ghrelin and desacyl-ghrelin to DMPC/DMPG (5/1, mol/mol) membranes was measured using an ultracentrifugation assay. DMPG was used because of side reactions of DMPS with fluorescamine. Upon addition of sucrose loaded vesicles and ultracentrifugation, bound ghrelin co-precipitates with the liposomes and the percentage of bound peptide is measured with a fluorescamine assay as shown in Fig. 3. While >90% of the octanoylated ghrelin binds to the acidic liposomes with a K_D_ value of 520 ± 73 μM, much weaker binding is observed for desacyl ghrelin, which does not reach saturation even at 5 mM lipid concentration. This indicates the importance of the octanoyl modification. The K_D_-derived ΔG^0^ value for the binding of ghrelin to membrane surfaces is −29.9 kJ/mol. To confirm that the lipid membranes used in this study were in a lamellar liquid crystalline phase state, static ^31^P NMR spectra were recorded. All preparations showed the typical axially symmetric powder pattern with a Δσ = 45 ppm indicative of a lamellar membrane phase state (S3 Fig.).

**Fig 4:**
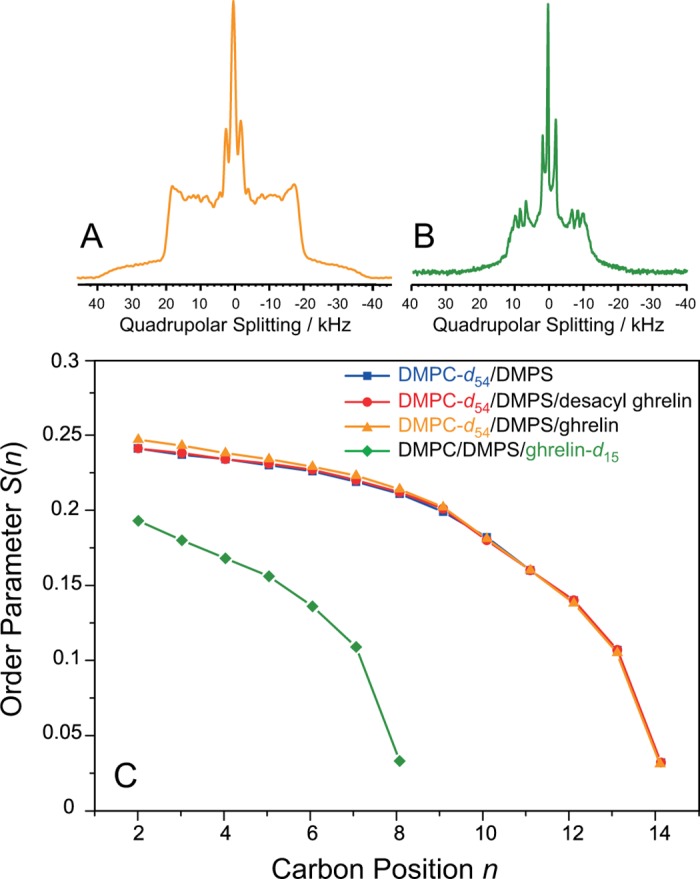
^2^H NMR spectra and order parameters of DMPC-*d*_54_/DMPS membranes in the absence and presence of ghrelin and deacylghrelin. ^2^H NMR spectra in DMPC-*d*_54_/DMPS membranes (5/1, mol/mol) in the presence of ghrelin (A) and ghrelin-*d*_15_ in DMPC/DMPS membranes (B). C) ^2^H NMR order parameters of DMPC-*d*_54_/DMPS (5:1, mol/mol) membranes in the presence or absence of ghrelin or desacylghreliln (1:30 protein to lipid molar ratio) at a temperature of 30°C and a buffer content of 35 wt%. Error bars of the ^2^H NMR order parameters are smaller than the symbol size.

To understand the dynamics of the membrane lipids and the lipid modification of membrane-associated ghrelin, the properties of the lipid chains in four different samples were compared: 1) pure DMPC-*d*_54_/DMPS, 2) DMPC-*d*_54_/DMPS/ghrelin at a 30:1 molar lipid-to-peptide molar ratio, 3) DMPC-*d*_54_/DMPS/desacyl-ghrelin, and 4) DMPC/DMPS/ghrelin-*d*_15_, where ghrelin featured a perdeuterated octanoyl-*d*_15_ chain at Ser3. This combination of samples allowed us to determine the effect of ghrelin on the bilayer properties of the host membrane. Typical ^2^H NMR spectra of the DMPC-*d*_54_/DMPS in the presence of ghrelin and the ghrelin-*d*_15_ component of the mixtures are shown in Fig. 4, panels A and B. The ^2^H NMR spectra show the typical superposition of Pake doublets, which is characteristic for the lamellar liquid crystalline phase state of the membrane. A small isotropic peak, as well as the bigger line width, indicates the presence of ghrelin. Isotropic lines in the ^2^H NMR spectra indicate that a small portion of the lipid or ghrelin may reside in structures with high curvature. The ^2^H NMR spectrum of ghrelin-*d*_15_ with a perdeuterated octanoyl chain also shows the features of a well-inserted peptide lipid chain, i.e., well dissolved Pake doublets. In addition, an isotropic peak that accounts for ~10% of the intensity is shown, indicating that about 10% of the octanoyl chain of ghrelin is isotropically mobile, or not inserted into the membrane.

From the ^2^H NMR powder spectra of the four samples mentioned above, the segmental chain order parameters were determined. Smoothed chain order parameter profiles showing the dependence of the order parameter on the position of the carbon segment in the acyl chain are presented in Fig. 4C. The segments are numbered consecutively starting at the carbonyl group of the lipid or the Cα of ghrelin’s Ser3. Striking differences between the chain order parameters of DMPC-*d*_54_ and ghrelin-*d*_15_ are observed. The ghrelin octanoyl chain shows significantly lower order parameters than the host membrane for all carbon positions. In contrast, the order parameters of the host membrane are very similar in the absence and presence of both ghrelin and desacyl ghrelin. Virtually no differences are observed for DMPC-*d*_54_/DMPS in the absence or presence of desacyl-ghrelin, confirming that there was no binding of the desacylated peptide to the membrane. Slightly higher order parameters are observed for the upper eight chain methylenes of the membrane in the presence of ghrelin. Using the mean torque model [69], the structural parameters of these lipid chains were calculated. The length of the DMPC chains in the mixture in the absence and presence of ghrelin was 11.1 Å and 11.3 Å, respectively. The length of the octanoyl chain of ghrelin was 4.8 Å.

### ^13^C Chemical Shifts Were Collected to Study the Structure of Membrane-Bound Ghrelin

**Fig 5:**
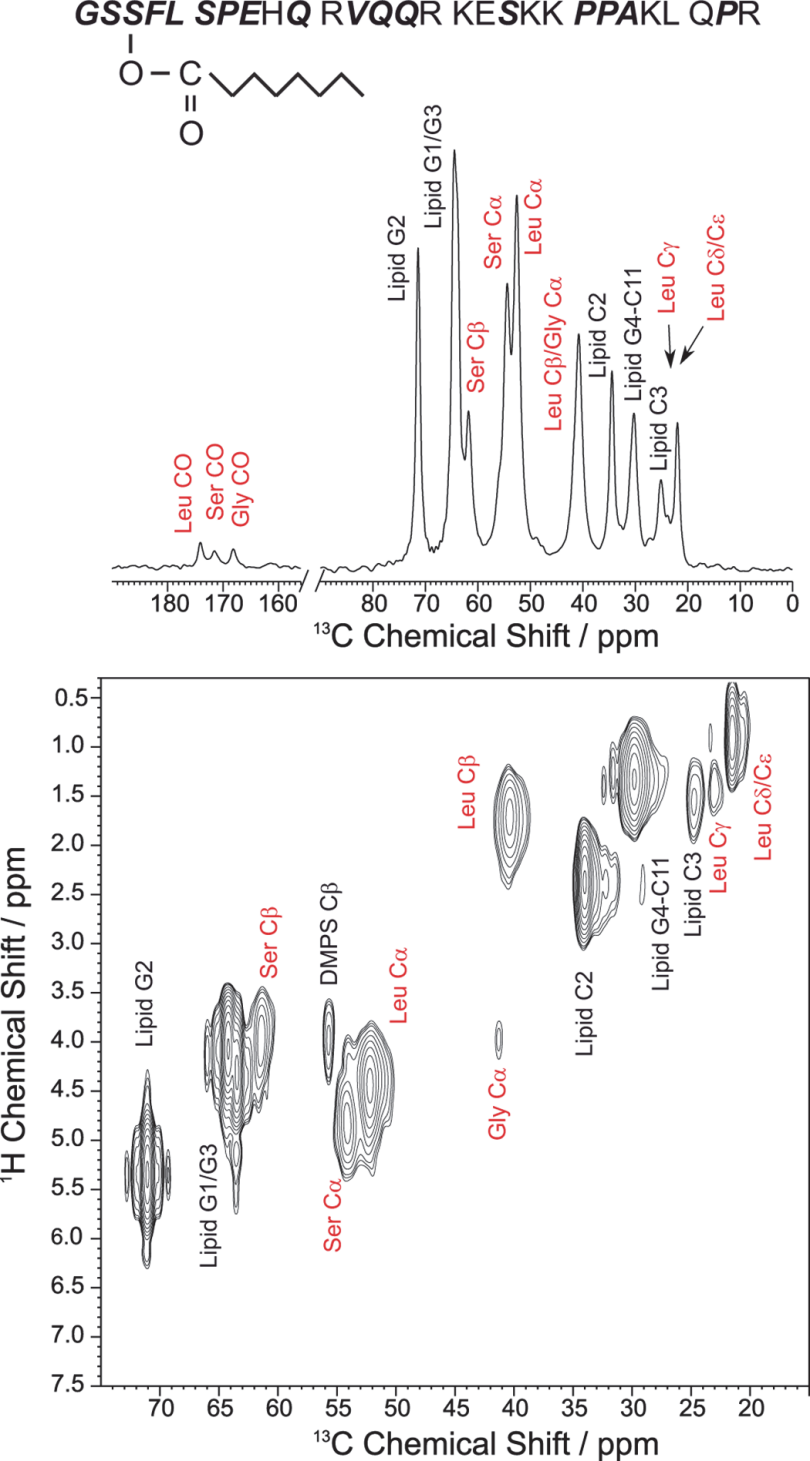
Ghrelin sequence showing the isotopically labeled amino acids of the different ghrelin molecules and ssNMR spectra of membrane-embedded ghrelin. Labeled amino acids are shown in bold italics (see S2 Table). A) ^13^C CP MAS NMR spectrum of ghrelin (with Gly1, Leu5, and Ser6 ^13^C/^15^N labeled) in DMPC-*d*_67_/DMPS-*d*_54_ (5:1, mol/mol) membranes at a ghrelin concentration of 3.3 mol%. B) ^1^H-^13^C MAS HetCor NMR spectrum of the same preparation, all at 30°C and a MAS frequency of 7 kHz.

Next, the secondary structure of membrane-bound ghrelin was investigated. To this end, six peptides with varying labeling scheme were synthesized (S2 Table). ^13^C MAS NMR measurements were carried out in DMPC/DMPS (5:1, mol/mol) membranes. A comprehensive set of directly excited ^13^C MAS NMR spectra, CP MAS spectra, and INEPT-based techniques were employed to find the most sensitive excitation scheme for membrane-bound ghrelin [70]; the CP MAS technique with a contact time of 700 μs provided the most sensitivity. A typical ^13^C CP MAS NMR spectrum of a ghrelin peptide in membranes is shown in Fig. 5A. As membrane-bound peptides often aggregate at high concentrations [52], the dependence of ghrelin CSs on peptide concentration was determined; ghrelin/lipid preparations of 1:30, 1:50, and 1:100 molar ratios were used. In all cases, there were no observable altered CSs, so a 1:30 ghrelin/lipid preparation was used for the remainder of this study.

**Fig 6:**
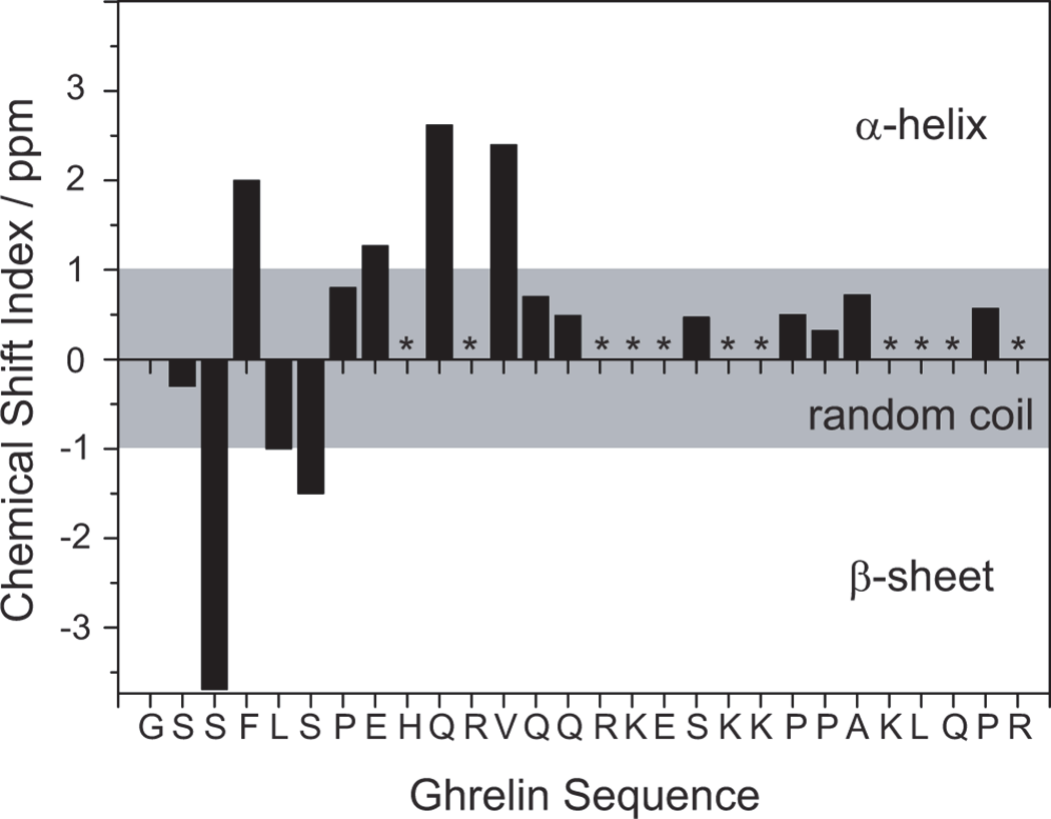
Chemical shift analysis of ghrelin based on MAS ssNMR data. The ^13^Cα–^13^Cβ CS values (chemical shift index) for reach residue are plotted. Positive values greater than 1 ppm indicate a tendency for α-helical structure, whereas values less than −1 ppm suggest β-sheet character. Amino acids with a CS index close to 0 ppm are considered to have no secondary structure. Asterisks indicate that no CSs were available for that residue.

To achieve the full assignments of the ghrelin signals, ^1^H–^13^C HetCor and ^13^C–^13^C PDSD experiments under magic angle spinning, or MAS (i.e., by ssNMR) were conducted. The basic connectivities within the labeled amino acid were determined in PDSD experiments using a mixing time of 50 ms. As membrane-associated ghrelin is relatively mobile (see below), the PDSD experiments were performed at −30°C. The high mobility of ghrelin helped in detecting ^1^H CSs in ^1^H–^13^C HetCor experiments, which were well-resolved, even without application of homo-nuclear decoupling. Typical peptide signals had a ^1^H MAS NMR line width of 0.3–0.4 ppm even under ssNMR conditions. A characteristic ^1^H–^13^C HetCor NMR spectrum of membrane-associated ghrelin is shown in Fig. 5B. A summary of the CS values determined for membrane-bound ghrelin is given in Table 1. The difference between ^13^Cα and ^13^Cβ values for determination of secondary structure are reported in Fig. 6. The ^13^C CP MAS NMR spectra, ^1^H–^13^C MAS HetCor and ^13^C–^13^C PDSD spectra for all membrane bound peptide variants are shown in S4 Fig.

**Table 1:** Chemical shifts measured for acylated ghrelin bound to DMPC/DMPC membranes (5/1, mol/mol) using MAS solid-state NMR.

Residue	CO	Cα	Cβ	Cγ	Cδ	Hα	Hβ	Hγ	Hδ
Gly1	167.0 ± 0.4	40.9 ± 0.2							
Ser2	172.1 ± 0.2	55.6 ± 0.5	62.5 ± 0.6						
Ser3		53.6 ± 0.1	63.3 ± 0.2			4.5± 0.3			
Phe4	172.1 ± 0.2	55.8 ± 1.2	37.0 ± 0.8						
Leu5	174.8 ± 0.4	51.9 ± 0.2	40.7 ± 0.5						
Ser6	169.3 ± 0.5	54.2 ± 0.5	61.2 ± 0.6						
Pro7	174.9 ± 1.2	61.2 ± 0.5	30.8 ± 1.7						
Glu8	174.1 ± 0.2	54.3 ± 0.9	25.8 ± 0.9						
Gln10	177.3± 0.3	55.4 ± 0.4	27.0 ± 0.0	34.4 ± 0.9		4.1± 0.3			
Val12	174.1 ± 0.2	60.3 ± 0.9	30.0± 0.3						
Gln13	173.4 ± 0.3	53.5 ± 0.2	27.0 ± 0.1			4.3± 0.3	2.0± 0.3		
Gln14	173.5± 0.3	53.4 ± 0.1	27.0 ± 0.1	33.7 ± 0.1		4.3± 0.3	2.1± 0.3	2.4± 0.3	
Ser18	171.8 ± 0.2	55.8 ± 0.1	61.3 ± 0.2			4.4± 0.3	3.9± 0.3		
Pro21	177.7± 0.3	59.0 ± 0.1	28.3 ± 0.0	24.8 ± 0.1	48.0± 0.3	4.7± 0.3		2.0± 0.3	3.8± 0.3
Pro22	173.7 ± 0.2	60.4 ± 0.2	29.4 ± 0.0	24.8± 0.3	47.9± 0.3	4.4± 0.3			
Ala23	175.5 ± 0.5	50.5 ± 0.6	17.0± 0.3						
Pro27	173.3 ± 0.0	60.8 ± 0.0	29.4 ± 0.1	24.8 ± 0.1	48.1± 0.3	4.4± 0.3	2.0± 0.3	2.0± 0.3	3.8± 0.3

* Gray cells indicate that these chemical shifts were used in structure determination.

### Dipolar Couplings Were Measured to Study the Dynamics of Membrane-Bound Ghrelin

**Fig 7:**
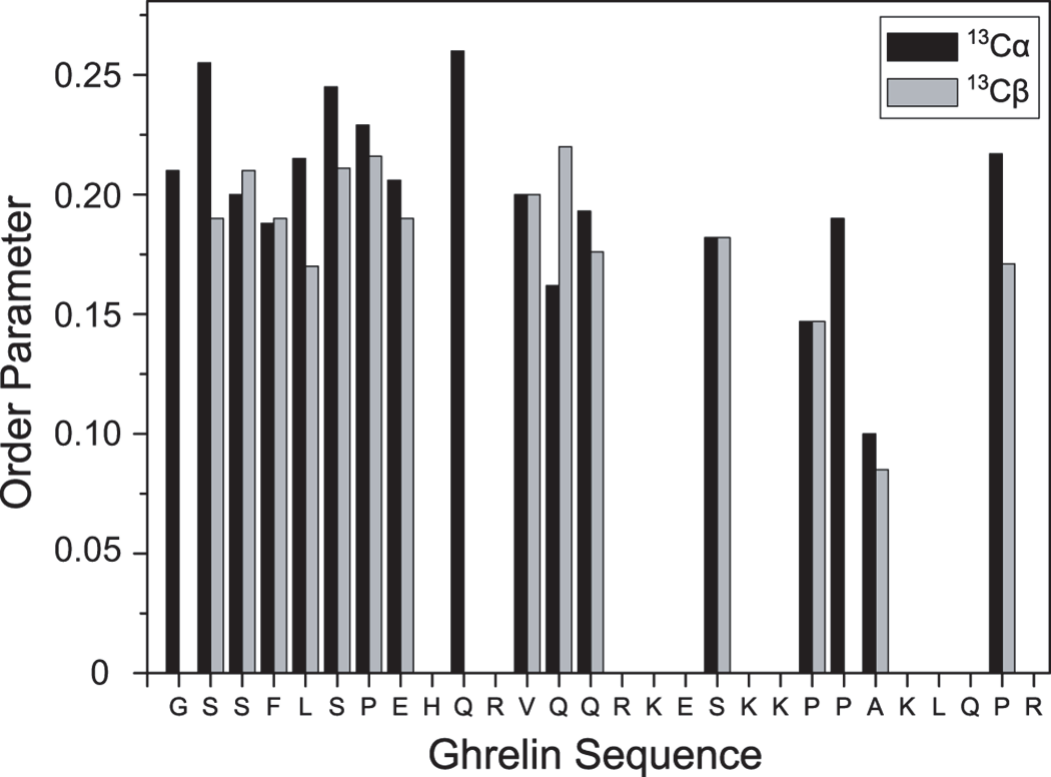
^1^H-^13^C order parameters of ghrelin bound to DMPC/DMPS membranes. Order parameters were determined for 3.3 mol% ghrelin bound to DMPC/DMPS membranes (5:1, mol/mol) at a temperature of 30°C and a water content of 35 wt%.

Next, the dynamics of membrane-associated ghrelin were studied via dipolar coupling measurements [50]. From the measurement of ^13^C–^1^H dipolar couplings, we determined the backbone and side chain order parameters needed to characterize the amplitude of motion for the C–H–bond vectors. A fully rigid C–H–bond exhibits the maximal dipolar coupling strength of 22.8 kHz, corresponding to an order parameter of 1. An order parameter value of 0 indicates fully isotropic motion, which is expressed by a vanishing dipolar coupling. Molecular motions with a given amplitude lead to partial averaging of the dipolar coupling strength and can be characterized by a specific order parameter. The ^1^H–^13^C order parameters sample all motions with correlation times shorter than ~10 μs [70] Overall, the order parameters for ghrelin in membranes are relatively low—around 0.2 for the backbone—with smaller values obtained for the side chains. There are no significant differences in the order parameters for residues 1–12. However, the order parameter of Ala23 was significantly lower, indicating a large increase in the motional amplitude at the C–terminus (Fig. 7).

### Ghrelin Interacts with Membrane via Ser3 and Phe4

**Fig 8:**
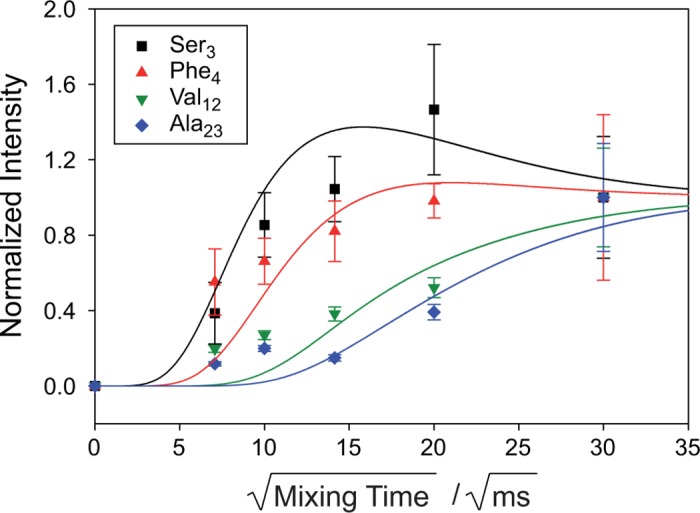
^1^H spin diffusion buildup curves of membrane-associated ghrelin. The plot shows the integral of the respective Cα position as a function of the square root of the mixing time, corrected for *T*_1_ relaxation and normalized to 1 for the longest spin diffusion time of 900 ms [53]. Spin diffusion spectra were determined for 3.3 mol% membrane-associated ghrelin in DMPC-*d*_67_/DMPS-*d*_54_ (5:1, mol/mol) at a D_2_O content of 35 wt%. Spin diffusion originates from the membrane’s glycerol and the PS headgroups. Solid lines represent best-fit simulations using a lattice model with a spin diffusion coefficient of *D* = 0.001 nm^2^/s and a distance between protons of 2 Å.

Finally, the membrane topology of ghrelin was investigated by measuring spin diffusion from the lipid into the peptide [53]. Ghrelin samples were prepared in DMPC−d_67_/DMPS−d_54_ membranes in the presence of D_2_O. Thus, spin diffusion originating from the glycerol backbone and the PS headgroup was detected in the ghrelin backbone. Typical spin diffusion curves for Ser3, Phe4, Val12, and Ala23 are shown in Fig. 8. At a mixing time of 0, all peptide magnetization was relaxed due to the T_2_ filter of 6 ms. However, as the mixing time increases, the intensity of the ghrelin signals also increases. Qualitatively, magnetization buildup is fastest in Ser3 and Phe4, while a significantly decreased magnetization buildup is detected for Val12 and Ala23. This means that Ser3 and Phe4 are in close proximity to the membrane surface, while Val12 and Ala23 have no direct membrane contact because spin diffusion has to migrate longer to reach these sites.

Magnetization buildup was also simulated using a simple lattice model for spin diffusion [54]. As the mobility of the lipids and ghrelin are comparable (see Figs. 4 and 7), a common spin diffusion coefficient of *D* = 0.001 nm^2^/s was used for spin diffusion within the lipid, from lipid to peptide, and within ghrelin. With these simple assumptions, the magnetization buildup could be modeled relatively well using a 2–Å spacing between neighboring spins. In the lattice model, spin diffusion from the lipid reaches the peptide sites in close proximity to the membrane surface, Ser3 and Phe4, in 3 and 4 steps, respectively. On the other hand, 6 to 8 steps are necessary for the magnetization to diffuse to residues Val12 and Ala23 indicating the increased distance of these residues from the membrane surface.

### PROSHIFT Predicts CS of *De Novo* Folded Ghrelin with Smallest Deviation from Experiment

In order to construct an ensemble of ghrelin models in agreement with the experimental CS data (Table 1), an appropriate method for predicting CSs based on the *de novo* folded models was needed. We tested four CS prediction tools: PROSHIFT [63], SHIFTX [65], SHIFTX2 [66], and SPARTA+ [64]. PROSHIFT employs an artificial neural network (ANN) trained on CS data from the Biological Magnetic Resonance Bank (BMRB). SHIFTX operates via a hybrid method, in which empirically derived CS hypersurfaces are combined with classical (i.e., Newtonian physics) or semi-classical equations for parameters, such as ring current, hydrogen bond, and solvent effects. SHIFTX2, like SHIFTX, employs structure-based concepts used by SHIFTX, but the algorithm also takes sequence homology information into account, as is done by SHIFTY [71]. SPARTA+ uses an ANN, but, being a newer method, the ANN was trained on an approximately two-fold larger protein database than was used for training the PROSHIFT ANN. We hypothesized that the fragment-based assembly in Rosetta samples the conformational space likely occupied by the biologically active peptide and that, therefore, some models within the final ensemble represent conformations that give rise to the observed CSs. Accordingly, one can argue that the CS prediction algorithm most suitable for this particular application should give the lowest CS-RMSD between experimental and predicted CS. Because not all of the CS prediction methods predict values for side chain atoms, including protons, only CO, Cα, Cβ, and Hα CSs were used in the determination of the CS-RMSD.

After using all four of the aforementioned methods to predict CSs for the 10,000 Rosetta-generated models, the CS-RMSD (in ppm) of each model to the experimental data was computed. The Rosetta score, or energy, was plotted against CS-RMSD, as determined by each CS prediction method (S5 Fig.). Surprisingly, it was found that PROSHIFT systematically created lower CS-RMSD values. Manual inspection of one selected model that agreed well with predicted CSs from all methods confirmed that more accurate CSs were predicted throughout the peptide and not located in one particular region (S3 Table).

Furthermore, the selection algorithm used to find the ensemble of models with the best overall agreement to the experimental CSs resulted in lower average RMSD values when the model CSs were predicted by PROSHIFT (S1 Table). After running the selection algorithm over model pools of various sizes, each with PROSHIFT, SHIFTX, SHIFTX2, or SPARTA(+)-predicted CSs, it was determined that, for this system, the top 10% of models by total Rosetta score that had Ser3 Cα atoms in proximity to the membrane plane struck the best compromise between favorable Rosetta energy and agreement with experimental CSs.

### The Final Structural Ensemble of Ghrelin is Highly Flexible

**Fig 9:**
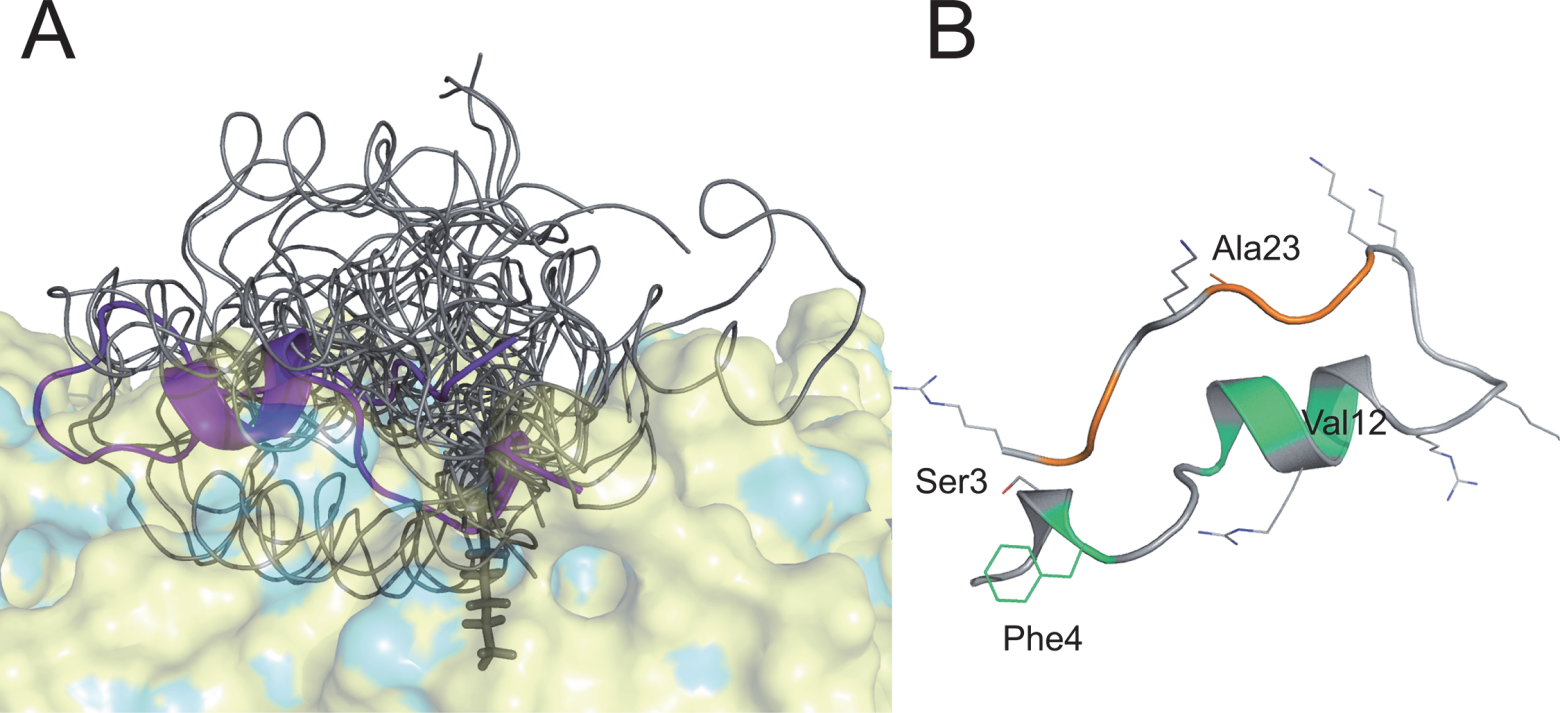
Structure of ghrelin based on MAS ssNMR chemical shift data. A) Final ensemble of ghrelin selected from the ensemble selection algorithm discussed in the main text. The Ser3 Cα of each model was superimposed on the others. The manually placed octanoyl side chain is shown as sticks. The model in Panel B is shown as solid cartoon. Residues predicted to be PPII helix (21–23 and 26–27) are colored in orange. Residues predicted to be helical according to the chemical shift analysis displayed in Fig. 6 (4, 8, 10, and 12) are colored in green. The rest of the ensemble is shown as ribbon (gray). The ensemble was manually positioned on the surface of a DMPC lipid bilayer (blue) to illustrate how the highly flexible ghrelin peptide might interact with the membrane. B) Model from the final ensemble. The color scheme is the same as the peptide (cartoon) in Panel A. Positively charged residues (Arg and Lys), Ser3, Phe4, Val12, and Ala23 are displayed as lines.

The final ensemble of 22 ghrelin models, which was selected according to the algorithm outlined in Fig. 2, had an RMSD of 0.4 ppm relative to the experimental CSs. However, the ensemble is highly flexible and mobile. The backbone RMSD to mean structure is 4.0 ± 0.8 Å (Table 2). There was no structural core by which the models could be aligned; therefore, the models’ Ser3 Cα were superimposed for visualization (Fig. 9).

**Table 2:** Statistics for restraints, structural calculations, and structural quality for final ensemble of ghrelin models.

**NMR distance restraints used during folding and refinement**	
Total restraints	55
Chemical shifts^a^	55
**Structural statistics**	
Number of models in ensemble	22
Deviations from idealized geometry	
Bond lengths (Å)	0.02
Bond angles (°)	0.7
Main chain RMSD to the mean structure (Å)	4.0 ± 0.8
Ensemble average RMSD to chemical shifts (ppm)	0.4
Ramachandran plot statistics (%)	
Most favored regions^b^, ^c^	95.2, 99.5
Additionally allowed regions^b^,^c^	4.8, 0.5

^*a*^ Chemical shifts were used during post-processing only; they were not used during fragment generation or *de novo* folding and refinement.

^*b*^ As determined by PROCHECK (http://www.ebi.ac.uk/thornton-srv/software/PROCHECK/).

^c^ As determined by MolProbity (http://molprobity.biochem.duke.edu).

**Fig 10:**
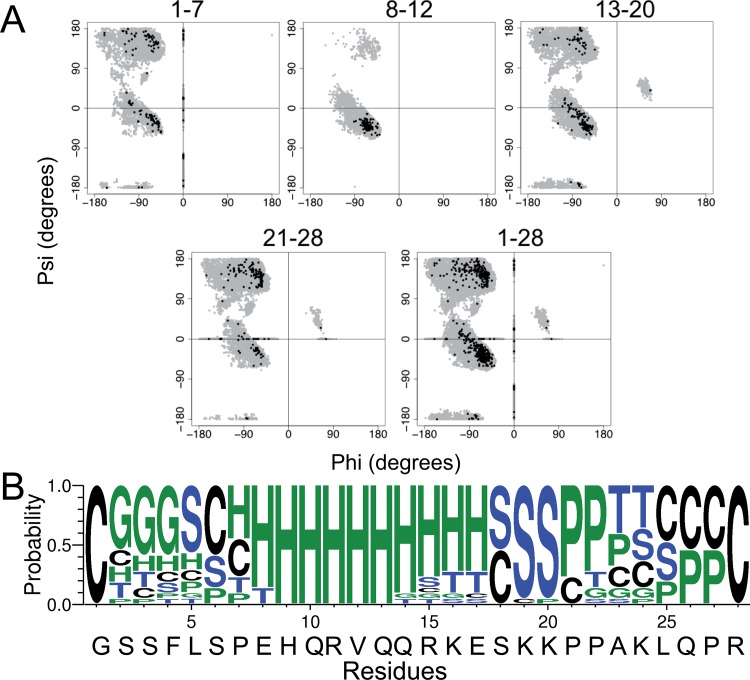
Secondary structure analysis of ghrelin. A) Ramachandran plots of various subsets of residues as labeled at the top of the plots. The torsion angles of all models generated in Rosetta (gray) and the final ensemble of models (black) are plotted. B) Weblogo (http://weblogo.threeplusone.com) of PPII-DSSP analysis of final ensemble of ghrelin models. Color key: black = random coil (C), blue = bend (S) or turn (T), and green = α-, 3_10_-, or PPII helix (H, G, or P, respectively).

Overall, the structural ensemble of membrane-associated ghrelin represents the high amount of molecular dynamics inferred from the NMR data. Notice that the peptide exhibits some α-helical core but no β-strand character. After further inspection of the Ramachandran plot generated for all 10,000 models, as well as for the final ensemble, it appears that the final ensemble may exhibit some polyproline II helical character, which would be found in the φ = −75° / ψ = 150° area (Fig. 10). Additionally, analysis of the φ / ψ angles using the PPII-DSSP method presented by Kabsch and Sander [67], residues 21–23 and 26–27 show significant PPII helical propensity (Fig. 10B). The α-helical core agrees well with the secondary structure prediction of ghrelin based on PSIPRED [72], JUFO [73], and SAM [74] (S2 Fig.). On the other hand, according to TALOS+ [75], which is based on the experimental CSs, the peptide, especially residues 21–23, is expected to be almost completely random coil. Ramachandran plots of residues 1–7, 8–12, 13–20, 21–28, and 21–28 indicate that the secondary structure of the final ensemble is not completely at odds with the secondary structure prediction or experimental CSs (Fig. 10A).

## Discussion

### Ghrelin Interacts with the Membrane via a Small Hydrophobic Cluster

According to our spin diffusion studies, ghrelin interacts with the membrane via residues Ser3 and Phe4, whose side chains and the octanoyl chain insert into the membrane (Fig. 8); this is also in agreement with solution NMR data performed in detergent micelles [27]. Due to the deuteration scheme of the membrane, ^1^H spin diffusion can only originate from the glycerol backbone and the polar headgroup, suggesting localization of the Phe side chain in this region. Generally speaking, the interface region of the membrane represents the preferred localization for membrane-bound lipidated peptides [76,77]. Spin diffusion into residues Val12 and Ala23 is significantly slower, implying that these residues have no membrane contact. It has been shown that due to Born repulsion, no direct molecular contact between cationic peptides and acidic membrane surfaces is established, typically, one water layer is found in between the peptide and the membrane surface [78]. Since this water is highly mobile, spin diffusion is substantially attenuated. Due to the highly dynamic ghrelin structure at the membrane surface and the fact that the octanoyl chain is in equilibrium between an inserted state (~90% of the time) and a desorbed state (~10% of the time) as determined from our binding assay, see Fig. 3, spin diffusion from the membrane into the peptide is significantly slower than what is observed for membrane proteins with a transmembrane segment [53].

The small hydrophobic cluster of amino acids of octanoylated Ser3, Phe4, and Leu5 account for about −13.4 kJ/mol [79,80] of the energy corresponding to the ghrelin-membrane interaction. At the lipid concentrations used in our experiments, this is insufficient for a permanent association with the membrane. Using a simple membrane partition model [29], this would only account for binding of ~8% of ghrelin. Clearly, a second mechanism is required for anchoring ghrelin to the membrane. This second mechanism is electrostatic attraction of the positively charged C-terminal two-thirds of the ghrelin sequence to the lipid headgroups. Indeed, ghrelin holds an electrostatic charge of +5.8 at pH 6, which was used for our studies to prevent the hydrolysis of the octanoyl chain. Numerous calculations based on the Gouy Chapman theory have been carried out to determine the electrostatic contribution to membrane binding of lipidated peptides [78]. For instance, pentalysine binds to a slightly negatively charged membrane, as in our case, with a Gibbs free energy of approximately −12 kJ/mol [81]. Together with the hydrophobic contribution from the N-terminus of ghrelin, we estimate a total membrane binding energy of Δ*G* ^0^ about −25 kJ/mol, which corresponds to approximately 90% of bound ghrelin using a simple partitioning model [29]. This corresponds relatively well with the measured value of −29.9 kJ/mol determined from the binding measurement.

### The Octanoyl Chain Might Play a Role in a Fine-Tuned Membrane Association Mechanism

Considering the observations made in this study, it is surprising that ghrelin is modified with an octanoyl chain and not with a longer lipid chain or prenyl groups, which provide much more favorable membrane anchors [29]. Although there is some disagreement about the exact hydrophopic contribution of ghrelin to membrane binding, most studies agree that desacyl-ghrelin does not significantly bind to membranes [23,27] and therefore, activates the receptor only in micromolar concentrations [19,23]. However, the short octanoyl chain is clearly not optimal for membrane binding. Bednarek, *et al*. showed that longer lipid chains and bulkier groups are also tolerated by the GHSR [19]. In addition, Matsumoto, *et al*. demonstrate that for activation of the receptor, the minimal linear fatty acid has to be a hexanoyl moiety, but the substitution of Ser(octanoyl) with the bulky amino acids, tryptophane or 1-naphthylalanine, are also accepted [82]. Apparently, the short ghrelin octanoyl chain is not optimized for the purpose of membrane binding but responsible for a fine-tuned membrane association mechanism, which catalyzes receptor binding and activation [21,23].

### Previous Studies of Membrane-Associated Ghrelin and Related Peptides Primarily Indicate α-Helical Structure

Earlier ^1^H NMR studies of acylated and desacylated ghrelin in aqueous solution at low pH indicate that both forms of the peptide are highly unstructured in water. Indeed, the poor dispersion of CSs, as well as the lack of nuclear Overhauser effects typically seen of α-helices and β-sheets support the CD data [22]. It is also possible that ghrelin experiences structural inter-conversion on a faster timescale than the NMR measurements, resulting in no detection of transient secondary structure. In work performed by Beevers and Kukol [24,83], a 10-ns MD simulation performed in water at constant temperature and neutral pH (preceded by 2 ns of simulated annealing MD, or SAMD) provided evidence that ghrelin may sample a helix from residues 7 to 13 in both environments. MD studies in DMPC bilayers for 15 ns, initiated with the energy-minimized final peptide from the previous 10-ns MD simulation in water, did not show any significant differences in secondary structure from the peptide in aqueous conditions. However, the presence of the membrane appeared to reduce ghrelin’s flexibility. Interestingly, the octanoyl side chain, while initially pointed to the lipid bilayer, did not anchor the peptide to the membrane. Instead, during the simulation, residues 15–18 served as contact points with the lipid headgroups [24,83].

CD spectroscopy of ghrelin and desacyl-ghrelin performed in aqueous solution (20 mM Tris buffer) and in 100% TFE at pH 7.4 provide experimental support for the MD studies, in that the acylated peptide exhibits 12% helical character in aqueous solution and in TFE. Desacyl-ghrelin, on the other hand, showed a significant increase in helical character when going from an aqueous environment (23%) to TFE (48%) [84].

In contrast to the MD simulations [24], Staes, *et al*. showed via a variety of biochemical assays that, while ghrelin and desacyl-ghrelin both electrostatically interact with the membrane, only acylated ghrelin penetrates into negatively charged membranes [23]. However, the interaction of ghrelin with membranes was not such that it otherwise significantly disturbed the membrane surface. The same authors also investigated the secondary structure of ghrelin and desacyl-ghrelin via *in silico* modeling and CD spectroscopy. Similar to previous MD studies [24], an α-helix spanning residues Pro7 to Ser18, which was flanked by two loops, for both acylated and desacylated ghrelin. The authors’ models were supported by CD data collected in water, dodecylphosphocholine micelles, SDS micelles, and TFE. For both forms of ghrelin, the helicity increased significantly in SDS micelles and TFE [23] A similar trend was observed for the prolactin releasing peptide (PrRP), another peptide that plays a role in food intake and body weight homeostasis [85]. In the case of PrRP, it was demonstrated that the peptide likely exists in a conformational equilibrium between α- and 3_10_-helix, and the helical propensity of the peptide is essential for its ability to activate the PrRP receptor, another GPCR. Another peptide that is involved in the regulation of appetite, galanin-like peptide, also shows nascent helical character, which may increase upon binding to galanin receptors [86]. More recently, the neuropeptide, substance P, was also found to have α-helical character in negatively charged SDS micelles and DMPG liposomes. However, in aqueous solution and in sub-micellar concentrations of SDS and DMPC liposomes, CD spectra indicate the presence of polyproline II (PPII) helix [87].

### The Ghrelin Model Ensemble Exhibits a Highly Flexible Structure Containing Some Polyproline II-, α- and 3_10_-Helix

Based on previous structural studies, structural characterization of related peptides, and secondary structure prediction based on the primary sequence (S2 Fig.), it was expected that our current ssNMR studies would point to a dynamic peptide having transient α- and/or 3_10_-helical character in conformational equilibrium. Interestingly, ^13^Cα −^13^Cβ CS values indicate helical propensity for residue 4, 8, 10, and 12, but according to CS index analysis with TALOS+ only Arg11 exhibits a small amount of helical propensity (S2 Fig.). This is in agreement with the high mobility inferred from the low order parameters that have been measured for the peptide (Fig. 7).

The final ensemble of Rosetta-generated models in best agreement with the experimental CSs provides a set of three-dimensional (3D) structures that allow for the visualization of the information obtained by NMR. It is important to note that, while the models are supported by the herein discussed ssNMR CS data, they may represent a speculative reflection of what occurs *in vivo*. As expected from the order parameters, we modeled a very loose conformational ensemble (Fig. 10). Interestingly, while Rosetta sampled φ/ψ torsion angles expected for all common secondary structures (i.e., α-helix and β-sheet), the final ensemble exhibits a strongly helical core with what initially appeared to be “random coil” in the N- and C-terminal region, in agreement with previous studies [1–3,23,24]. However, upon closer inspection of the Ramachandran plots and a modified DSSP analysis of these 22 models, it is probable that the final ensemble exhibits a small amount of 3_10_-helical character, as well as a significant amount of PPII helix, especially for Pro21-Ala23 and Gln26-Pro27 (Figs. 9 and 10). The helical character of ghrelin does appear to allow it to frequently adopt amphipathic conformations, thus allowing the basic residues to interact with the membrane’s polar headgroups (Fig. 9).

### Polyproline II Helical Conformation in Ghrelin May Play a Biologically Significant Role

Pure PPII helix is left-handed, is often characterized as a triangular prism, and has a helical pitch of 9.3 Å/turn; it contains φ and ψ angles of −75° and 145°, respectively. However, other amino acids and combinations of amino acids can form PPII helices [88]. Stapley and Creamer state that, in addition to Pro, Gln and positively charged residues having an increased probability of existing in PPII helices, Gly and aromatic residues show decreased probability [89] Other analyses of proteins of known structure agree that Gly and aromatic residues have low propensities to form PPII helices and that Pro appears most often, the increased observation of Gln and positively charged residues in PPII helices is disputed [90]. In addition to being sampled during protein folding and unfolding, PPII helical structure has been implicated in amyloid formation [91,92], nucleic acid binding [93], and muscle tissue elasticity [94]. Statistical analysis of a database of 274 non-homologous protein structures shows that, while only 2% of residues are found in PPII helices, more than half of all polypeptide chains contain PPII helix of at least three residues in length [89].

Our model ensemble of ghrelin, which is based on CS data from ssNMR, exhibits PPII helical character. Even though the models are based on experimental information, the nature of this character is implicated indirectly, as there do not to be examples of using isotropic CS information to describe PPII character. To our knowledge, methods used to predict nascent and flexible helical character, such as PPII helix, from NMR data rely on information from residual dipolar couplings [95–97] or chemical shift anisotropy [98]. Even so, ghrelin is the first membrane-associated peptide that appears to have PPII helical character for some residues in the presence of lipid bilayers. We point out that this character is likely transient and involves only short stretches of 2–3 residues. However, it is possible that, like the aforementioned peptides, ghrelin’s α-helical content increases and extends into the ten C-terminal residues when it binds to GHSR. However, the potential PPII helical character could allow for increased solvent accessibility while simultaneously providing for structural flexibility in areas such as flanking α-helices, linker regions, etc. Further, PPII helices have been found to be structural motifs involved in protein-protein interactions, which may result from their tendency to form amphipathic helices and to bind in a rapid and reversible fashion [68,99].

### Reliance of Peptide Fragment Selection on Chemical Shifts and Secondary Structure Prediction

While CSs can be used to guide fragment selection for *de novo* folding in Rosetta, we ultimately chose to utilize the original fragment selection protocol and filter by CS agreement after modeling was completed. Given that CS data for ghrelin is sparse, i.e., only for a subset of all residues secondary structure can be determined from the CSs, this protocol was chosen to prevent biases from residues with determined CSs on other regions of the peptide. In the case of ghrelin, Rosetta selects fragments based on agreement of CS for a subset of residues with little or no secondary structure information for other residues. When generating fragments for ghrelin, this led to a bias of β-hairpin fragments, which was not in agreement with other experimental data that pointed to a highly flexible and mobile peptide. We therefore opted to select fragments based on predicted secondary structure for all residues and filtered the models based on agreement of experimental CS later. Indeed, upon analysis of the secondary structure of fragments selected with and without experimental CSs, we see that the fragment selection scheme depends heavily on CS data when available, as is described in the literature.^36^ On the other hand, when CS data are not included in fragment selection, the secondary structure prediction of all residues is critical (S3 Fig.).

While our approach to modeling ghrelin with Rosetta is relatively quick and robust, the recent work of Vendruscolo and others [100–101], in which replica-averaged structural restraints based on NMR CSs are used to predict the structure of linker region of calmodulin [102], provides another option for CS-guided modeling of flexible peptides and protein regions. Unlike Rosetta, these methods use MD simulations, allowing for the study of conformational fluctuations as they occur over time.

### PROSHIFT Systematically Gives the Best Agreement Between Experimental and Predicted CS Values

In order to compare the Rosetta-generated models with the experimentally determined CSs, we tested four CS prediction methods: PROSHIFT, SPARTA+, SHIFTX, and SHIFTX2. While SPARTA+, SHIFTX, and SHIFTX2 performed similarly, PROSHIFT appears to be the best method for prediction of CSs for ghrelin (S2 Fig.). To rule out systematic error and artifacts, one low-energy model that had minimal deviations between predicted and experimental CSs was chosen for in-depth analysis (S3 Table and S6 Fig.). This was also carried out on a few randomly selected models (data not shown).

This result was somewhat surprising, given that PROSHIFT is an older method than the three to which it was compared. The reason for PROSHIFT’s superior performance is not obvious, especially considering that the same 55 (CO, Cα, Cβ, and Hα) CSs were used for all analysis. Our explanation for this phenomenon is that PROSHIFT might be less biased than other methods in predicting CSs for well-structured proteins with large amounts of secondary structure, thereby making it more suitable for prediction of CSs for peptides or intrinsically disordered proteins. We expect that, in this case, the “devil is in the details” and that there are multiple details in how the PROSHIFT ANNs were trained that leads to its superior performance for this system.

### A Combined ssNMR-Rosetta Protocol for Studying Structure and Dynamics of Flexible Peptides and Proteins

Due to the lack of regular inter-residue hydrogen bonding characteristic of α-helices and β-strands, it is likely that PPII helices are often categorized as “random coil” by secondary structure analysis software, such as DSSP. Furthermore, while PPII helices are difficult to detect directly by NMR [90] there have been attempts using CS data [103,104]. More generally, determining the structural ensemble that best represents sparse NMR CSs is especially challenging for biomolecules expected to be highly flexible and potentially unstructured. In addition to presenting a 3D structural ensemble of the biologically active form of ghrelin, we provide a novel, thorough method for predicting membrane-associated peptides, as well as for selecting a set of models based on ssNMR CSs. As NMR is often used to characterize protein unfolding and intrinsically unstructured proteins [94,95,104–105], we believe our approach of combing NMR with Rosetta and a Monte Carlo ensemble selection algorithm may be useful for future studies of other structurally flexible and mobile systems.

## Conclusions

To date, the results on the structure and dynamics of ghrelin have been controversial and inconclusive. In order to elucidate the mechanism by which ghrelin interacts with the membrane, as well as its 3D structure and its dynamics in the membrane environment, CSs and order parameter data were collected via MAS ssNMR. The primary sequence of ghrelin was then used to *de novo* fold the peptide in Rosetta using the RosettaMembrane energy functions. A final ensemble of models was then selected based on the CS data. Unlike other peptides that activate GPCRs and in contrast to previous studies of ghrelin, our model of ghrelin is extremely flexible (4-Å RMSD), and appears to undergo large-amplitude motions at the membrane surface while strongly sampling both α- and PPII helical character. This unique secondary structure may allow the peptide to adopt an amphipathic structure, which would bind electrostatically to the membrane. Finally, the protocol employed to fold ghrelin and select the final ensemble of models can be used to structurally characterize other flexible proteins and peptides for which only sparse CS data are available, including those that act in a lipid environment.

## Supporting Information

S1 Fig Sequence alignment of GHSR and GPCRs of known structure. The sequences of twenty GPCR templates of known structure and nineteen GHSR sequences were used manually aligned in Aline (http://crystal.scb.uwa.edu.au/charlie/software/aline/) such that gaps in the predicted TMH consensus ranges (dark gray helices) were minimized and conserved prolines (white triangles) and cysteines (open gray triangles) remained in alignment.
(EPS)

S2 Fig Secondary structure prediction of ghrelin. A) Secondary structure prediction for the primary sequence of ghrelin. B) Secondary structure composition of 3-mer and 9-mer amino acid fragments used in *de novo* folding. These fragments were generated based either on the primary sequence of the peptide alone (+CS and −CS). For all predictions, α-helices (H) are in green, β-strands (S) are in blue, and random coil (C) are in black.
(EPS)

S3 Fig ^31^P NMR spectra of DMPC/DMPC (5/1, mol/mol) membranes in the presence or absence of ghrelin or desacylghrelin at a molar lipid to peptide ratio of 30:1. Red curves show best fit line shape simulations.
(EPS)

S4 Fig ^13^C CP MAS NMR spectra, ^1^H-^13^C MAS HetCor and ^13^C-^13^C PDSD spectra for all peptide variants investigated in this study. This collection provides insight into the line width and quality of data used for the analysis.
(EPS)

S5 Fig Assessment of four chemical shift prediction methods. Score vs. RMSD (in ppm) plot, where the RMSD of each model’s predicted CSs to experimental values were computed. The RMSD was computed over each of the experimentally determined CSs, excluding the two CSs determined for Gly1.
(EPS)

S6 Fig In-depth analysis of chemical shifts for one model A) The CS_SPARTA+_, CS_SHIFTX_, and CS_SHIFTX2_ deviations from CS_experimental_ values (S3 Table) are plotted against CS_PROSHIFT_ deviations from CS_experimental_ values. All values are in ppm. B) Structure of the model chosen for in-depth analysis. While it was not in the final ensemble of models reported in this work, it was within the top 10% by Rosetta energy and the best or second-best model with respect to CS-RMSD relative to experimental CSs.
(EPS)

S1 File Further details about the generation of GHSR comparative model to define membrane location in Rosetta and the Resetta Protocol Capture are given. (DOCX)

S2 File Rosetta-related protocols as Python scripts. (TGZ)

S1 Table Ensemble average RMSDs (in ppm) resulting from filtering strategies. (DOC)

S2 Table Overview of ghrelin peptide constructs and labeling schemes. (DOC)

S3 Table Detailed analysis of low-RMSD model from set of filtered models in top 10% by score. (DOC)
