# Supplementary material for: Integrating Solid-State NMR and Computational Modeling to Investigate the Structure and Dynamics of Membrane-Associated Ghrelin
Source: PLoS One. 2015 Mar 24;10(3):e0122444. doi: 10.1371/journal.pone.0122444 (PMC4372444; doi:10.1371/journal.pone.0122444)
Supplement: S1 File — (DOCX) [file pone.0122444.s007.docx]

**Supplemental Information for:**

Integrating Solid-State NMR and Computational Modeling to Investigate the Structure and Dynamics of Membrane-Associated Ghrelin

Gerrit Vortmeier^1¶^, Stephanie H. DeLuca^2¶^, Sylvia Els-Heindl^3^, Constance Chollet^3^, Holger A. Scheidt^1^, Annette G. Beck-Sickinger^3^, Jens Meiler^2^, Daniel Huster^1^*

^1^ Institute of Medical Physics and Biophysics, University of Leipzig, Leipzig, Germany

^2^ Center for Structural Biology, Vanderbilt University, Nashville, Tennessee, USA 37232

^3^ Institute of Biochemistry, University of Leipzig, Leipzig, Germany

* Corresponding author

E-mail: [daniel.huster@medizin.uni-leipzig.de](mailto:daniel.huster@medizin.uni-leipzig.de)

^¶^ These authors contributed equally to this work.

**Generation of GHSR comparative model to define membrane location in Rosetta**

In order to fold a peptide at the membrane surface, for technical reasons, Rosetta requires at least one transmembrane span to define the location of the membrane. We decided to construct a comparative model of growth hormone secretagogue receptor 1a (GHSR) as we expect to leverage it in future studies. We then used this model to define the membrane location but ensured that no interaction between receptor and peptide occurs for the present study.

The comparative model was based on the sequence alignment in Fig. S1 and generated according to the protocol described previously.^1-4^ Briefly, GHSR amino acid sequences from nineteen species were aligned using ClustalW,^1-7^ resulting in a sequence alignment profile. Next, twenty GPCRs of known structure, henceforth referred to as templates, were structurally aligned in Mustang,^2,4,7-9^ which resulted in a structural alignment profile. Then, a profile-profile alignment was performed in ClustalW, and the resulting alignment was manually adjusted to minimize gaps in TMH regions and maximize alignment of regions conserved across GPCRs (Fig. S1).

The sequence of human GHSR was isolated from the final profile-profile alignment and threaded onto the backbone of the bovine rhodopsin structure (PDB: 1U19^9-11^). Next, all loops and areas of missing electron density (from alignment gaps) were built in for one hundred models using the Rosetta cyclic coordinate descent (CCD) loop modeling algorithm.^5,7^ The five lowest-energy models that did not contain chainbreaks were used as starting models for constructing extracellular loops (ECLs). For each starting structure, ECLs 1–3 were constructed for 185–200 models, resulting in a total of approximately 985 complete comparative models. Finally, the lowest energy model after building the ECLs was selected to define the membrane in the ghrelin folding protocol.

Protocol Capture

**Computational details**

All models were generated by independent simulations using Vanderbilt University’s Center for Structural Biology computing cluster and the university’s Advanced Computing Center for Research and Education (ACCRE). Computations were performed on a combination of AMD Opteron and Intel Nehalem processor nodes. All Rosetta-related protocols were conducted using Rosetta version 3.4. Python scripts referenced in protocol capture are provided as a compressed zip file (S2 File).

Comparative modeling

**FASTA file of GHSR1a**

>gi|38455410|ref|NP_940799.1| growth hormone secretagogue receptor

MWNATPSEEPGFNLTLADLDWDASPGNDSLGDELLQLFPAPLLAGVTATCVALFVVGIAGNLLTMLVVSR

FRELRTTTNLYLSSMAFSDLLIFLCMPLDLVRLWQYRPWNFGDLLCKLFQFVSESCTYATVLTITALSVE

RYFAICFPLRAKVVVTKGRVKLVIFVIWAVAFCSAGPIFVLVGVEHENGTDPWDTNECRPTEFAVRSGLL

TVMVWVSSIFFFLPVFCLTVLYSLIGRKLWRRRRGDAVVGASLRDQNHKQTVKMLAVVVFAFILCWLPFH

VGRYLFSKSFEPGSLEIAQISQYCNLVSFVLFYLSAAINPILYNIMSKKYRVAVFRLLGFEPFSQRKLST

LKDESSRAWTESSINT

**Transmembrane span prediction**

# Used HMMTOP, TMHMM, JUFO9D, and OCTOPUS servers. Also ran Meiler lab’s YUFOPM (Jeff Mendenhall):

yufopm GHSR1.fasta

**Threading of GHSR1a sequence on template structure**

# See Chapter III for sequence alignment information.

/sb/meiler/scripts/sequence_util/thread_pdb_from_alignment.py --template $TEMPLATE_NAME_FROM_ALIGNMENT --target $TARGET_NAME_FROM_ALIGNMENT --chain A --align_format clustal

**Preparation for making fragments**

make_fragments.pl –id GHSR1 –nofrags GHSR1.fasta

**Generating fragments with Rosetta fragment picker**

-in:file:fasta GHSR1.fasta

-in:path:database rosetta-3.4/rosetta_database

-in:file:vall rosetta-3.4/rosetta_tools/fragment_tools/vall.jul19.2011.gz

-frags:n_candidates 1000

-frags:n_frags 200

-frags:frag_sizes 3 9

-out:file:frag_prefix GHSR1_

-frags:scoring:config GHSR1.cfg

-in:file:checkpoint GHSR1.checkpoint

-frags:write_ca_coordinates

-frags:describe_fragments GHSR1_score

-frags:ss_pred GHSR1.psipred_ss2 psipred GHSR1.jufo_ss jufo GHSR1.rdb sam

**Generation of lipophilicity file**

rosetta_source/src/apps/public/membrane_abinitio/run_lips.pl <fasta file> <span file> <path to blastpgp> <path to nr database> <path to alignblast.pl script>

**GHSR1 disulfide definition**

116 198

**GHSR1 spanfile**

TM region consensus for Homo sapiens GHSR1a

7 366

antiparallel

n2c

43 66 43 66

80 100 80 100

120 140 120 140

162 181 162 181

212 233 212 233

263 282 263 282

304 325 304 325

**Fill in density by building loops**

#options file

-database /blue/meilerlab/apps/rosetta/rosetta-3.4/rosetta_database

-loops:timer #output time spent in seconds for each loop modeling job

-loops:fa_input #input structures are in full atom format

-in:fix_disulf GHSR1.disulfide #read disulfide connectivity information

-in:file:spanfile GHSR1.span

-in:file:lipofile GHSR1.lips4

-loops:relax fastrelax #does a minimization of the structure in the torsion space

-loops:extended true #force phi-psi angles to be set to 180 degrees independent of loop input file (recommended for production runs)

-loops:frag_sizes 9 3 1

-loops:frag_files GHSR1.200.9mers GHSR1.200.3mers none

-loops:remodel quick_ccd

-loops:refine refine_kic

-out:file:silent_struct_type binary #output file type

-membrane:no_interpolate_Mpair # membrane scoring specification

-membrane:Menv_penalties # turn on membrane penalty scores

-score:weights membrane_highres_Menv_smooth.wts

# command line

rosetta-3.4/rosetta_source/bin/loopmodel.default.linuxgccrelease -database rosetta-3.4/rosetta_database @fill_gaps.options -s GHSR1_on_"$TEMPLATE".pdb -loops:input_pdb GHSR1_on_"$TEMPLATE".pdb -loops:loop_file GHSR1_on_"$TEMPLATE"_init.loops -out:file:silent GHSR1_on_"$TEMPLATE"_fillgaps.out -out:file:scorefile GHSR1_on_"$TEMPLATE"_fillgaps.sc -nstruct 25

**Filter for building ECLs**

Filtered out models based on template 1U19 so that only took models with no chainbreaks within the top 10 by total score.

**Rebuilding extracellular loops**

#Options file

-database rosetta-3.4/rosetta_database

-loops:timer #output time spent in seconds for each loop modeling job

-loops:fa_input #input structures are in full atom format

-in:fix_disulf GHSR1.disulfide #read disulfide connectivity information

-in:file:spanfile GHSR1.span

-in:file:lipofile GHSR1.lips4

-in:detect_disulf true #NEW

-loops:relax fastrelax #does a minimization of the structure in the torsion space

-loops:extended true #force phi-psi angles to be set to 180 degrees independent of loop input file (recommended for production runs)

-loops:frag_sizes 9 3 1

-loops:frag_files GHSR1.200.9mers GHSR1.200.3mers none

-loops:ccd_closure

-loops:remodel quick_ccd

-loops:refine refine_kic

-ex1

-ex2

-relax:membrane #set up membrane environment for relax

-relax:fast

-out:file:silent_struct_type binary #output file type

-out:file:fullatom #output file will be fullatom

-membrane:no_interpolate_Mpair # membrane scoring specification

-membrane:Menv_penalties # turn on membrane penalty scores

-score:weights membrane_highres_Menv_smooth.wts

#Command

rosetta-3.4/rosetta_source/bin/loopmodel.default.linuxgccrelease -database rosetta-3.4/rosetta_database @rebuild_ecl.options -s GHSR1_on_"$TEMPLATE"_"$RANK".pdb -loops:input_pdb GHSR1_on_"$TEMPLATE"_"$RANK".pdb -loops:loop_file GHSR1_on_"$TEMPLATE".loops -out:file:silent GHSR1_on_"$TEMPLATE"_0"$RANK"_rebuild_ecl.out -out:file:scorefile GHSR1_on_"$TEMPLATE"_0"$RANK"_rebuild_ecl.sc -nstruct 20

**Selecting final model**

The final model was selected by choosing the lowest scoring model overall.

Folding of ghrelin in the Rosetta membrane environment

**FASTA file**

>GHSRg_renumber

APLLAGVTATCVALFVVGIAGNLLTMLVVSRFRELRTTTNLYLSSMAFSDLLIFLCMPLDLVRLWQYRPWNFGDLLCKLFQFVSESCTYATVLTITALSVERYFAICFPLRAKVVVTKGRVKLVIFVIWAVAFCSAGPIFVLVGVEHENGTDPWDTNECRPTEFAVRSGLLTVMVWVSSIFFFLPVFCLTVLYSLIGRKLWRRRRGDAVVGASLRDQNHKQTVKMLAVVVFAFILCWLPFHVGRYLFSKSFEPGSLEIAQISQYCNLVSFVLFYLSAAINPILYNIMSKKYRVAVFRLLGFGSSFLSPEHQRVQQRKESKKPPAKLQPR

**Making fragments**

See above section on making fragments for the receptor.

**Spanfile**

TM region consensus for Homo sapiens GHSR1 with ghrelin

7 329

antiparallel

n2c

4 27 4 27

41 61 41 61

81 101 81 101

123 142 123 142

173 194 173 194

224 243 224 243

265 286 265 286

**Lipophilicity file**

Generated as before but with the spanfile directly above.

**Rigid file (for Topology Broker)**

RIGID 1 301

**Topology broker setup file**

CLAIMER MembraneTopologyClaimer

END_CLAIMER

CLAIMER RigidChunkClaimer

NO_USE_INPUT_POSE

PDB receptor.pdb

REGION_FILE GHSRg.rigid

END_CLAIMER

**Options file for de novo folding**

-in

-file

-native receptor.pdb

-fasta GHSRg.fasta

-frag3 GHSRg.200.3mers

-frag9 GHSRg.200.9mers

-spanfile GHSRg.span

-lipofile GHSRg.lips4

-residues

-patch_selectors CENTROID_HA

-broker

-setup GHSRg.tpb

-run

-protocol broker

-score

-find_neighbors_3dgrid

-weights membrane_highres_Menv_smooth

-membrane

-no_interpolate_Mpair

-Menv_penalties

-abinitio

-membrane

-rg_reweight 0.00

-stage2_patch score_membrane_s2.wts_patch

-stage3a_patch score_membrane_s3a.wts_patch

-stage3b_patch score_membrane_s3b.wts_patch

-stage4_patch score_membrane_s4.wts_patch

-relax

-membrane

-fast

-ex1

-ex2

-out

-output

-file

-fullatom

-silent_struct_type binary

-overwrite

**De novo command line**

rosetta-3.4/rosetta_source/bin/minirosetta.static.linuxgccrelease -database rosetta-3.4/rosetta_database/ @fold_GHSRg.flags -out::nstruct ${NSTRUCT} -out:file:silent GHSRg.out -out:file:scorefile output/GHSRg.sc

Analysis and ensemble selection

**Filter by proximity to membrane**

#XML file

<dock_design>

    <SCOREFXNS> #defines non-standard score functions

    </SCOREFXNS>

    <FILTERS>

        <MembraneDepth name="membrane_depth" residue=304 depth_lb=48 depth_ub=60/> ### **this** covers polar region. Membrane is 0-60 (inner to outer)

    </FILTERS>

    <MOVERS>

    </MOVERS>

    <PROTOCOLS>

        <Add filter_name=membrane_depth/>

    </PROTOCOLS>

</dock_design>

#command

Rosetta/main/source/bin/rosetta_scripts.mpi.linuxgccrelease -database Rosetta/main/database/ -in:file:l pdb.ls -parser:protocol test.xml -out:file:score_only -in:file:spanfile GHSRg.span -in:file:lipofile GHSRg.lips4 -membrane:no_interpolate_Mpair -membrane:Menv_penalties -out:file:scorefile MembraneDepth.sc -out:no_nstruct_label >& MembraneDepth.log &

**Run PROSHIFT and format for further analysis**

proshift.exe ${pdb} ${pdb}.pro 303 6

grep SHIFT ${pro} | grep -v PROSHIFT > ${pro}.cs

**Run SPARTA+ and format for further analysis**

sparta/SPARTA+/sparta+ -in input.pdb -ref GHSRg.tab -out outfile.out -outS outfile.outstruct -outCS outfile.outcs -offset

tail -n55 outfile.outcs > outfile.outcs.tmp

cat outfile.outcs.tmp | awk '{print("SHIFT "NR"\t"$3"\t"$2" A\t"$1"\t"$6"\tppm +- "$9" ppm")}' > final.sparta.cs.out

**Run SHIFTX**

shiftx/./shiftx 1 ${pdb} ${pdb}.shiftx >& ${pdb}_shiftx.log

head -n30 ${pdb}.shiftx | tail -n28 > ${pdb}.shiftx.tmp

ls *.shiftx.tmp > shiftx.ls

./shiftx_to_proshift.py -i shiftx.ls --suffix cs –shiftx

**Run SHIFTX2**

python shiftx2-v107-linux/shiftx2.py -i ${pdb} -f TABULAR -p 6 -t 303 >& ${pdb}_shiftx2.log

head -n30 ${pdb}.cs | tail -n28 > ${pdb}.cs.shiftx2

ls *.shiftx2 > shiftx2.ls

./shiftx_to_proshift.py -i shiftx2.ls --suffix cs --shiftx2

**Compare predicted chemical shifts to experimental chemical shifts**

# list all predicted chemical shift files (one method at a time)

ls *.cs > cs.ls

awk '{system("./compare_cs.py --exp_cs GHSRg.tab.sd --pred_cs " $1 " --outfile " $1 ".out --summary --no_sd --carbon_scale_factor 0.25")}' cs.ls

ls *.cs.out.summary >> all_outputs.ls

foreach file (`cat all_outputs.ls`)

grep -H -v MaxDiff ${file} | awk '{split($1,a,":");print(a[1]"\t"a[2]"\t"$2"\t"$3"\t"$4"\t"$5"\t"$6"\t"$7"\t"$8"\t"$9"\t"$10"\t"$11"\t"$12"\t"$13"\t"$14"\t"$15"\t"$16)}' | awk '{split($1,a,".");print(a[1]"\t"$2"\t"$3"\t"$4"\t"$5"\t"$6"\t"$7"\t"$8"\t"$9"\t"$10"\t"$11"\t"$12"\t"$13"\t"$14"\t"$15"\t"$16)}' >> all_outputs.txt

end

echo "PDB #yes #no AvgDiff sdDiff MaxDiff MaxRes# MaxResn MaxAtom MaxExpCS MaxExpLB MaxExpUB MaxProCS MaxProLB MaxProUB RMSD" > GHSRg_compare_cs.out

cat all_outputs.txt >> GHSRg_compare_cs.out

**Input experimental chemical shifts**

# 1 G C 167.040 0.4

# 1 G CA 40.900 0.2

2 S C 172.100 0.2

2 S CA 55.600 0.5

2 S CB 62.500 0.6

3 S CA 53.580 0.10

3 S CB 63.270 0.21

3 S HA 4.488 9999

4 F C 172.100 0.2

4 F CA 55.800 1.2

4 F CB 37.000 0.8

5 L C 174.800 0.4

5 L CA 51.900 0.2

5 L CB 40.700 0.5

6 S C 169.300 0.5

6 S CA 54.250 0.5

6 S CB 61.250 0.6

7 P C 174.900 1.2

7 P CA 61.250 0.5

7 P CB 30.800 1.7

8 E C 174.100 0.2

8 E CA 54.300 0.9

8 E CB 25.800 0.9

10 Q C 177.300 9999

10 Q CA 55.420 0.35

10 Q CB 27.010 0.02

10 Q HA 4.130 9999

12 V C 174.100 0.2

12 V CA 60.300 0.9

12 V CB 30.000 9999

13 Q C 173.350 0.26

13 Q CA 53.530 0.18

13 Q CB 26.950 0.12

13 Q HA 4.310 9999

14 Q C 173.500 9999

14 Q CA 53.440 0.14

14 Q CB 26.950 0.05

14 Q HA 4.296 9999

18 S C 171.760 0.16

18 S CA 55.800 0.1

18 S CB 61.300 0.2

18 S HA 4.440 9999

21 P C 177.700 9999

21 P CA 58.980 0.09

21 P CB 28.320 0.04

21 P HA 4.720 9999

22 P C 173.670 0.18

22 P CA 60.430 0.25

22 P CB 29.410 0.03

22 P HA 4.440 9999

23 A C 175.500 0.5

23 A CA 50.500 0.6

23 A CB 17.000 9999

27 P C 173.320 0.03

27 P CA 60.770 0.021

27 P CB 29.420 0.14

27 P HA 4.430 9999

**Run ensemble selection script**

./find_best_ensemble.py --ncycles 5000000 --min_ensemble_size 10 --max_ensemble_size 30 --outfile outfile.out /directory/to/predicted/cs/in/proshift/format/ending/in/*.cs

Run DSSP and compute phi/psi angles for models

./run_dssp.py -i pdb.ls --all all.out

Find polyproline II helix residues

Ls *.dssp > dssp.ls

foreach file ( `cat dssp.ls` )

awk '{if(($3>=-104.0 && $3<=-46.0) && ($4>=116.0 && $4<=174.0) && ($2=="-"))print}' ${file} > ${file}.pp2

*end*

**References**

(1) Combs, S. A.; DeLuca, S. L.; DeLuca, S. H.; Lemmon, G. H.; Nguyen, E. D.; Willis, J. R.; Sheehan, J. H.; Meiler, J. Small-Molecule Ligand Docking into Comparative Models with Rosetta. *Nature Protocols* **2013**, *8*, 1277-1298.

(2) Rathmann, D.; Lindner, D.; DeLuca, S. H.; Kaufmann, K. W.; Meiler, J.; Beck-Sickinger, A. G. Ligand-Mimicking Receptor Variant Discloses Binding and Activation Mode of Prolactin-Releasing Peptide. *J. Biol. Chem.* **2012**, *287*, 32181–32194.

(3) Meiler, J. PROSHIFT: Protein Chemical Shift Prediction Using Artificial Neural Networks. *J. Biomol. NMR* **2003**, *26*, 25–37.

(4) Gregory, K. J.; Nguyen, E. D.; Reiff, S. D.; Squire, E. F.; Stauffer, S. R.; Lindsley, C. W.; Meiler, J.; Conn, P. J. Probing the Metabotropic Glutamate Receptor 5 (mGlu5) Positive Allosteric Modulator (PAM) Binding Pocket: Discovery of Point Mutations That Engender a “Molecular Switch” in PAM Pharmacology. *Molec. Pharmacol.* **2013**, *83*, 991–1006.

(5) Canutescu, A.; Shelenkov, A.; Dunbrack, R. A Graph-Theory Algorithm for Rapid Protein Side-Chain Prediction. *Protein Sci.* **2003**, *12*, 2001–2014.

(6) Neal, S. Rapid and Accurate Calculation of Protein 1H, 13C and 15N Chemical Shifts. *J. Biomol. NMR* **2003**, *26*, 215–240.

(7) Thompson, J. D.; Higgins, D. G.; Gibson, T. J. CLUSTALW: Improving the Sensitivity of Progressive Multiple Sequence Alignment Through Sequence Weighting, Position-Specific Gap Penalties and Weight Matrix Choice. *Nucl. Acids Res.* **1994**, *22*, 4673–4680.

(8) Han, B.; Liu, Y.; Ginzinger, S. W.; Wishart, D. S. SHIFTX2: Significantly Improved Protein Chemical Shift Prediction. *J. Biomol. NMR* **2011**, *50*, 43–57.

(9) Konagurthu, A. S.; Whisstock, J. C.; Stuckey, P. J.; Lesk, A. M. MUSTANG: a Multiple Structural Alignment Algorithm. *Prot. Struct. Funct. Bioinfo.* **2006**, *64*, 559–574.

(10) Okada, T.; Sugihara, M.; Bondar, A.-N.; Elstner, M.; Entel, P.; Buss, V. The Retinal Conformation and Its Environment in Rhodopsin in Light of a New 2.2 Å Crystal Structure. *J. Mol. Biol.* **2004**, *342*, 571–583.

(11) Shen, Y.; Bax, A. SPARTA+: A Modest Improvement in Empirical NMR Chemical Shift Prediction by Means of an Artificial Neural Network. *J. Biomol. NMR* **2010**, *48*, 13–22.

(12) Bond, C. S.; Schüttelkopf, A. W. ALINE: a WYSIWYG Protein-Sequence Alignment Editor for Publication-Quality Alignments. *Acta Crystallogr. D Biol. Crystallogr.* **2009**, *65*, 510–512.
