# Supplementary material for: Integrating Solid-State NMR and Computational Modeling to Investigate the Structure and Dynamics of Membrane-Associated Ghrelin
Source: PLoS One. 2015 Mar 24;10(3):e0122444. doi: 10.1371/journal.pone.0122444 (PMC4372444; doi:10.1371/journal.pone.0122444)
Supplement: S2 File — (TGZ) [file pone.0122444.s008.tgz › ghrelin/folding_analysis/PSVS_analysis/phi_psi_plot_lnk.html]

Protein Structure Quality Analysis Result


Text output from PDBStat of phi psi order

the pdf file of S(phi)~Residue\_number Plot

the postscript file of S(phi)~Residue\_number Plot

JPEG image of S(phi)~Residue\_number Plot

the pdf file of S(psi)~Residue\_number Plot

the postscript file of S(psi)~Residue\_number Plot

JPEG image of S(psi)~Residue\_number Plot

  
