# Supplementary material for: Integrating Solid-State NMR and Computational Modeling to Investigate the Structure and Dynamics of Membrane-Associated Ghrelin
Source: PLoS One. 2015 Mar 24;10(3):e0122444. doi: 10.1371/journal.pone.0122444 (PMC4372444; doi:10.1371/journal.pone.0122444)
Supplement: S2 File — (TGZ) [file pone.0122444.s008.tgz › ghrelin/folding_analysis/PSVS_analysis/rmsd_lnk.html]

Protein Structure Quality Analysis Result


Text report of backbone and heavy atom RMSD for ordered regions

Text report of backbone RMSD for entire protein

Text report of heavy atom RMSD for entire protein

Summary of heavy atom and backbone RMSDs over the whole protein and ordered residues

  
