# Supplementary material for: Integrating Solid-State NMR and Computational Modeling to Investigate the Structure and Dynamics of Membrane-Associated Ghrelin
Source: PLoS One. 2015 Mar 24;10(3):e0122444. doi: 10.1371/journal.pone.0122444 (PMC4372444; doi:10.1371/journal.pone.0122444)
Supplement: S2 File — (TGZ) [file pone.0122444.s008.tgz › ghrelin/folding_analysis/PSVS_analysis/toc_a.html]

**PSVS Analysis Results(V1.5)**

| Software Environment for PSVS |
| Warning message from PSVS |
| Concise report of structure quality analysis |
| Summary of results of this analysis |
| Plot of S(phi)|S(psi) v/s Residue number |
| Superimposed Coordinates |
| Table of Backbone and Heavy Atom RMSD |
| Ramachandran Plot for all models |
| Residue Properties for all models |
| Model Secondary Structures from Procheck |
| Ramachandran Plots for each residue from Procheck |
| Ramachandran analysis for each residue from Richardson lab's Molprobity |
| Chi1-Chi2 Plots for each residue |
| Global quality scores over the whole protein |
| Procheck backbone G-factors for each residue |
| Procheck all dihedral angles G-factors for each residue |
| Verify3D Score over a window of 7 residues |
| ProsaII Score over a window of 7 residues |
| VdW violations from MAGE |
| Results from PDB validation software |
| Archive of results |
