# Supplementary material for: Integrating Solid-State NMR and Computational Modeling to Investigate the Structure and Dynamics of Membrane-Associated Ghrelin
Source: PLoS One. 2015 Mar 24;10(3):e0122444. doi: 10.1371/journal.pone.0122444 (PMC4372444; doi:10.1371/journal.pone.0122444)
Supplement: S2 File — (TGZ) [file pone.0122444.s008.tgz › ghrelin/folding_analysis/PSVS_analysis/GHSRg_top1000pro_01_ramachand.pdf]

PROCHECK-NMR

Ramachandran Plot

GHSRg_top1000pro (22 models)

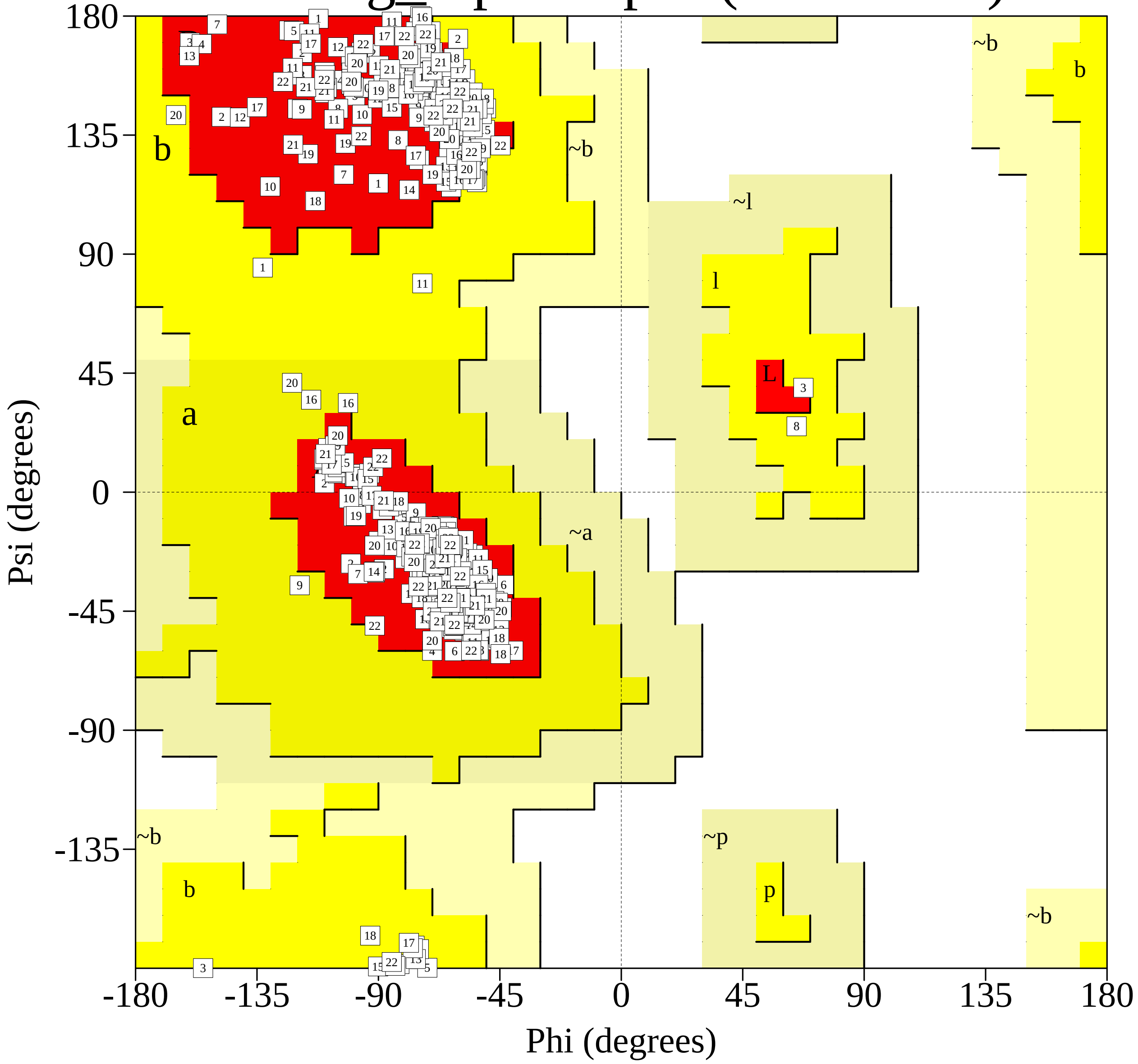

Plot statistics

Residues in most favoured regions [A,B,L]	461	95.2%
Residues in additional allowed regions [a,b,l,p]	23	4.8%
Residues in generously allowed regions [~a,~b,~l,~p]	0	0.0%
Residues in disallowed regions	0	0.0%
	----	------
Number of non-glycine and non-proline residues	484	100.0%
Number of end-residues (excl. Gly and Pro)	22	
Number of glycine residues (shown as triangles)	22	
Number of proline residues	88	

Total number of residues	616	

Based on an analysis of 118 structures of resolution of at least 2.0 Angstroms and R-factor no greater than 20%, a good quality model would be expected to have over 90% in the most favoured regions.
Model numbers shown inside each data point.

GHSRg_top1000pro_01_ramachand.ps
