# Supplementary material for: Integrating Solid-State NMR and Computational Modeling to Investigate the Structure and Dynamics of Membrane-Associated Ghrelin
Source: PLoS One. 2015 Mar 24;10(3):e0122444. doi: 10.1371/journal.pone.0122444 (PMC4372444; doi:10.1371/journal.pone.0122444)
Supplement: S2 File — (TGZ) [file pone.0122444.s008.tgz › ghrelin/folding_analysis/PSVS_analysis/GHSRg_top1000pro_results_summary.html]

Summary of structure quality factors


# Summary of structure quality factors

---

  

Analyses performed for all residues.

|  |
| --- |
|  |
| Total structures computed | currently unknown |
| Number of structures used | 22 |
  
|  |
| RMSD Values |
|  | all | orderede | Selectedf |
| All backbone atoms | 4.0 Å | 1.3 Å | 4.0 Å |
| All heavy atoms | 5.3 Å | 2.5 Å | 5.3 Å |
|  |
| Structure Quality Factors - overall statistics |
|  | Mean score | SD | Z-score g |
| Procheck G-factor e (phi / psi only) | 0.13 | N/A | 0.83 |
| Procheck G-factor e (all dihedral angles) | 0.29 | N/A | 1.71 |
| Verify3D | 0.24 | 0.0668 | -3.53 |
| ProsaII (-ve) | 0.60 | 0.1512 | -0.21 |
| MolProbity clashscore | 3.01 | 3.0681 | 1.01 |
|  |
| Ramachandran Plot Summary from Procheck f |
| Most favoured regions | 95.2% |
| Additionally allowed regions | 4.8% |
| Generously allowed regions | 0.0% |
| Disallowed regions | 0.0% |
|  |
| Ramachandran Plot Statistics from Richardson's lab |
| Most favoured regions | 99.5% |
| Allowed regions | 0.5% |
| Disallowed regions | 0% |

  

---

e Residues with sum of phi and psi order parameters > 1.8  
  
*Ordered residue ranges:*   
f Residues selected based on: all residues  
  
*Selected residue ranges: all*  
  
g With respect to mean and standard deviation for for a set of 252 X-ray structures < 500 residues, of resolution <= 1.80 Å, R-factor <= 0.25 and R-free <= 0.28; a positive value indicates a 'better' score  
  
Generated using PSVS 1.5  
