# Supplementary material for: Integrating Solid-State NMR and Computational Modeling to Investigate the Structure and Dynamics of Membrane-Associated Ghrelin
Source: PLoS One. 2015 Mar 24;10(3):e0122444. doi: 10.1371/journal.pone.0122444 (PMC4372444; doi:10.1371/journal.pone.0122444)
Supplement: S2 File — (TGZ) [file pone.0122444.s008.tgz › ghrelin/folding_analysis/PSVS_analysis/richardson_rama.pdf]

MolProbity Ramachandran analysis

GHSRg_top1000pro.pdb, all models

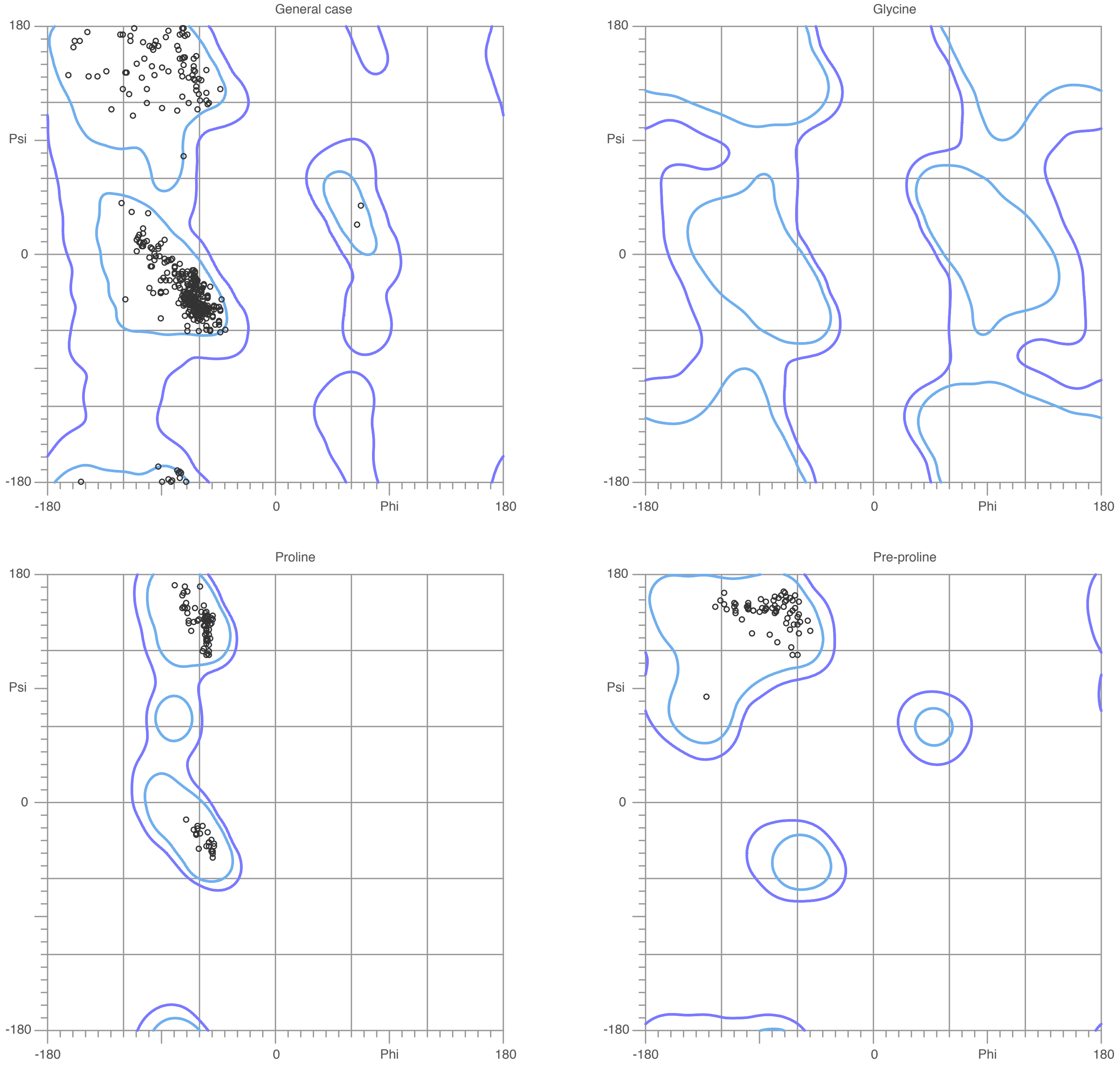

99.5% (569/572) of all residues were in favored (98%) regions.
100.0% (572/572) of all residues were in allowed (>99.8%) regions.

There were no outliers.

http://kinemage.biochem.duke.edu

Lovell, Davis, et al. Proteins 50:437 (2003)

MolProbity Ramachandran analysis

GHSRg_top1000pro.pdb, model 1

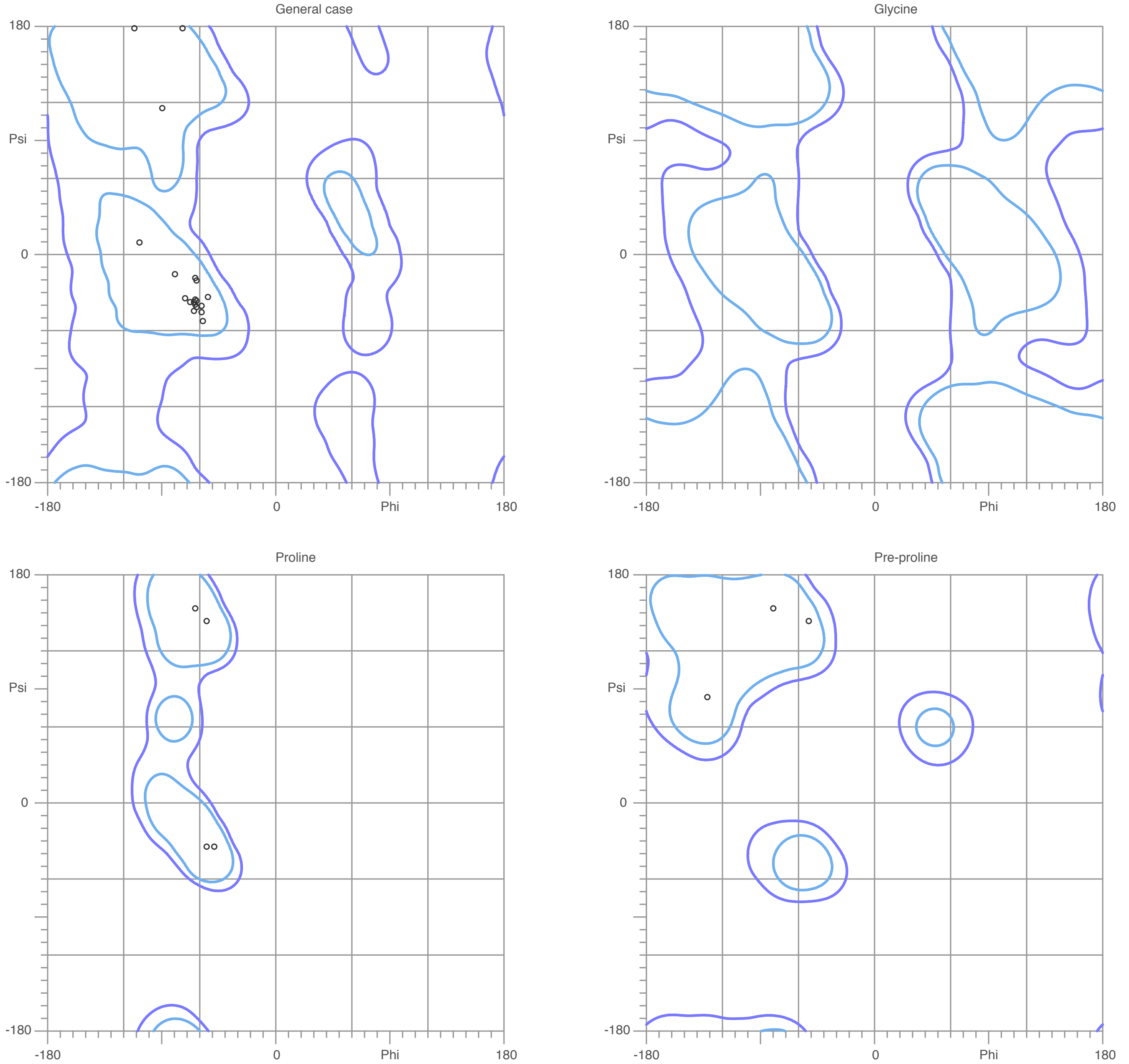

100.0% (26/26) of all residues were in favored (98%) regions.
100.0% (26/26) of all residues were in allowed (>99.8%) regions.

There were no outliers.

http://kinemage.biochem.duke.edu

Lovell, Davis, et al. Proteins 50:437 (2003)

MolProbity Ramachandran analysis

GHSRg_top1000pro.pdb, model 2

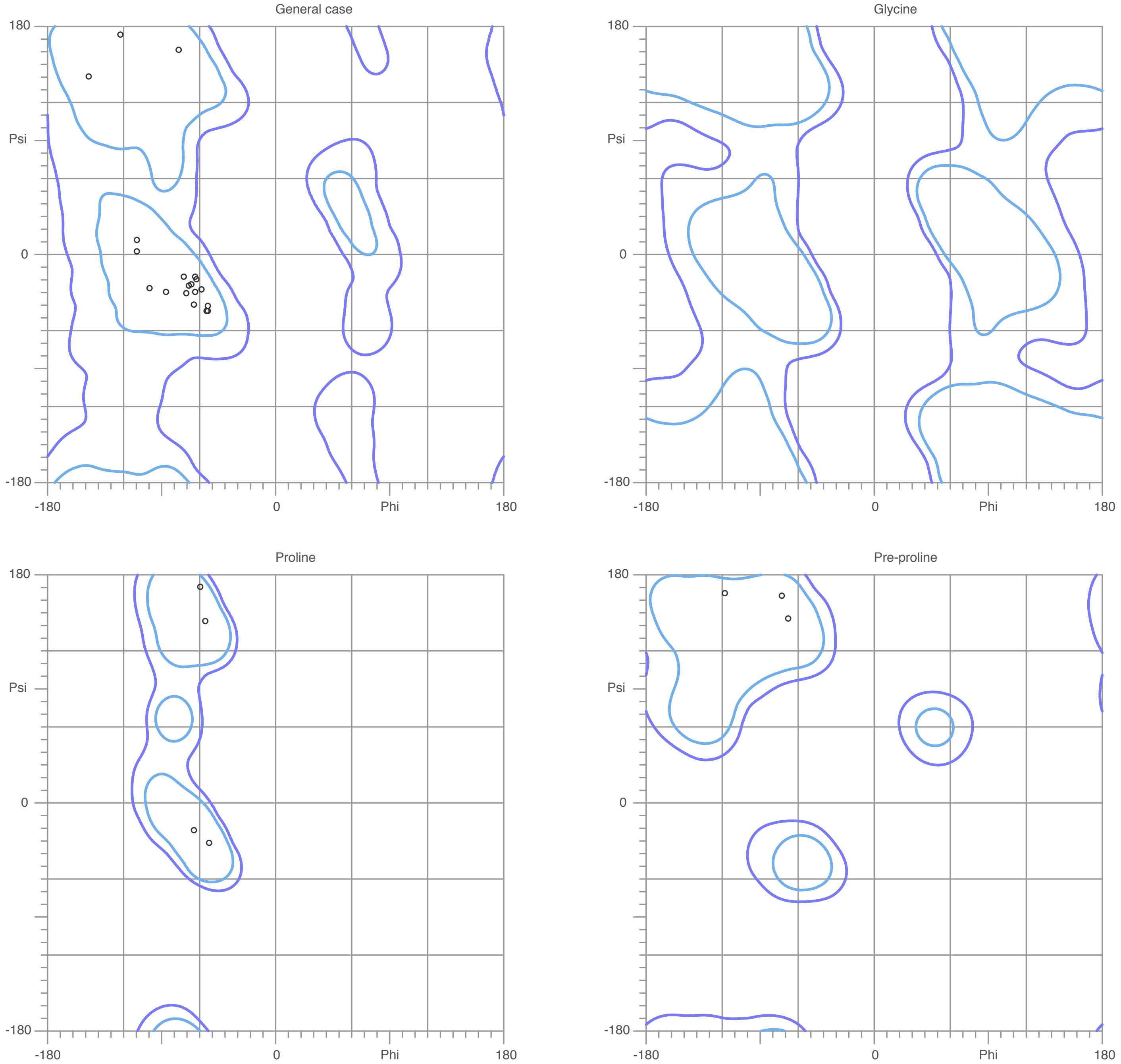

100.0% (26/26) of all residues were in favored (98%) regions.
100.0% (26/26) of all residues were in allowed (>99.8%) regions.

There were no outliers.

http://kinemage.biochem.duke.edu

Lovell, Davis, et al. Proteins 50:437 (2003)

MolProbity Ramachandran analysis

GHSRg_top1000pro.pdb, model 3

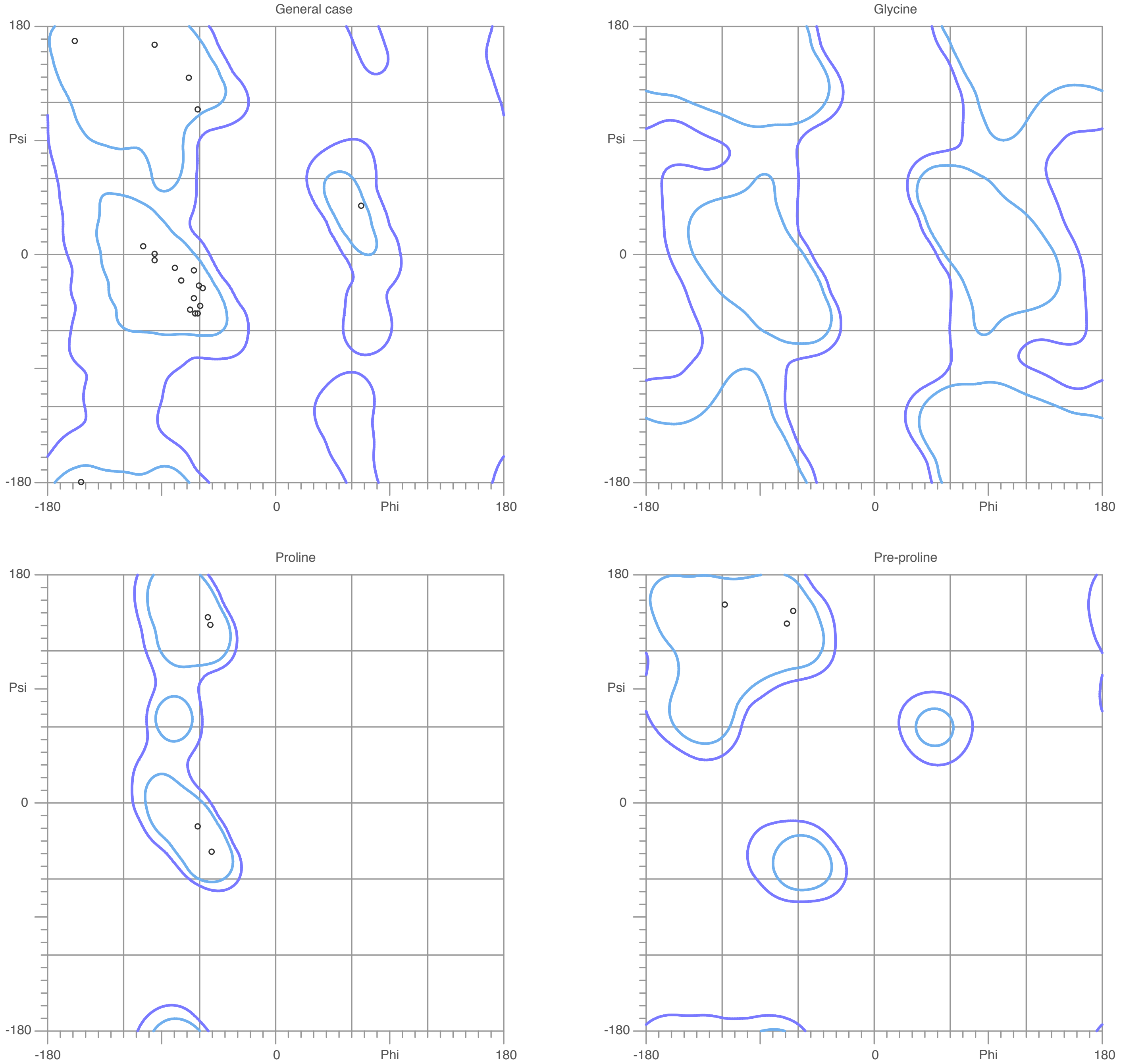

100.0% (26/26) of all residues were in favored (98%) regions.
100.0% (26/26) of all residues were in allowed (>99.8%) regions.

There were no outliers.

http://kinemage.biochem.duke.edu

Lovell, Davis, et al. Proteins 50:437 (2003)

MolProbity Ramachandran analysis

GHSRg_top1000pro.pdb, model 4

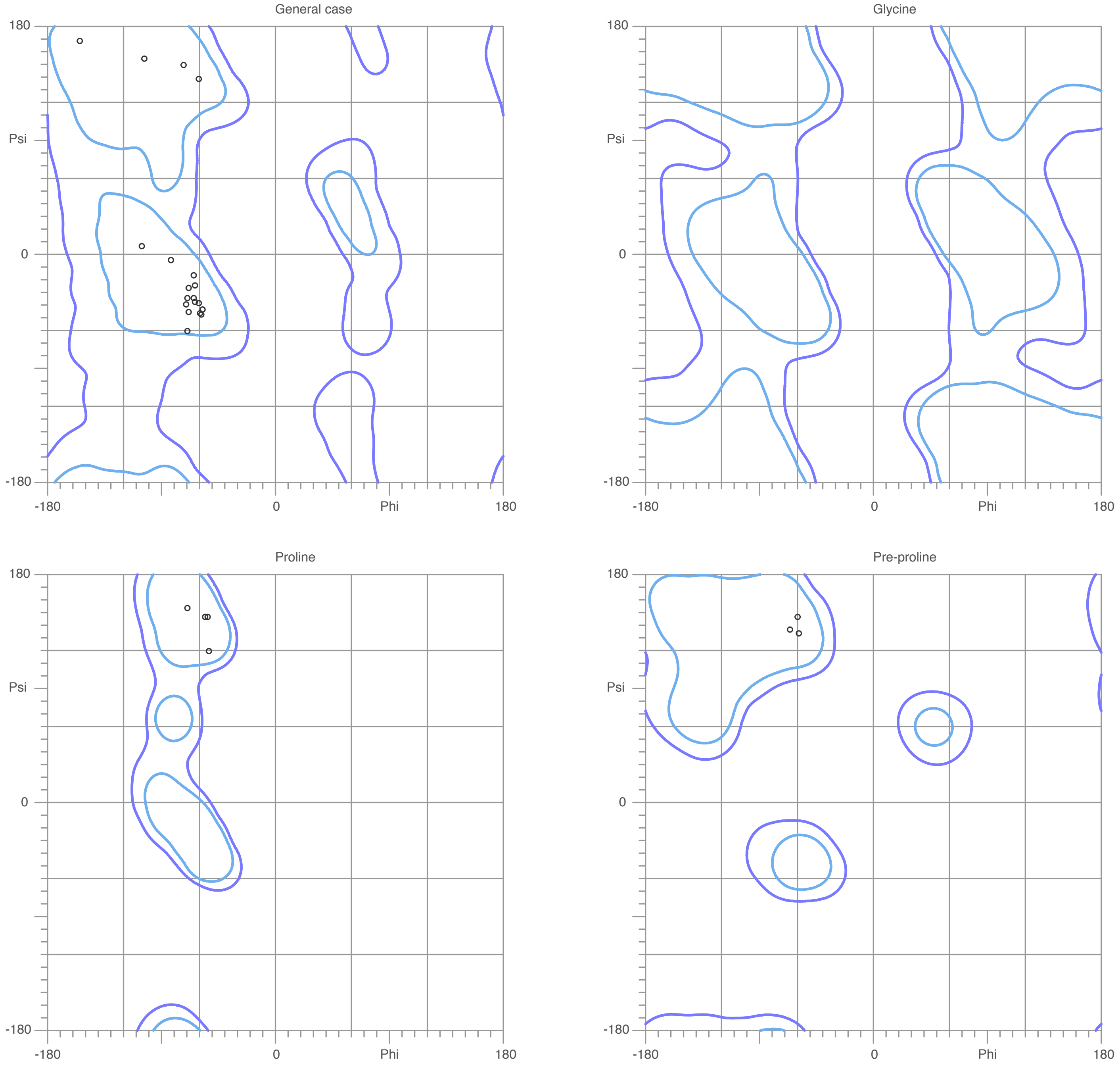

100.0% (26/26) of all residues were in favored (98%) regions.
100.0% (26/26) of all residues were in allowed (>99.8%) regions.

There were no outliers.

MolProbity Ramachandran analysis

GHSRg_top1000pro.pdb, model 5

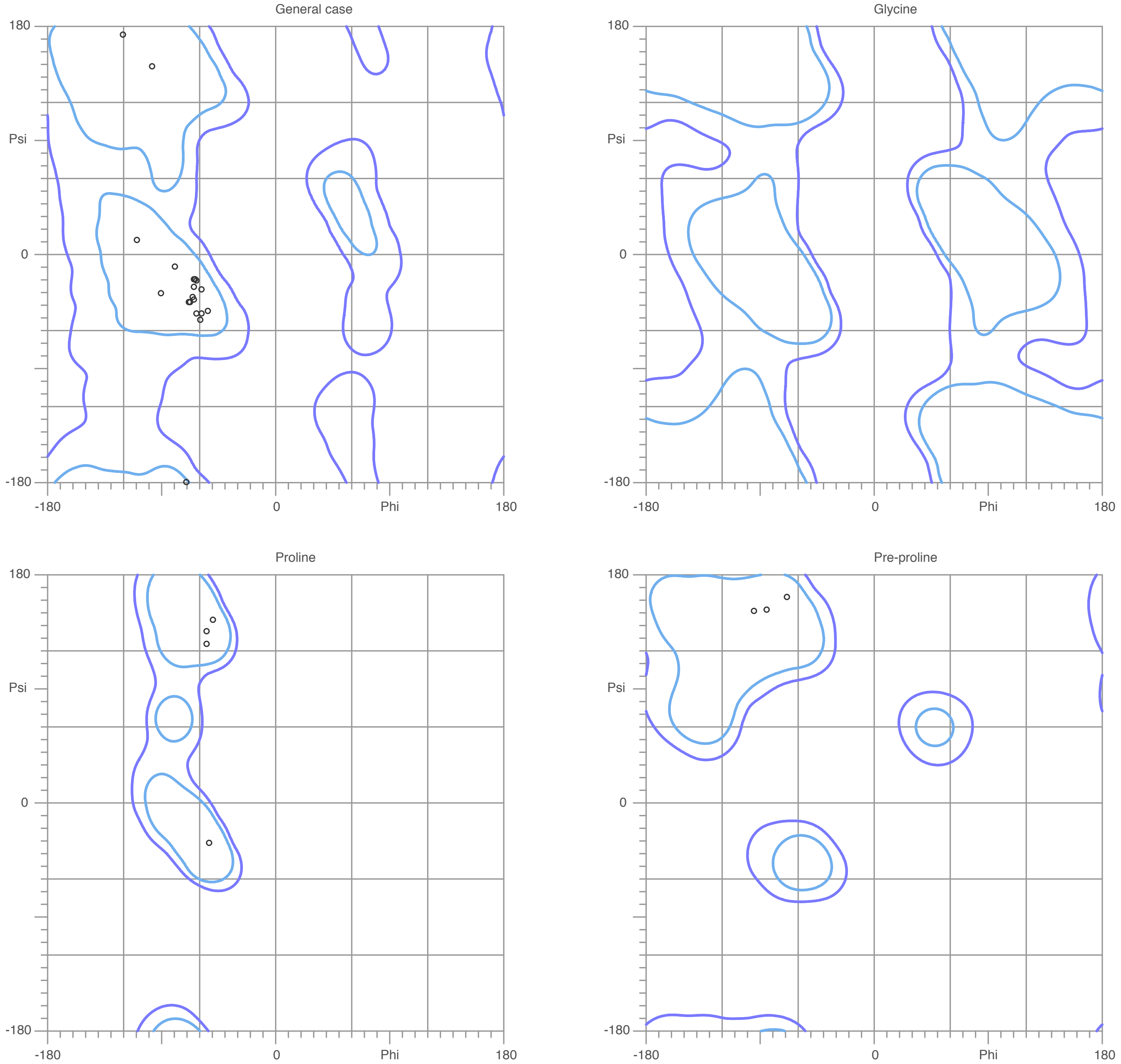

100.0% (26/26) of all residues were in favored (98%) regions.
100.0% (26/26) of all residues were in allowed (>99.8%) regions.

There were no outliers.

http://kinemage.biochem.duke.edu

Lovell, Davis, et al. Proteins 50:437 (2003)

MolProbity Ramachandran analysis

GHSRg_top1000pro.pdb, model 6

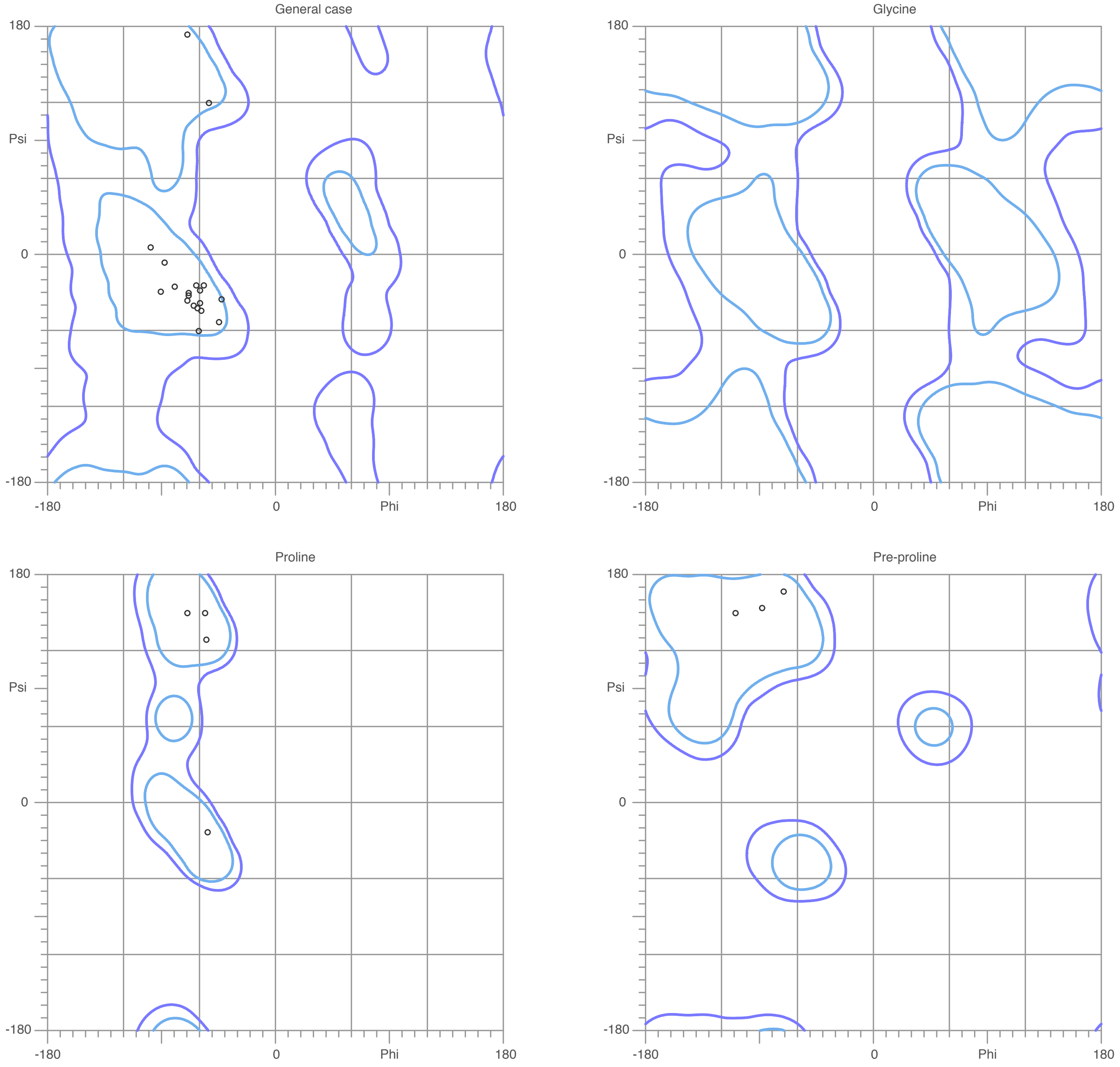

100.0% (26/26) of all residues were in favored (98%) regions.
100.0% (26/26) of all residues were in allowed (>99.8%) regions.

There were no outliers.

http://kinemage.biochem.duke.edu

Lovell, Davis, et al. Proteins 50:437 (2003)

MolProbity Ramachandran analysis

GHSRg_top1000pro.pdb, model 7

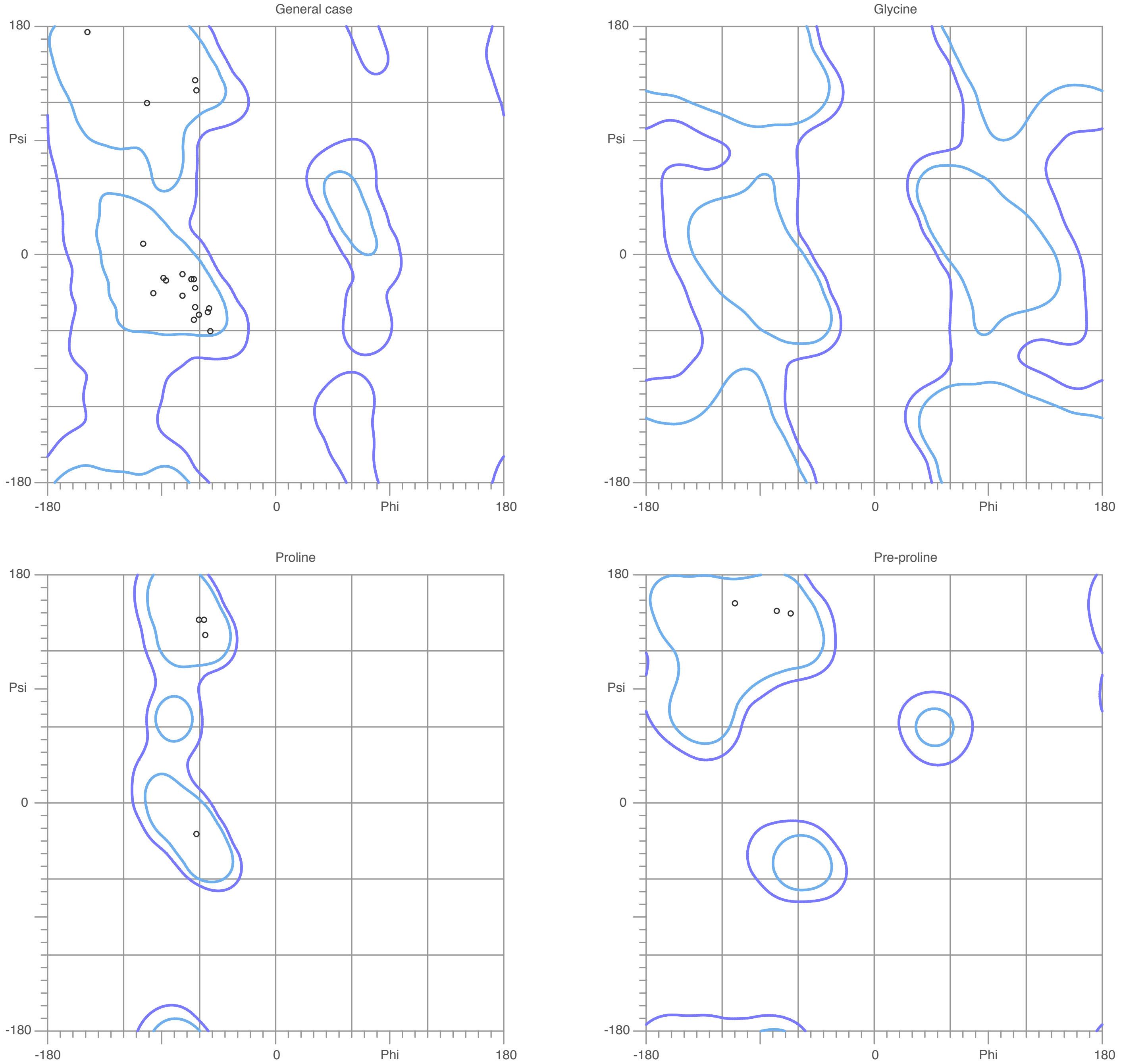

100.0% (26/26) of all residues were in favored (98%) regions.
100.0% (26/26) of all residues were in allowed (>99.8%) regions.

There were no outliers.

http://kinemage.biochem.duke.edu

Lovell, Davis, et al. Proteins 50:437 (2003)

MolProbity Ramachandran analysis

GHSRg_top1000pro.pdb, model 8

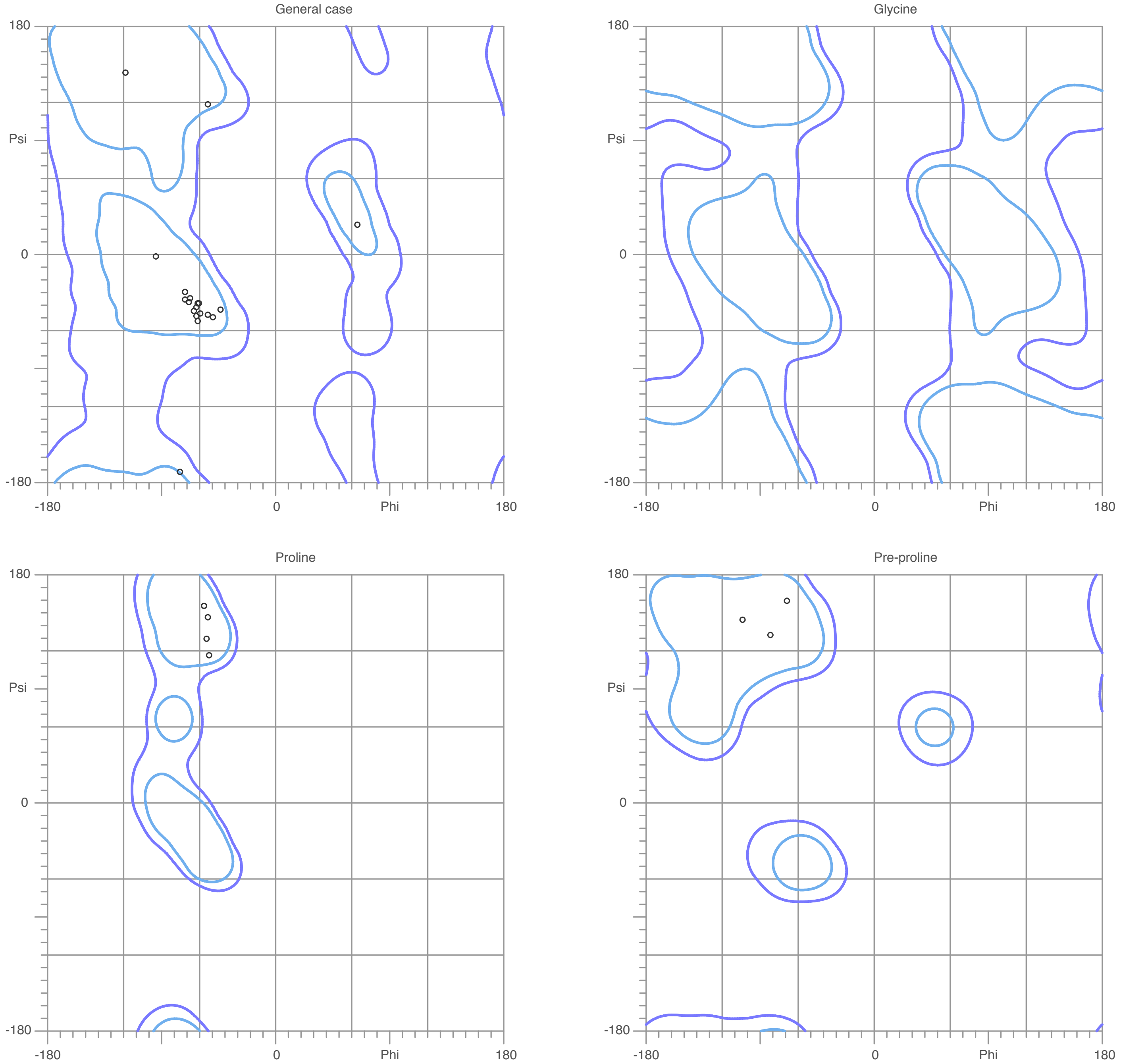

100.0% (26/26) of all residues were in favored (98%) regions.
100.0% (26/26) of all residues were in allowed (>99.8%) regions.

There were no outliers.

http://kinemage.biochem.duke.edu

Lovell, Davis, et al. Proteins 50:437 (2003)

MolProbity Ramachandran analysis

GHSRg_top1000pro.pdb, model 9

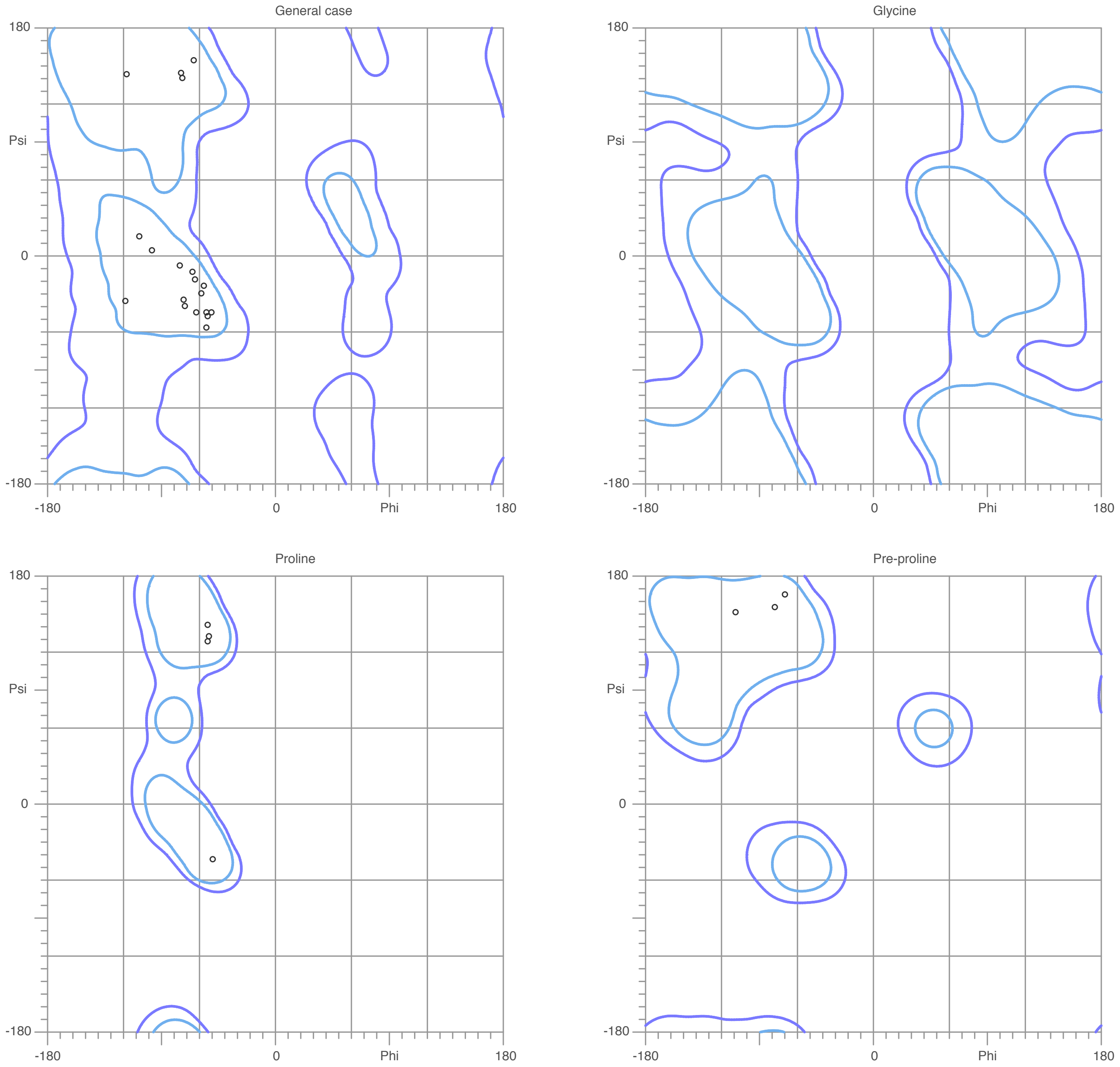

100.0% (26/26) of all residues were in favored (98%) regions.
100.0% (26/26) of all residues were in allowed (>99.8%) regions.

There were no outliers.

http://kinemage.biochem.duke.edu

Lovell, Davis, et al. Proteins 50:437 (2003)

MolProbity Ramachandran analysis

GHSRg_top1000pro.pdb, model 10

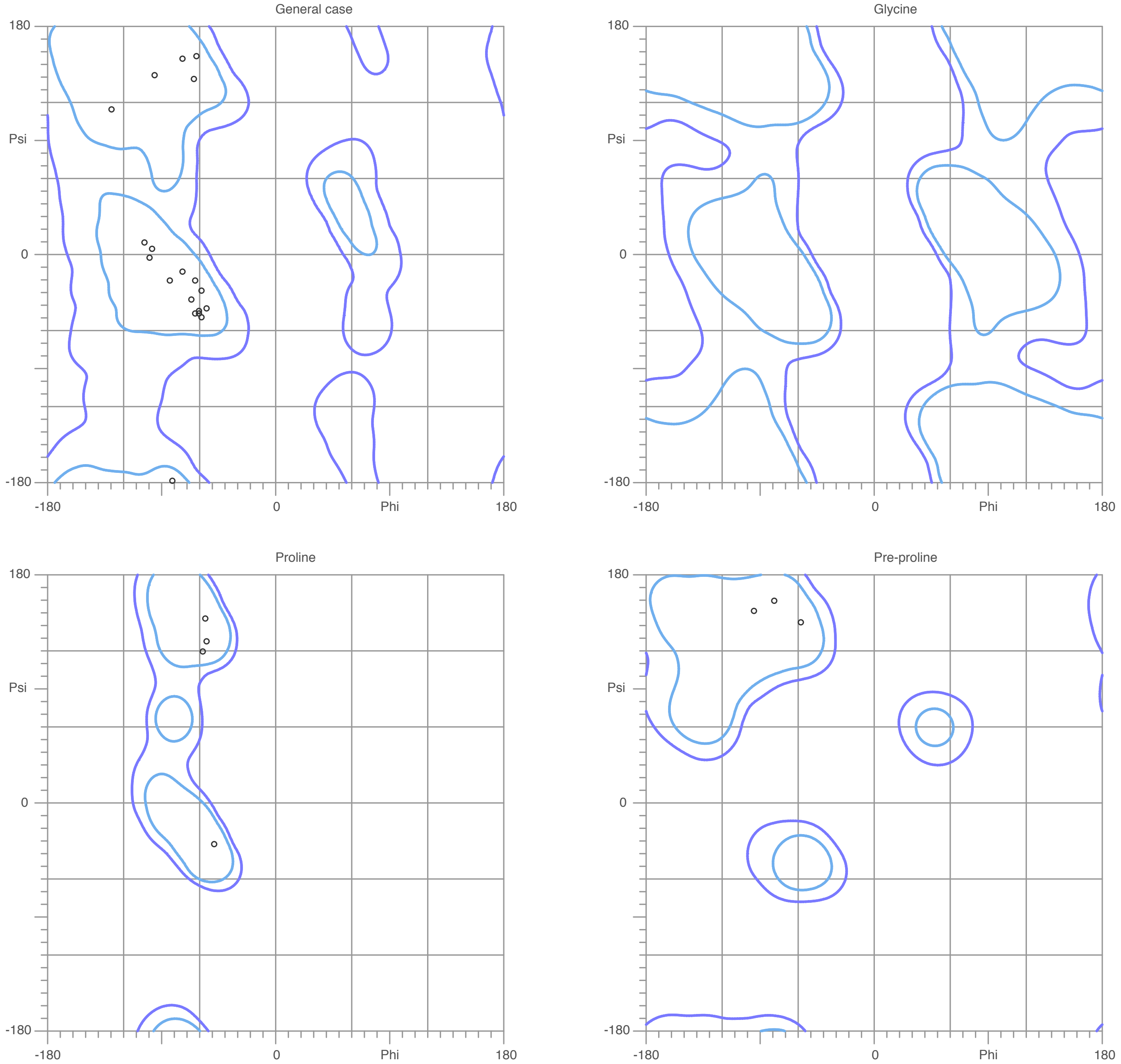

100.0% (26/26) of all residues were in favored (98%) regions.
100.0% (26/26) of all residues were in allowed (>99.8%) regions.

There were no outliers.

http://kinemage.biochem.duke.edu

Lovell, Davis, et al. Proteins 50:437 (2003)

MolProbity Ramachandran analysis

GHSRg_top1000pro.pdb, model 11

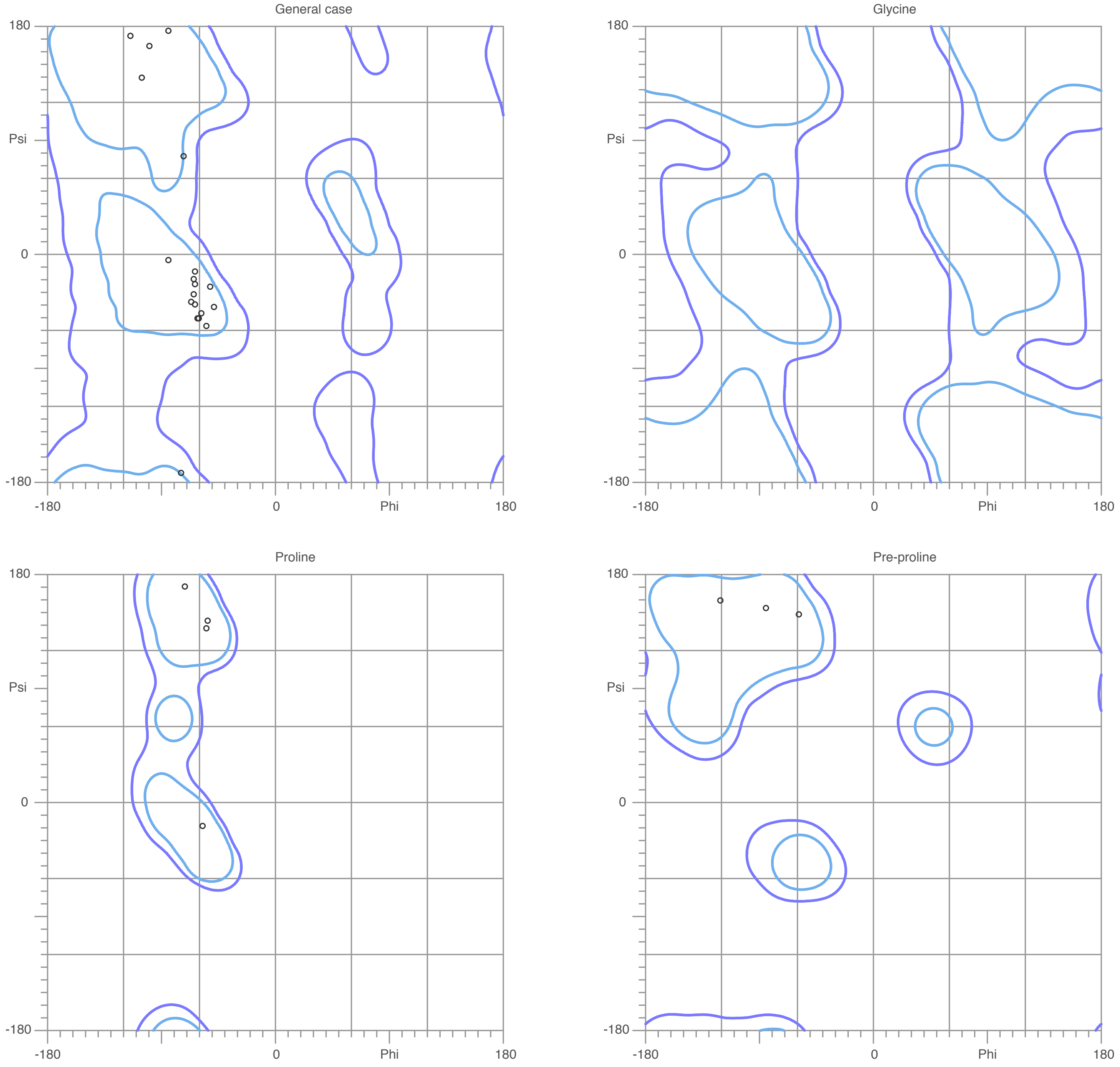

96.2% (25/26) of all residues were in favored (98%) regions.
100.0% (26/26) of all residues were in allowed (>99.8%) regions.

There were no outliers.

http://kinemage.biochem.duke.edu

Lovell, Davis, et al. Proteins 50:437 (2003)

MolProbity Ramachandran analysis

GHSRg_top1000pro.pdb, model 12

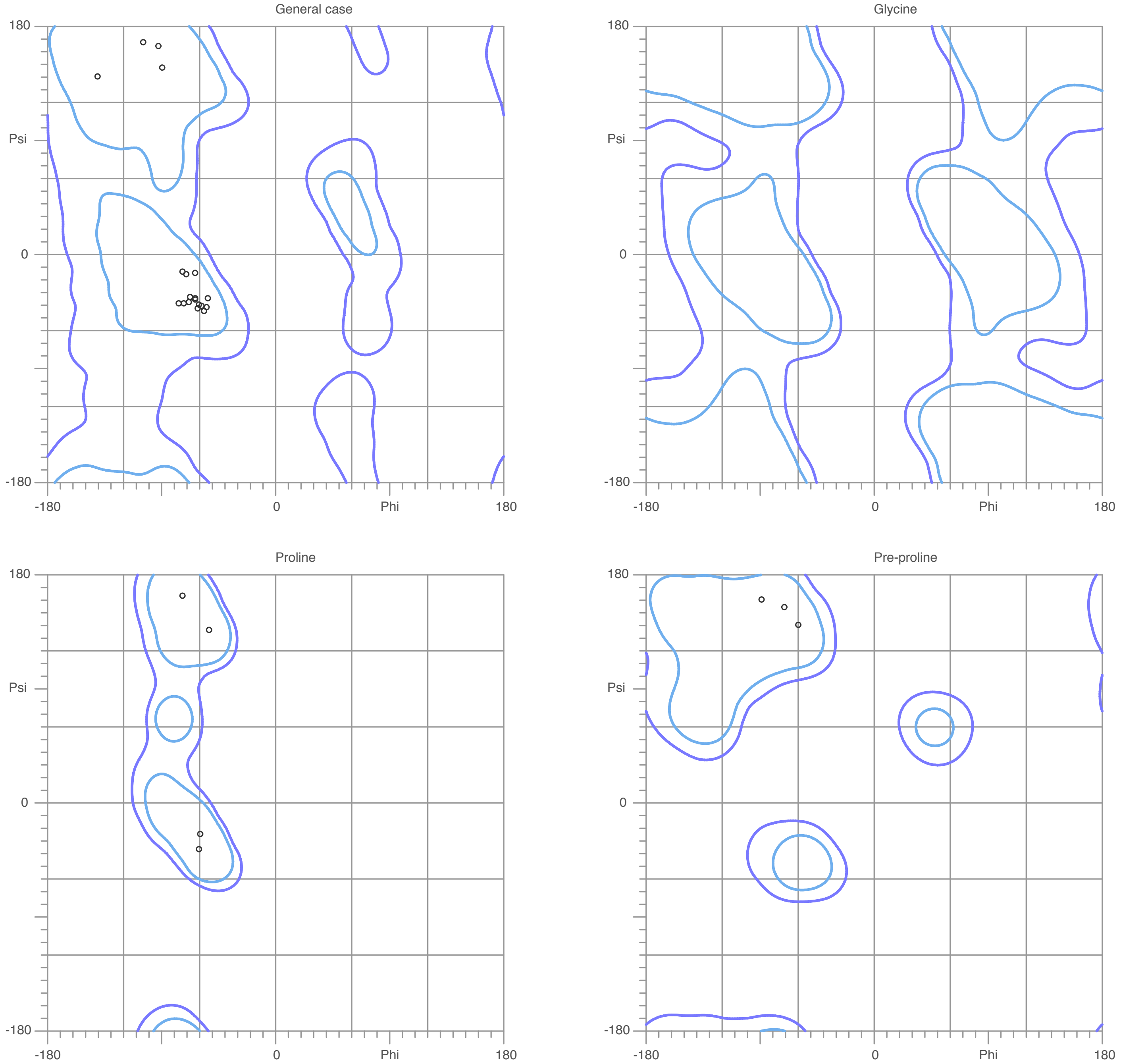

100.0% (26/26) of all residues were in favored (98%) regions.
100.0% (26/26) of all residues were in allowed (>99.8%) regions.

There were no outliers.

http://kinemage.biochem.duke.edu

Lovell, Davis, et al. Proteins 50:437 (2003)

MolProbity Ramachandran analysis

GHSRg_top1000pro.pdb, model 13

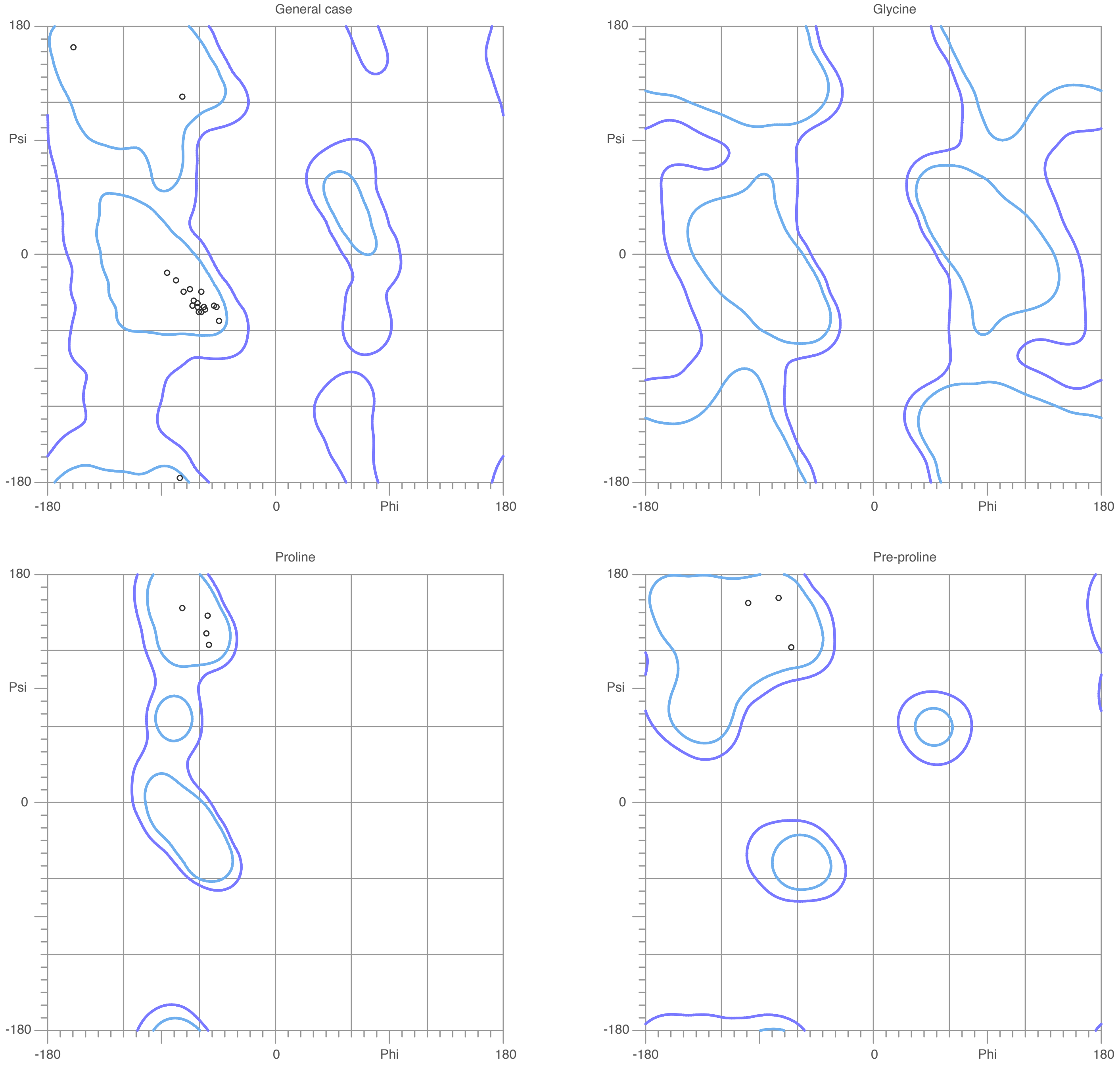

100.0% (26/26) of all residues were in favored (98%) regions.
100.0% (26/26) of all residues were in allowed (>99.8%) regions.

There were no outliers.

http://kinemage.biochem.duke.edu

Lovell, Davis, et al. Proteins 50:437 (2003)

MolProbity Ramachandran analysis

GHSRg_top1000pro.pdb, model 14

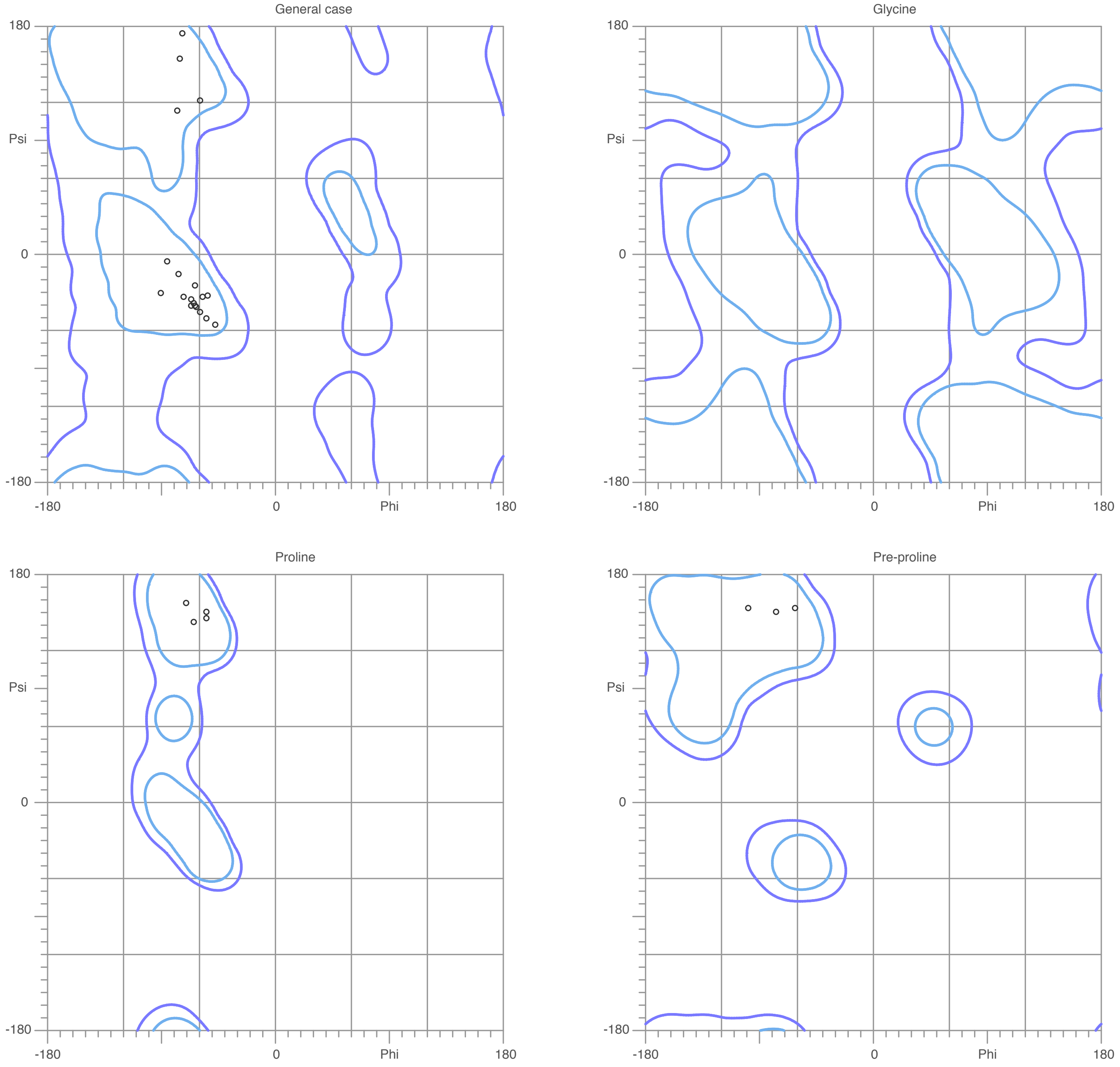

100.0% (26/26) of all residues were in favored (98%) regions.
100.0% (26/26) of all residues were in allowed (>99.8%) regions.

There were no outliers.

http://kinemage.biochem.duke.edu

Lovell, Davis, et al. Proteins 50:437 (2003)

MolProbity Ramachandran analysis

GHSRg_top1000pro.pdb, model 15

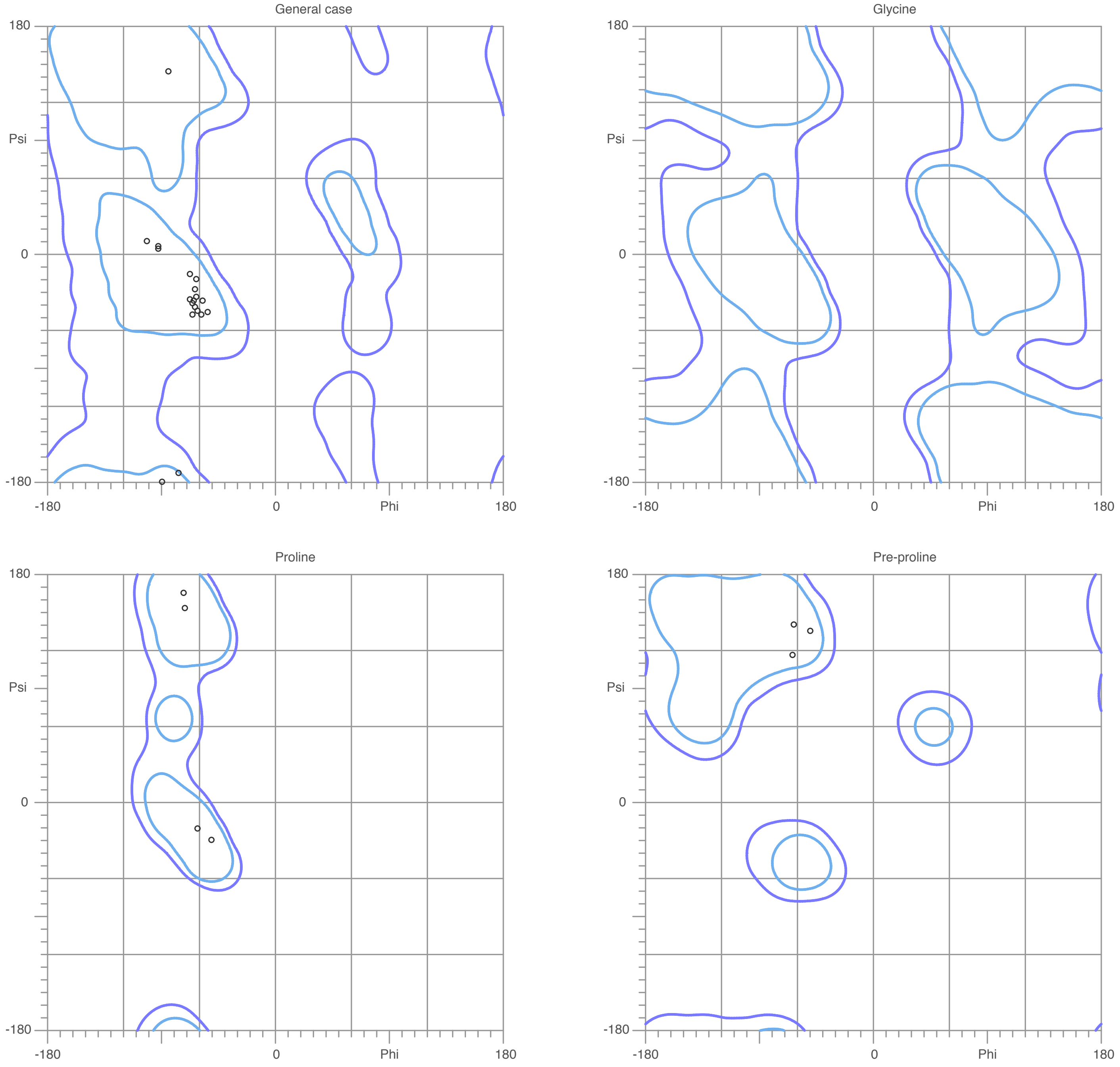

100.0% (26/26) of all residues were in favored (98%) regions.
100.0% (26/26) of all residues were in allowed (>99.8%) regions.

There were no outliers.

http://kinemage.biochem.duke.edu

Lovell, Davis, et al. Proteins 50:437 (2003)

MolProbity Ramachandran analysis

GHSRg_top1000pro.pdb, model 16

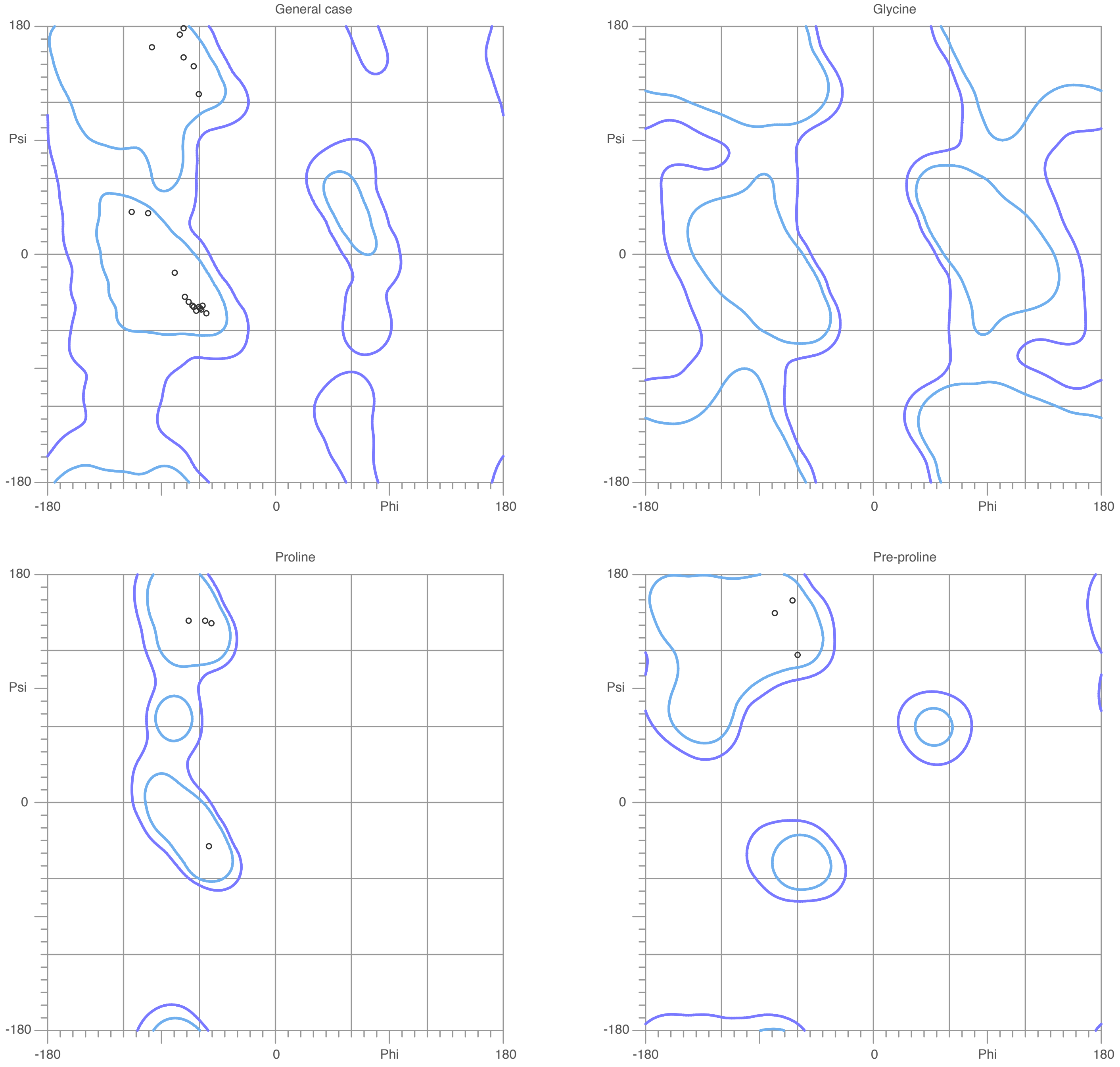

100.0% (26/26) of all residues were in favored (98%) regions.
100.0% (26/26) of all residues were in allowed (>99.8%) regions.

There were no outliers.

http://kinemage.biochem.duke.edu

Lovell, Davis, et al. Proteins 50:437 (2003)

MolProbity Ramachandran analysis

GHSRg_top1000pro.pdb, model 17

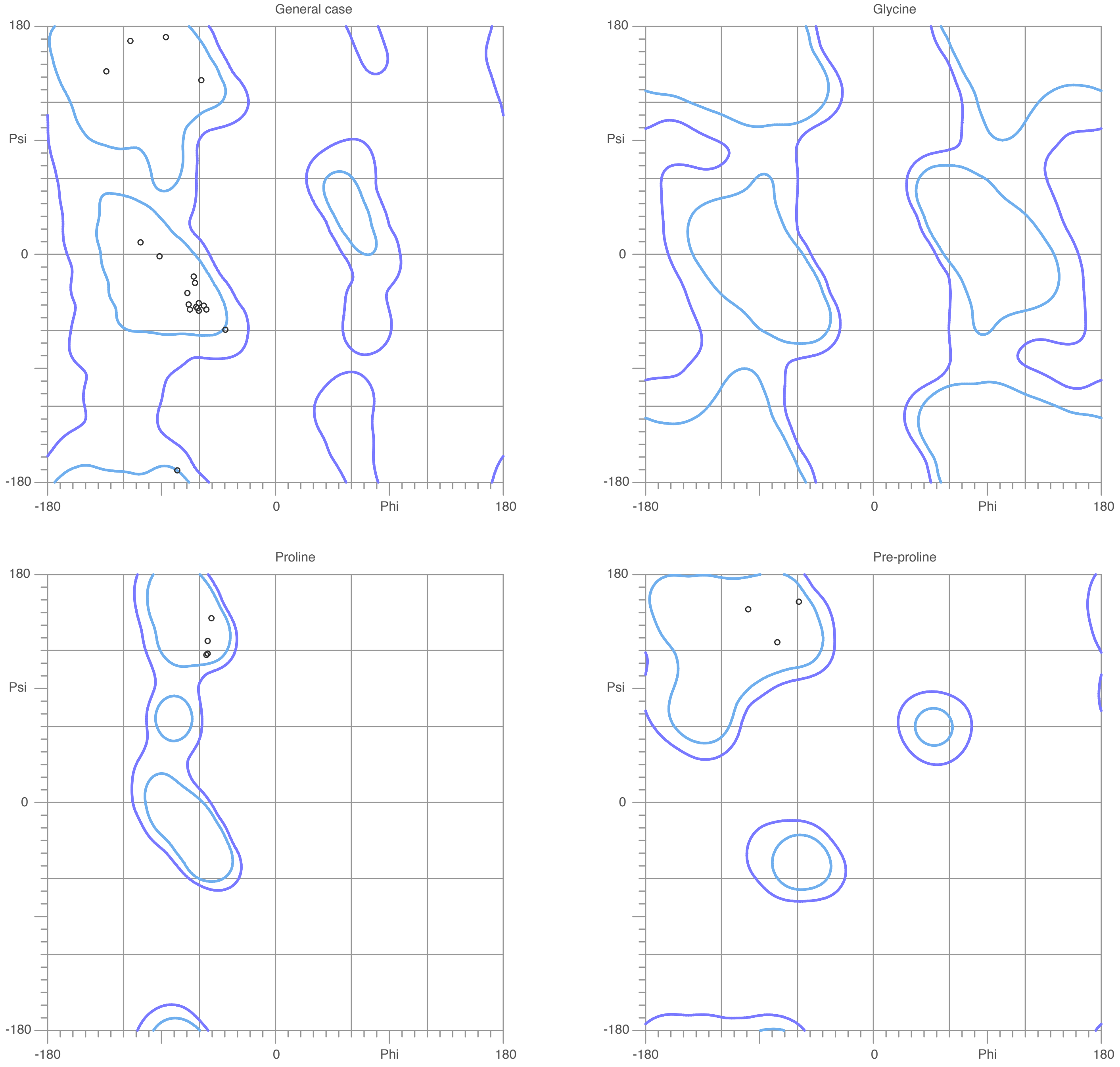

96.2% (25/26) of all residues were in favored (98%) regions.
100.0% (26/26) of all residues were in allowed (>99.8%) regions.

There were no outliers.

http://kinemage.biochem.duke.edu

Lovell, Davis, et al. Proteins 50:437 (2003)

MolProbity Ramachandran analysis

GHSRg_top1000pro.pdb, model 18

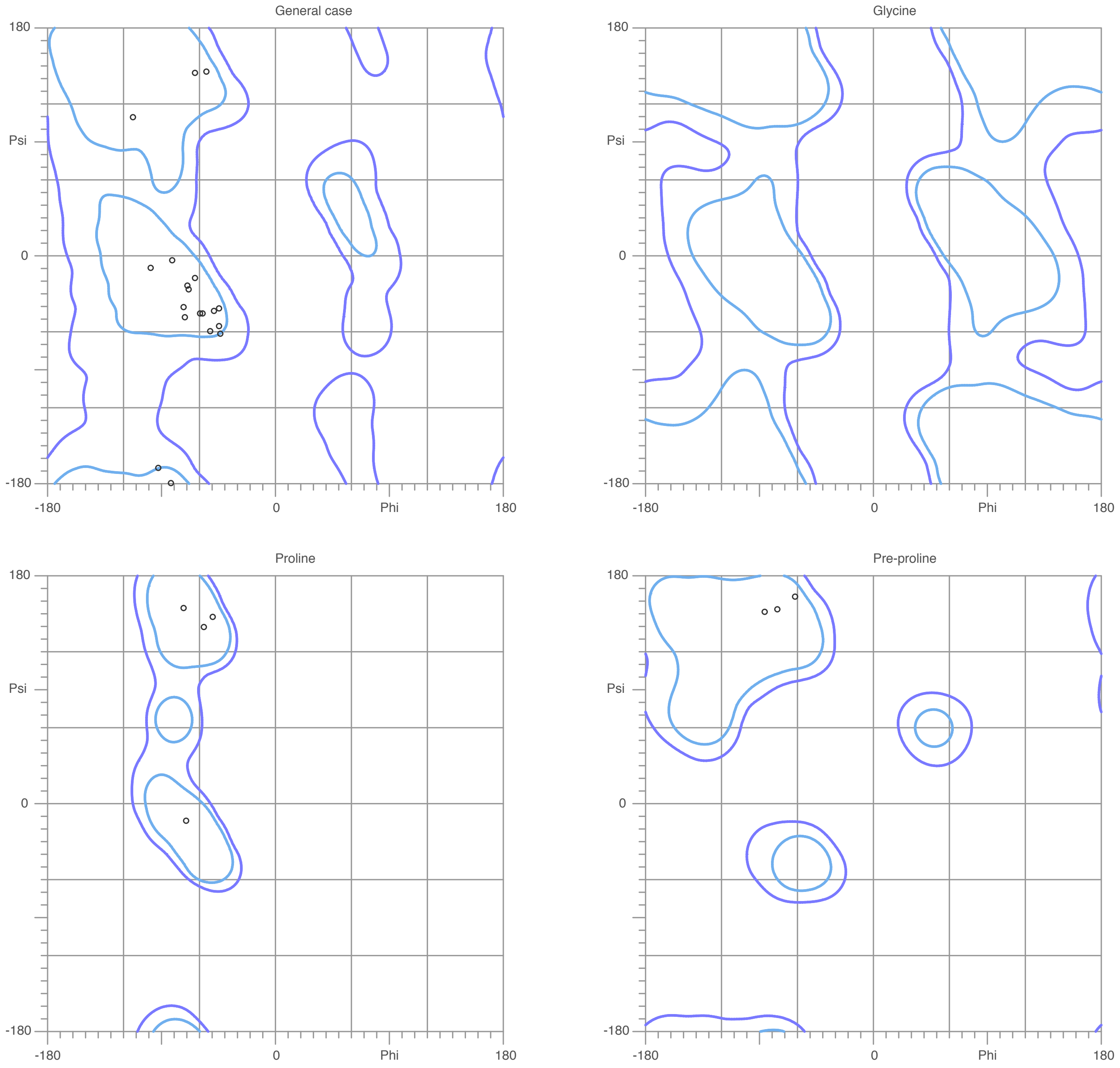

96.2% (25/26) of all residues were in favored (98%) regions.
100.0% (26/26) of all residues were in allowed (>99.8%) regions.

There were no outliers.

http://kinemage.biochem.duke.edu

Lovell, Davis, et al. Proteins 50:437 (2003)

MolProbity Ramachandran analysis

GHSRg_top1000pro.pdb, model 19

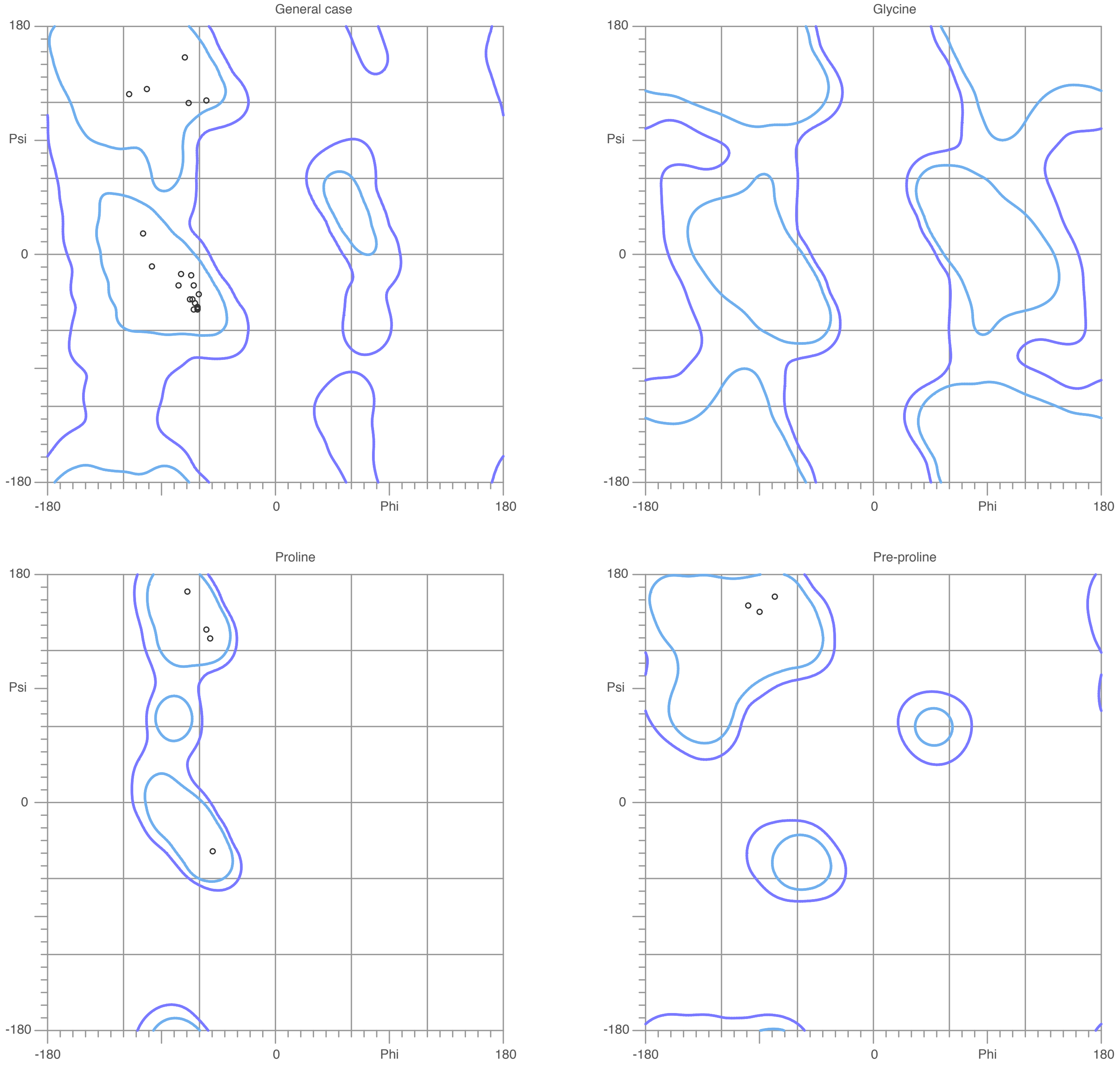

100.0% (26/26) of all residues were in favored (98%) regions.
100.0% (26/26) of all residues were in allowed (>99.8%) regions.

There were no outliers.

http://kinemage.biochem.duke.edu

Lovell, Davis, et al. Proteins 50:437 (2003)

MolProbity Ramachandran analysis

GHSRg_top1000pro.pdb, model 20

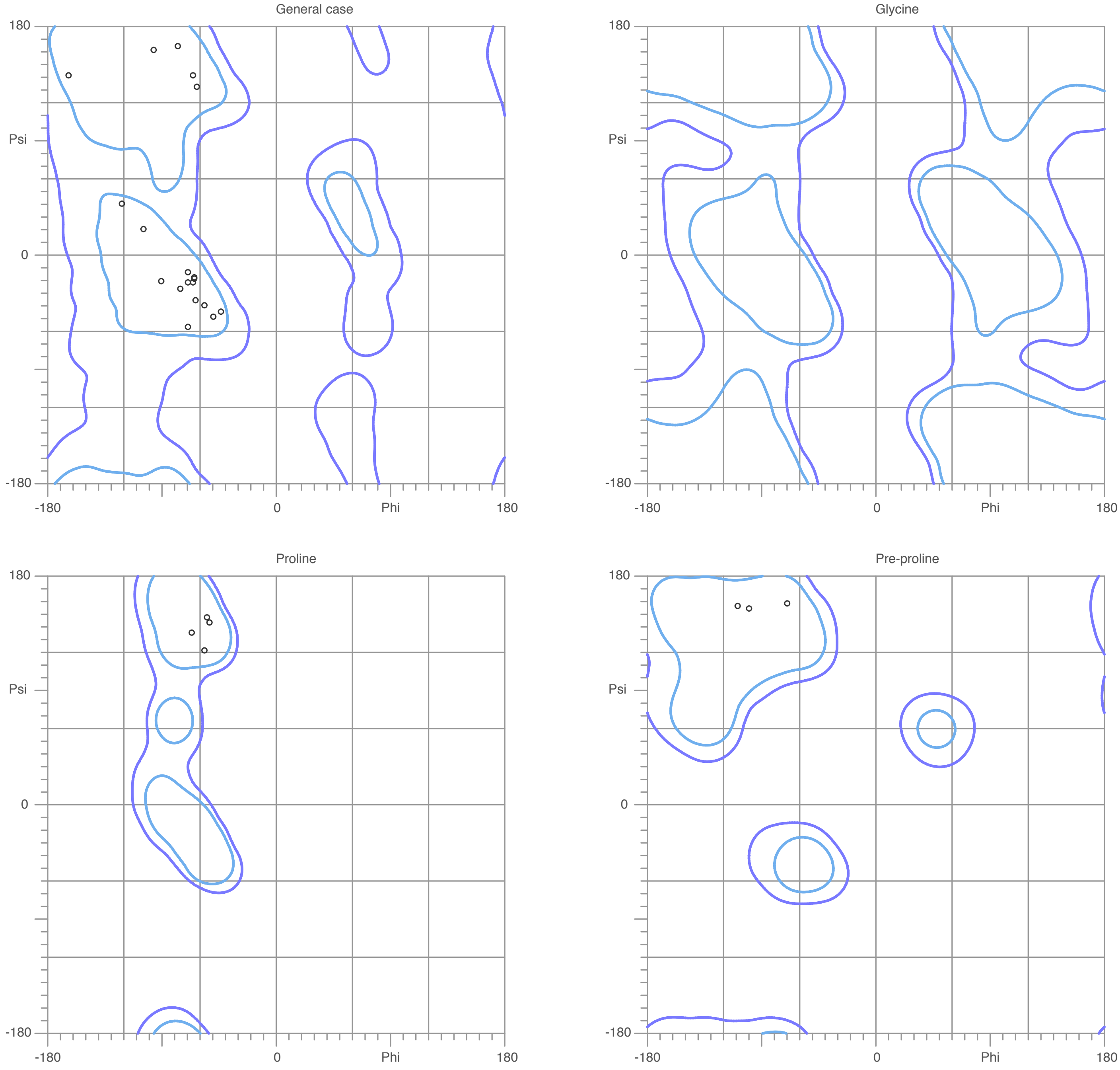

100.0% (26/26) of all residues were in favored (98%) regions.
100.0% (26/26) of all residues were in allowed (>99.8%) regions.

There were no outliers.

http://kinemage.biochem.duke.edu

Lovell, Davis, et al. Proteins 50:437 (2003)

MolProbity Ramachandran analysis

GHSRg_top1000pro.pdb, model 21

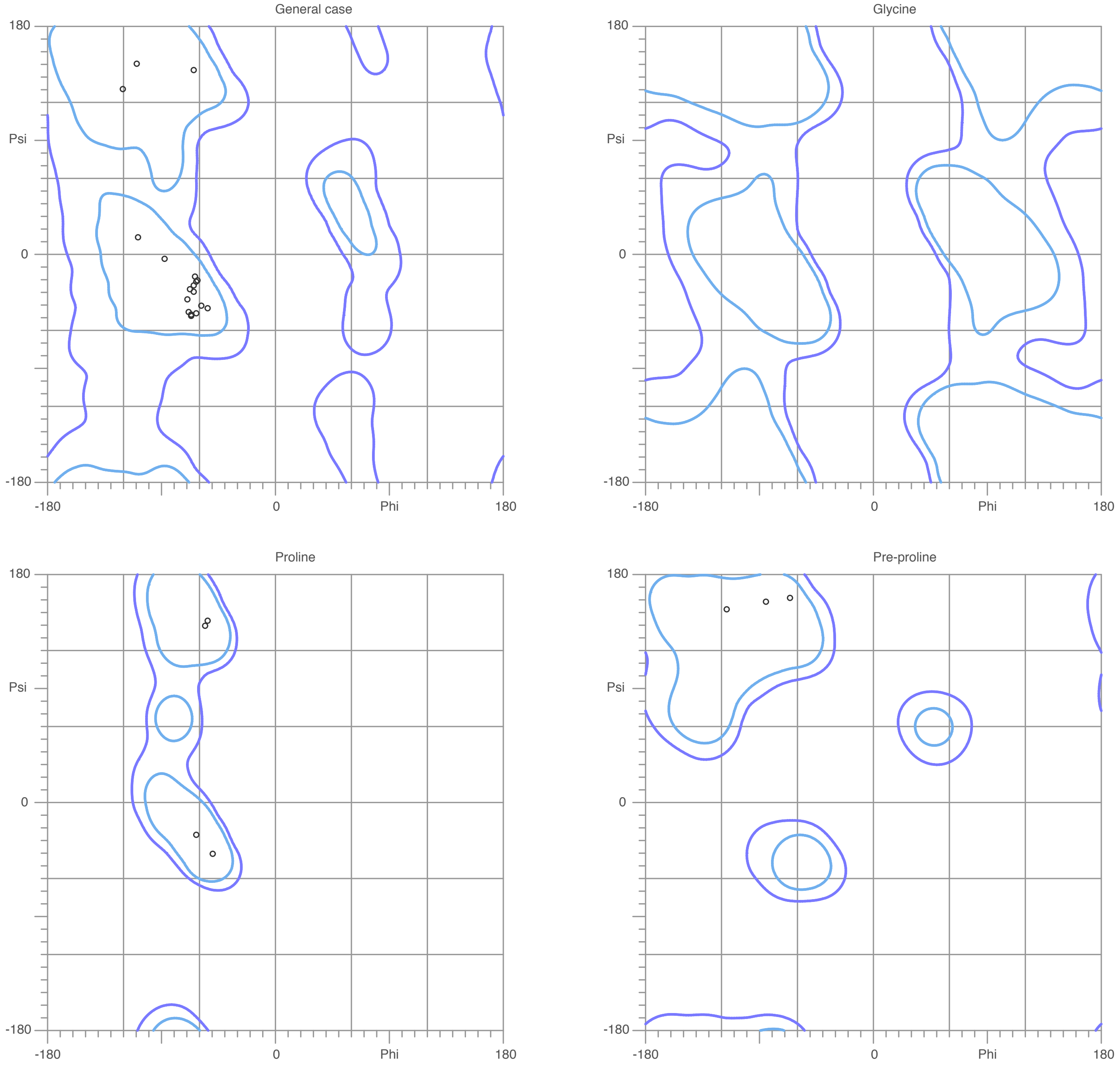

100.0% (26/26) of all residues were in favored (98%) regions.
100.0% (26/26) of all residues were in allowed (>99.8%) regions.

There were no outliers.

http://kinemage.biochem.duke.edu

Lovell, Davis, et al. Proteins 50:437 (2003)

MolProbity Ramachandran analysis

GHSRg_top1000pro.pdb, model 22

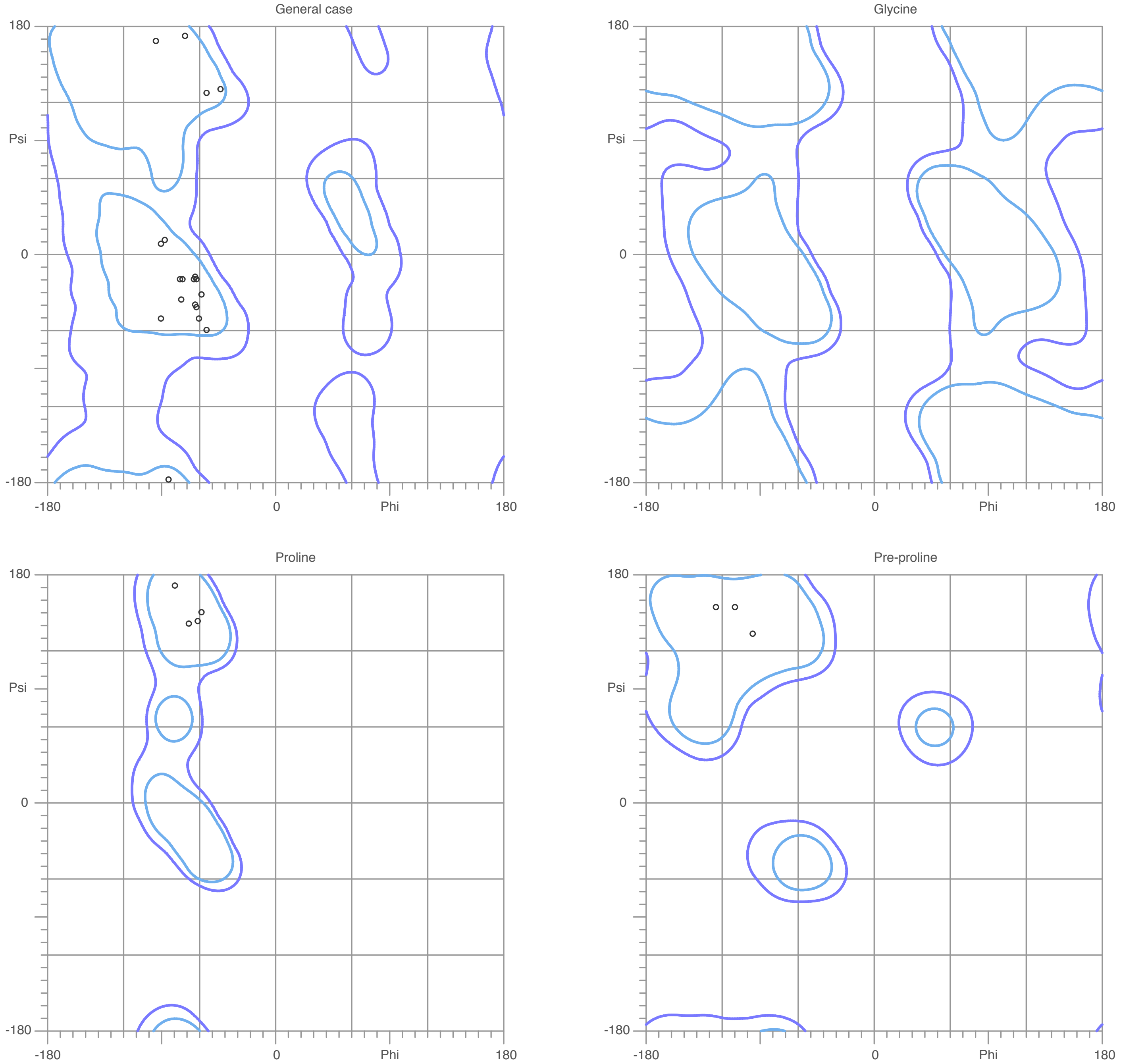

100.0% (26/26) of all residues were in favored (98%) regions.
100.0% (26/26) of all residues were in allowed (>99.8%) regions.

There were no outliers.

http://kinemage.biochem.duke.edu

Lovell, Davis, et al. Proteins 50:437 (2003)
