# Supplementary material for: Integrating Solid-State NMR and Computational Modeling to Investigate the Structure and Dynamics of Membrane-Associated Ghrelin
Source: PLoS One. 2015 Mar 24;10(3):e0122444. doi: 10.1371/journal.pone.0122444 (PMC4372444; doi:10.1371/journal.pone.0122444)
Supplement: S2 File — (TGZ) [file pone.0122444.s008.tgz › ghrelin/folding_analysis/PSVS_analysis/prosa_lnx.html]

Protein Structure Quality Analysis Result


the pdf file for ProsaII Score

the postscript file for ProsaII Score

JPEG image for ProsaII Score

Table of ProsaII scores for ordered residues across all models

  
