# Supplementary material for: Integrating Solid-State NMR and Computational Modeling to Investigate the Structure and Dynamics of Membrane-Associated Ghrelin
Source: PLoS One. 2015 Mar 24;10(3):e0122444. doi: 10.1371/journal.pone.0122444 (PMC4372444; doi:10.1371/journal.pone.0122444)
Supplement: S2 File — (TGZ) [file pone.0122444.s008.tgz › ghrelin/folding_analysis/PSVS_analysis/verify3d_lnx.html]

Protein Structure Quality Analysis Result


the pdf file for Verify3D Score

the postscript file for Verify3D Score

JPEG image for Verify3D Score

Table of Verify3D scores for ordered residues across all models

  
