# Supplementary material for: Integrating Solid-State NMR and Computational Modeling to Investigate the Structure and Dynamics of Membrane-Associated Ghrelin
Source: PLoS One. 2015 Mar 24;10(3):e0122444. doi: 10.1371/journal.pone.0122444 (PMC4372444; doi:10.1371/journal.pone.0122444)
Supplement: S2 File — (TGZ) [file pone.0122444.s008.tgz › ghrelin/folding_analysis/PSVS_analysis/rama_plot_lnx.html]

Protein Structure Quality Analysis Result


Text summary of Ramachandran Plot

the pdf file for all model Ramachandran Plot

the postscript file for all model Ramachandran Plot

JPEG image for all model Ramachandran Plot

  
