# Supplementary material for: Integrating Solid-State NMR and Computational Modeling to Investigate the Structure and Dynamics of Membrane-Associated Ghrelin
Source: PLoS One. 2015 Mar 24;10(3):e0122444. doi: 10.1371/journal.pone.0122444 (PMC4372444; doi:10.1371/journal.pone.0122444)
Supplement: S2 File — (TGZ) [file pone.0122444.s008.tgz › ghrelin/folding_analysis/PSVS_analysis/fsvr/output_NAME.html]

Structure Quality Analysis for NAME


# Structure Quality Analysis for NAME

Analyses performed for all residues.  
Procheck analysis,RMSD calculation and structure superimposition are based on: all residues

|  |  |  |  |  |  |  |  |  |  |  |  |  |  |  |  |  |  |  |  |  |  |  |  |  |  |  |
| --- | --- | --- | --- | --- | --- | --- | --- | --- | --- | --- | --- | --- | --- | --- | --- | --- | --- | --- | --- | --- | --- | --- | --- | --- | --- | --- |
| |  |  | | --- | --- | | NESG ID: | NAME | | PDB ID: |  | | Deposition date: |  | | Common Name: |  | | Class: |  | | Length (a.a.): | 28 | | Organism: |  | | SwissProt / TrEMBL ID: |  | | # models: | 22 | | Oligomerization: | monomer | | Molecular weight: | 3245 | | |  | | --- | |  | | | | |

Secondary Structure Elements:   
alpha helices: 7A-17A  
beta strands:

FIDs deposited in the BMRB? no

|  |  |  |  |
| --- | --- | --- | --- |
| RMSD | *All residues* | *Ordered residues2* | *Selected residues3* |
| *All backbone atoms* | 4.0 Å | 1.3 Å | 4.0 Å |
| *All heavy atoms* | 5.3 Å | 2.5 Å | 5.3 Å |

Ramachandran Plot Summary for selected residues3 from Procheck

|  |  |  |  |
| --- | --- | --- | --- |
| *Most favoured regions* | *Additionally allowed regions* | *Generously allowed regions* | *Disallowed regions* |
| 95.2% | 4.8% | 0.0% | 0.0% |

Ramachandran Plot Summary for selected residues3 from Richardson Lab's Molprobity

|  |  |  |  |
| --- | --- | --- | --- |
| *Most favoured regions* | *Allowed regions* | *Disallowed regions* | View plot View model summary |
| 99.5% | 0.5% | 0% |

**Global quality scores**

|  |  |  |  |  |  |
| --- | --- | --- | --- | --- | --- |
| Program | *Verify3D* | *ProsaII (-ve)* | *Procheck (phi-psi)3* | *Procheck (all)3* | *MolProbity Clashscore* |
| *-Raw score* | 0.24 | 0.60 | 0.13 | 0.29 | 3.01 |
| *Z-score1* | -3.53 | -0.21 | 0.83 | 1.71 | 1.01 |

Close Contacts and Deviations from Ideal Geometry (from PDB validation software)

|  |  |
| --- | --- |
| Number of close contacts (within 1.6 Å for H atoms, 2.2 Å for heavy atoms): | 0 |
| RMS deviation for bond angles: | 0.7 ° |
| RMS deviation for bond lengths: | 0.017 Å |

1 With respect to mean and standard deviation for a set of 252 X-ray structures < 500 residues, of resolution <= 1.80 Å, R-factor <= 0.25 and R-free <= 0.28; a positive value indicates a 'better' score
  
2Order residues: HASH(0xd7f3c0)
  
3Selected residues: all

**Residue Plot of Ramachandran anlysis(based on data from Richardson Lab's Molprobity)**

***References:***  
1. Luthy R, Bowie J U and Eisenberg D, "Assessment of protein models with three-dimensional profiles", Nature 356 (1992): 83-85  
2. Bowie J U, Luthy R and Eisenberg D, "A Method to Identify Protein Sequences that Fold into a Known Three-Dimensional Structure", Science 253 (1991): 164-169  
3. Sippl M J, "Recognition of Errors in Three-Dimensional Structures of Proteins", Proteins 17 (1993): 355-362  
4. Sippl M J, "Calculation of Conformation Ensembles from Potentials of Mean Force", J Mol Biol 213 (1990): 859-883  
5. Laskowski R Ai et al, "AQUA and PROCHECK\_NMR: Programs for checking the quality of proteins structures solved by NMR", J Biomolec NMR 8 (1996): 477-486  
6. Laskowski R A et al "PROCHECK: a program to check the stereochemical quality of protein structures" J Appl Cryst, 26 (1993): 283-291  
7. Word J M et al, "Exploring steric constrains on protein mutations using MAGE / PROBE", Prot Sci 9 (2000): 2251-2259  
8. Word J M et al, "Asparagine and Glutamine: Using Hydrogen Atom Contacts in the Choice of Side-chain Amide Orientation", J Mol Biol 285 (1999): 1735-1747  
9. Word J M et al, "Visualizing and Quantifying Molecular Goodness-of-Fit: Small-probe Contact Dots with Explicit Hydrogens", J Mol Biol 285 (1999): 1711-1733  
10. Tejero R and Montelione G T, "PDBStat", unpublished  
11. Luthy R, McLachlan A D and Eisenberg D, "Secondary Structure-Based Profiles: Use of Structure-Conserving Scoring Tables in Searching Protein Sequence Databases for Structural Similarities", Proteins 10 (1991): 229-239  
12. Richardson D C, Richardson J S, "The kinemage: a tool for scientific communication", Prot Sci 1(1) (1992): 3-9  
13. Koradi, R, et al, "MOLMOL: a program for display and analysis of macromolecular structures ", J Mol Graphics 14 (1996): 51-55.  
14. G�ntert, P, Mumenthaler, C & W�thrich, K "Torsion angle dynamics for NMR structure calculation with the new program DYANA", J. Mol. Biol 273 (1997): 283-298  
15. Lovell S C et al, "Structure validation by Calpha geometry: phi,psi and Cbeta deviation" Proteins (2003) 50: 437-450  
16. Kabsch W, Sander C, "Dictionary of protein secondary structure: pattern recognition of hydrogen-bonded and geometrical features", Biopolymers (1983) 22: 2577-2637  
17. Bagaria, A., Jaravine, V., Huang, Y.J., Montelione, G.T., and Guntert, P. "Protein structure validation by generalized linear model root-mean-square deviation prediction". Protein Sci 21(2012), 229-238.
