# Supplementary material for: Integrating Solid-State NMR and Computational Modeling to Investigate the Structure and Dynamics of Membrane-Associated Ghrelin
Source: PLoS One. 2015 Mar 24;10(3):e0122444. doi: 10.1371/journal.pone.0122444 (PMC4372444; doi:10.1371/journal.pone.0122444)
Supplement: S2 File — (TGZ) [file pone.0122444.s008.tgz › ghrelin/folding_analysis/PSVS_analysis/fsvr/output_NAME.pdf]

Structure Quality Analysis for NAME

Analyses performed for all residues.
Procheck analysis,RMSD calculation and structure superimposition are based on: all residues

NESG ID:	NAME
PDB ID:	
Deposition date:	
Common Name:	
Class:	
Length (a.a.):	28
Organism:	
SwissProt / TrEMBL ID:	
# models:	22
Oligomerization:	monomer
Molecular weight:	3245

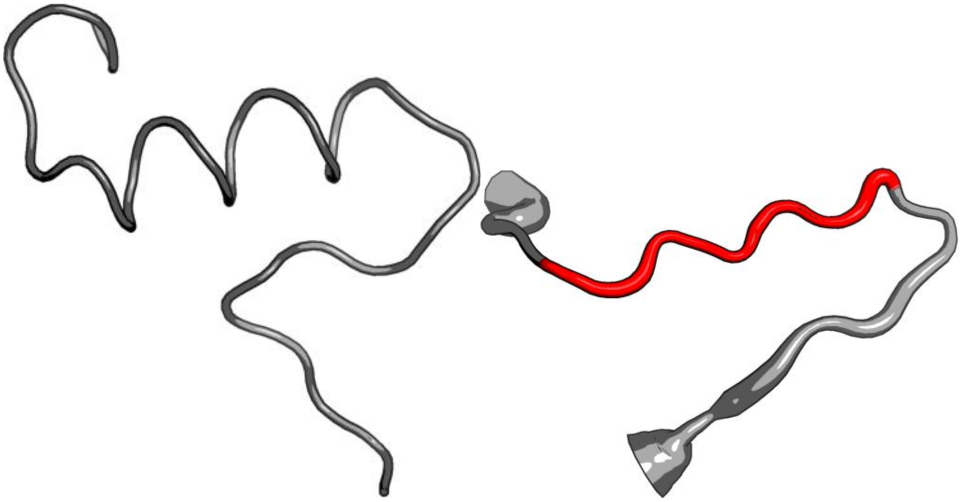

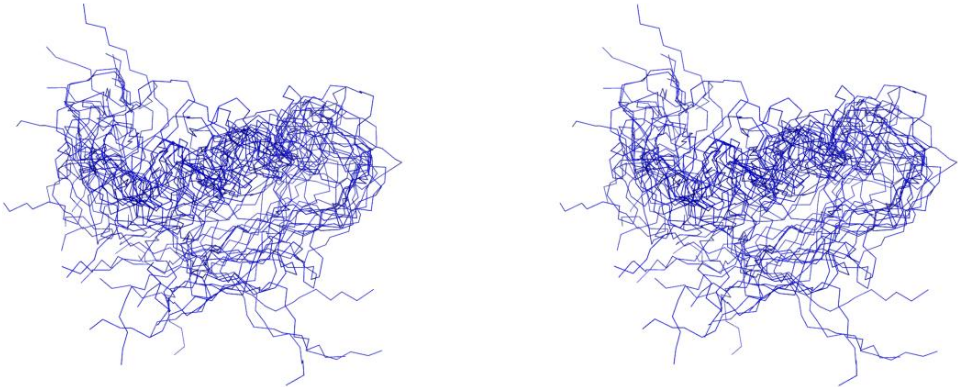

Secondary Structure Elements:
alpha helices: 7A-17A
beta strands:

FIDs deposited in the BMRB? no

RMSD	*All residues*	*Ordered residues[2]*	*Selected residues[3]*
All backbone atoms	4.0 Å	1.3 Å	4.0 Å
All heavy atoms	5.3 Å	2.5 Å	5.3 Å

Ramachandran Plot Summary for selected residues[3] from Procheck

Most favoured regions	*Additionally allowed regions*	*Generously allowed regions*	*Disallowed regions*
95.2%	4.8%	0.0%	0.0%

Ramachandran Plot Summary for selected residues[3] from Richardson Lab's Molprobity

Most favoured regions	*Allowed regions*	*Disallowed regions*	View plot	View model summary
99.5%	0.5%	0%		

Global quality scores

Program	*Verify3D*	*ProsaII (-ve)*	*Procheck (phi-psi)*[3]	*Procheck (all)*[3]	*MolProbity Clashscore*
-Raw score	0.24	0.60	0.13	0.29	3.01
Z-score[1]	-3.53	-0.21	0.83	1.71	1.01

Close Contacts and Deviations from Ideal Geometry (from PDB validation software)

Number of close contacts (within 1.6 &Aring for H atoms, 2.2 &Aring for heavy atoms):	0
RMS deviation for bond angles:	0.7 °
RMS deviation for bond lengths:	0.017 Å

[1] With respect to mean and standard deviation for a set of 252 X-ray structures < 500 residues, of resolution <= 1.80 Å, R-factor <= 0.25 and R-free <= 0.28; a positive value indicates a 'better' score

[2]Order residues: HASH(0xd7f3c0)

[3]Selected residues: all

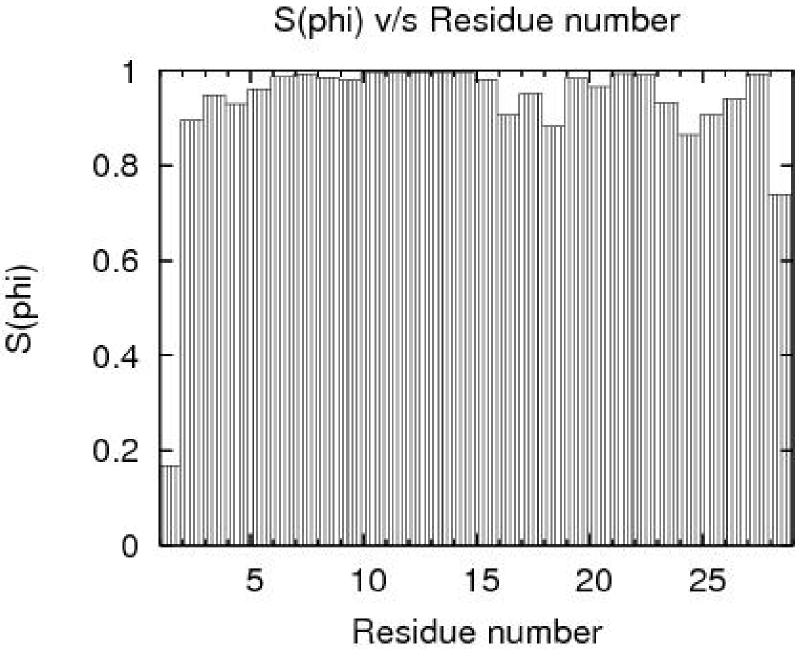

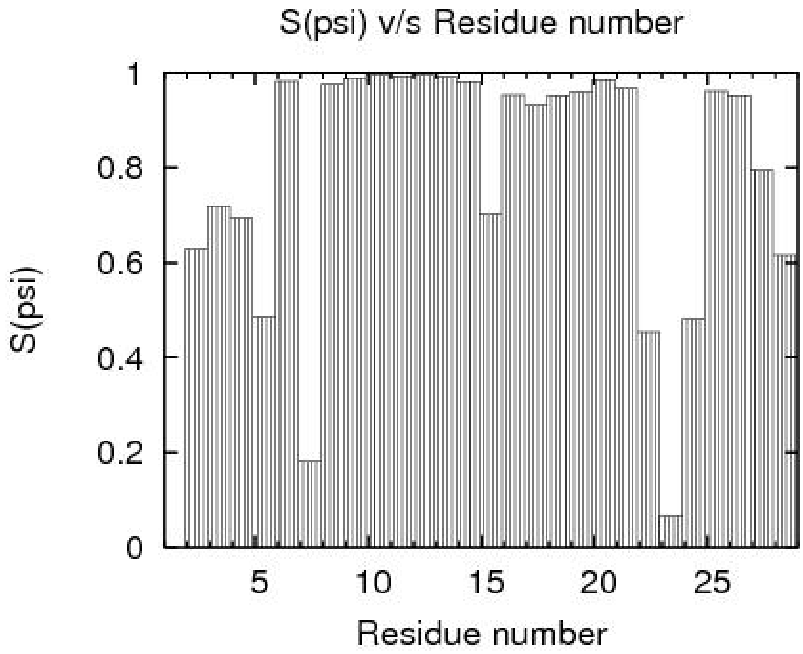
S(psi) v/s Residue number
S(psi)
1
0.8
0.6
0.4
0.2
0
5
10
15
20
25
Residue number

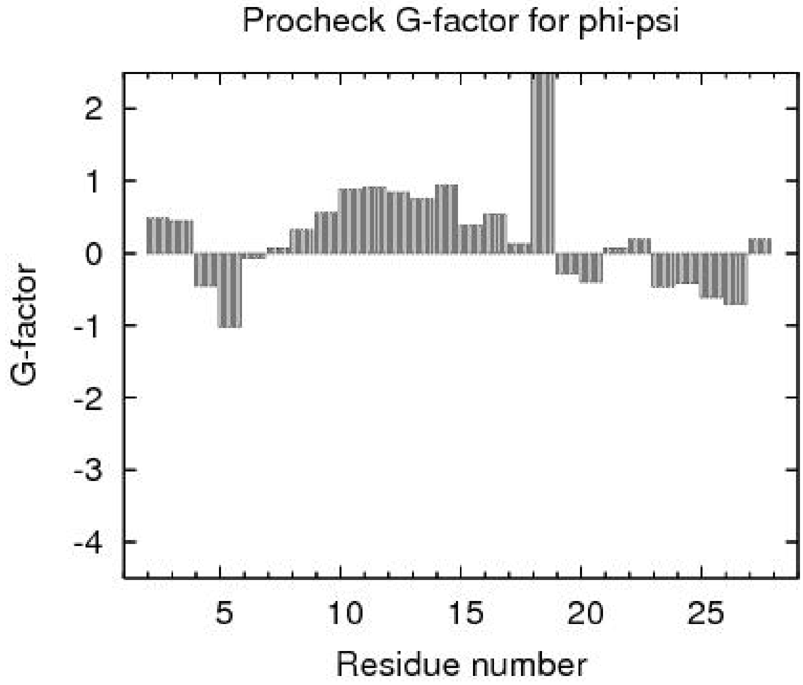
Procheck G-factor for phi-psi
G-factor
2
1
0
-1
-2
-3
-4
5
10
15
20
25
Residue number

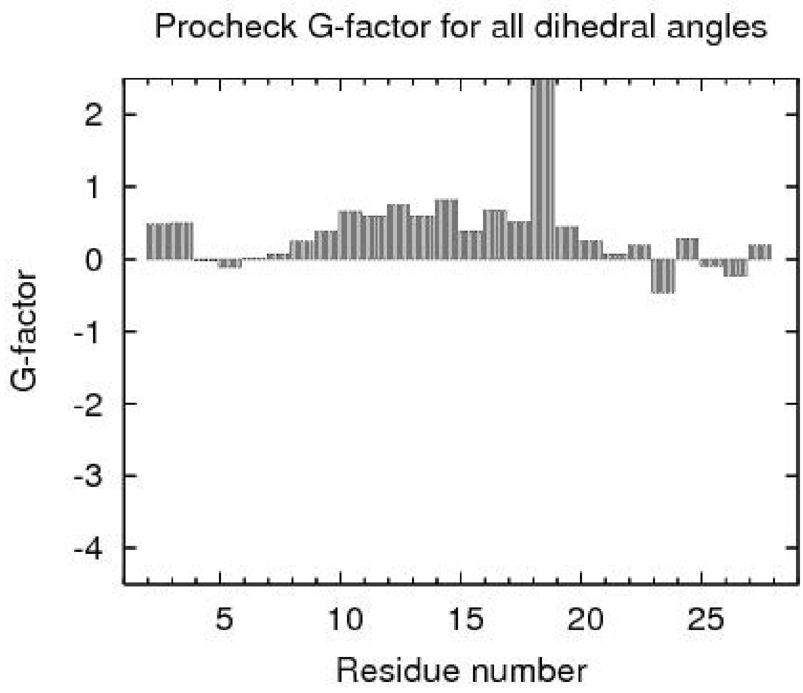
Procheck G-factor for all dihedral angles
G-factor
Residue number
2
1
0
-1
-2
-3
-4
5
10
15
20
25

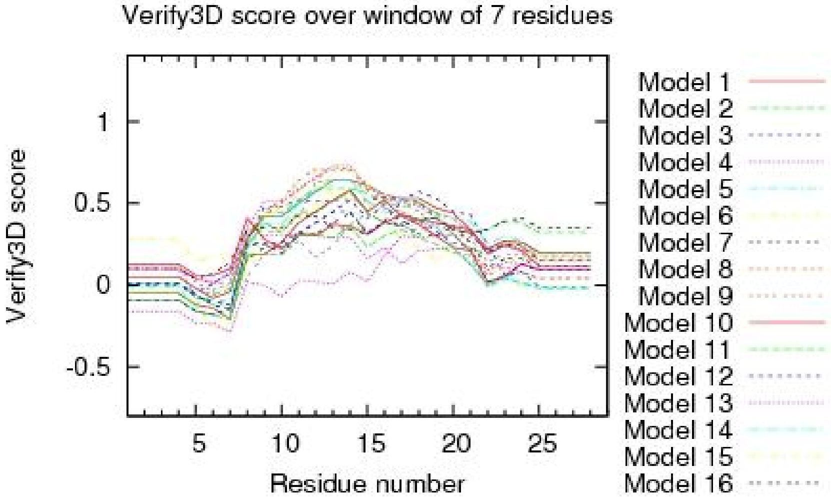
Verify3D score over window of 7 residues
Verify3D score
Residue number
1
0.5
0
-0.5
5
10
15
20
25
Model 1
Model 2
Model 3
Model 4
Model 5
Model 6
Model 7
Model 8
Model 9
Model 10
Model 11
Model 12
Model 13
Model 14
Model 15
Model 16

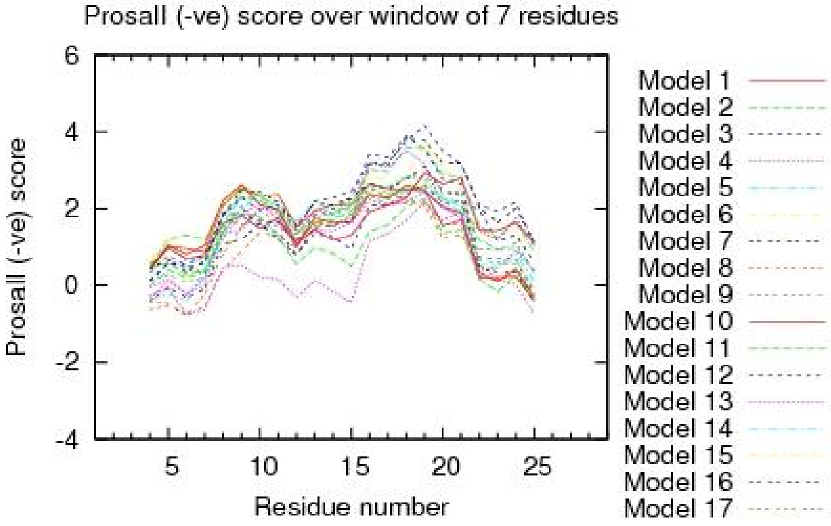

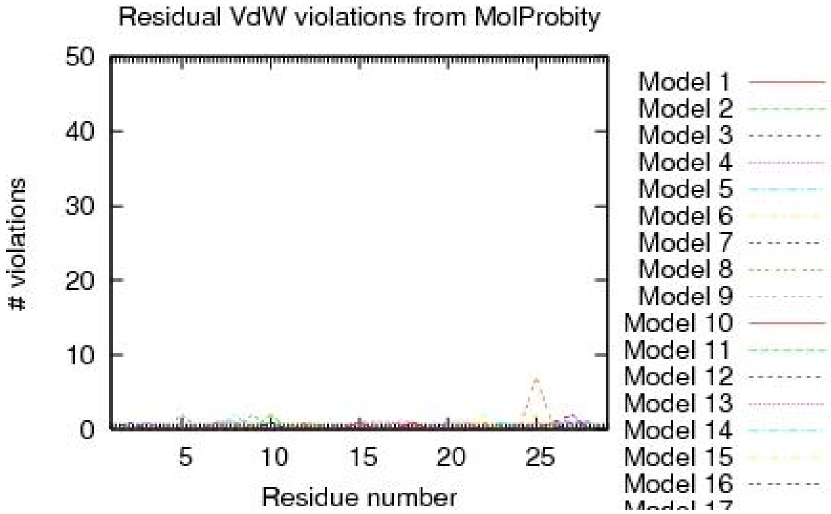

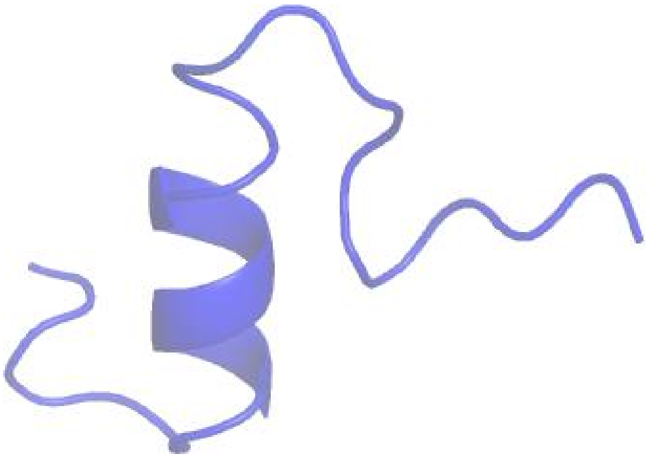

Residue Plot of Ramachandran anlysis(based on data from Richardson Lab's Molprobity)

References:

1. Luthy R, Bowie J U and Eisenberg D, "Assessment of protein models with three-dimensional profiles", Nature 356 (1992): 83-85
2. Bowie J U, Luthy R and Eisenberg D, "A Method to Identify Protein Sequences that Fold into a Known Three-Dimensional Structure", Science 253 (1991): 164-169
3. Sippl M J, "Recognition of Errors in Three-Dimensional Structures of Proteins", Proteins 17 (1993): 355-362
4. Sippl M J, "Calculation of Conformation Ensembles from Potentials of Mean Force", J Mol Biol 213 (1990): 859-883
5. Laskowski R Ai et al, "AQUA and PROCHECK_NMR: Programs for checking the quality of proteins structures solved by NMR", J Biomolec NMR 8 (1996): 477-486
6. Laskowski R A et al "PROCHECK: a program to check the stereochemical quality of protein structures" J Appl Cryst, 26 (1993): 283-291
7. Word J M et al, "Exploring steric constrains on protein mutations using MAGE / PROBE", Prot Sci 9 (2000): 2251-2259
8. Word J M et al, "Asparagine and Glutamine: Using Hydrogen Atom Contacts in the Choice of Side-chain Amide Orientation", J Mol Biol 285 (1999): 1735-1747
9. Word J M et al, "Visualizing and Quantifying Molecular Goodness-of-Fit: Small-probe Contact Dots with Explicit Hydrogens", J Mol Biol 285 (1999): 1711-1733
10. Tejero R and Montelione G T, "PDBStat", unpublished
11. Luthy R, McLachlan A D and Eisenberg D, "Secondary Structure-Based Profiles: Use of Structure-Conserving Scoring Tables in Searching Protein Sequence Databases for Structural Similarities", Proteins 10 (1991): 229-239
12. Richardson D C, Richardson J S, "The kinemage: a tool for scientific communication", Prot Sci 1(1) (1992): 3-9
13. Koradi, R, et al, "MOLMOL: a program for display and analysis of macromolecular structures ", J Mol Graphics 14 (1996): 51-55.
14. Güntert, P, Mumenthaler, C & Wüthrich, K "Torsion angle dynamics for NMR structure calculation with the new program DYANA", J. Mol. Biol 273 (1997): 283-298
15. Lovell S C et al, "Structure validation by Calpha geometry: phi,psi and Cbeta deviation" Proteins (2003) 50: 437-450
16. Kabsch W, Sander C, "Dictionary of protein secondary structure: pattern recognition of hydrogen-bonded and geometrical features", Biopolymers (1983) 22: 2577-2637
17. Bagaria, A., Jaravine, V., Huang, Y.J., Montelione, G.T., and Guntert, P. "Protein structure validation by generalized linear model root-mean-square deviation prediction". Protein Sci 21(2012), 229-238.

Protein Structure Validation Suite (PSVS)

Software Environment

Software for structure quality evaluation:

```
DSSP            DsspCMBI-April-2000
pdbstat         PdbStat-5.9 Version
AutoAssign      Version 2.4.0 (uses only AVS scripts)
RPF analysis    ASDP-1.0
PDB validation  Version 8.061
Verify3D        Version 1.0 corrected by Aneerban
ProsaII         Prosa2003
PROCHECK        Version 3.5.4
MolMol          Version 2K.2
```

MolProbity programs:

```
cluster             1999
clashlistcluster    1999 (corrected by Aneerban)
mage                Version 6.35.040409
prekin              Version 6.35.040406
reduce              Version 2.14
probe               Version 2.6
```

Other Software:

```
PERL            Version 5.8.0
convert         ImageMagick 5.5.6
ps2pdf          Ghostscript 7.05
htmldoc         v1.9
gnuplot         Version 3.7 patchlevel 3
jpegtopnm       year 2000
pnmcrop         year 2000
pnmtojpeg       year 2000
```
