# Supplementary material for: Integrating Solid-State NMR and Computational Modeling to Investigate the Structure and Dynamics of Membrane-Associated Ghrelin
Source: PLoS One. 2015 Mar 24;10(3):e0122444. doi: 10.1371/journal.pone.0122444 (PMC4372444; doi:10.1371/journal.pone.0122444)
Supplement: S2 File — (TGZ) [file pone.0122444.s008.tgz › ghrelin/folding_analysis/PSVS_analysis/mage_lnx.html]

Protein Structure Quality Analysis Result


the pdf file for MAGE VdW violations

the postscript file for MAGE VdW violations

JPEG image for MAGE VdW violations

Table of MAGE VdW violations for ordered residues across all models

List of bad contacts calculated by MAGE for model 1

List of bad contacts calculated by MAGE for model 2

List of bad contacts calculated by MAGE for model 3

List of bad contacts calculated by MAGE for model 4

List of bad contacts calculated by MAGE for model 5

List of bad contacts calculated by MAGE for model 6

List of bad contacts calculated by MAGE for model 7

List of bad contacts calculated by MAGE for model 8

List of bad contacts calculated by MAGE for model 9

List of bad contacts calculated by MAGE for model 10

List of bad contacts calculated by MAGE for model 11

List of bad contacts calculated by MAGE for model 12

List of bad contacts calculated by MAGE for model 13

List of bad contacts calculated by MAGE for model 14

List of bad contacts calculated by MAGE for model 15

List of bad contacts calculated by MAGE for model 16

List of bad contacts calculated by MAGE for model 17

List of bad contacts calculated by MAGE for model 18

List of bad contacts calculated by MAGE for model 19

List of bad contacts calculated by MAGE for model 20

List of bad contacts calculated by MAGE for model 21

List of bad contacts calculated by MAGE for model 22
