# Supplementary material for: Integrating Solid-State NMR and Computational Modeling to Investigate the Structure and Dynamics of Membrane-Associated Ghrelin
Source: PLoS One. 2015 Mar 24;10(3):e0122444. doi: 10.1371/journal.pone.0122444 (PMC4372444; doi:10.1371/journal.pone.0122444)
Supplement: S2 File — (TGZ) [file pone.0122444.s008.tgz › ghrelin/folding_analysis/PSVS_analysis/rama_each_molpro_lnx.html]

Protein Structure Quality Analysis Result


the pdf file from Richardson lab's Ramachandran analysis

Summary from Richardson lab's Ramachandran analysis

JPEG for residue Ramachandran Plots from Molprobity - Ensemble summary

JPEG for residue Ramachandran Plots from Molprobity - Model 1

JPEG for residue Ramachandran Plots from Molprobity - Model 2

JPEG for residue Ramachandran Plots from Molprobity - Model 3

JPEG for residue Ramachandran Plots from Molprobity - Model 4

JPEG for residue Ramachandran Plots from Molprobity - Model 5

JPEG for residue Ramachandran Plots from Molprobity - Model 6

JPEG for residue Ramachandran Plots from Molprobity - Model 7

JPEG for residue Ramachandran Plots from Molprobity - Model 8

JPEG for residue Ramachandran Plots from Molprobity - Model 9

JPEG for residue Ramachandran Plots from Molprobity - Model 10

JPEG for residue Ramachandran Plots from Molprobity - Model 11

JPEG for residue Ramachandran Plots from Molprobity - Model 12

JPEG for residue Ramachandran Plots from Molprobity - Model 13

JPEG for residue Ramachandran Plots from Molprobity - Model 14

JPEG for residue Ramachandran Plots from Molprobity - Model 15

JPEG for residue Ramachandran Plots from Molprobity - Model 16

JPEG for residue Ramachandran Plots from Molprobity - Model 17

JPEG for residue Ramachandran Plots from Molprobity - Model 18

JPEG for residue Ramachandran Plots from Molprobity - Model 19

JPEG for residue Ramachandran Plots from Molprobity - Model 20

JPEG for residue Ramachandran Plots from Molprobity - Model 21

JPEG for residue Ramachandran Plots from Molprobity - Model 22
