# Supplementary material for: Integrating Solid-State NMR and Computational Modeling to Investigate the Structure and Dynamics of Membrane-Associated Ghrelin
Source: PLoS One. 2015 Mar 24;10(3):e0122444. doi: 10.1371/journal.pone.0122444 (PMC4372444; doi:10.1371/journal.pone.0122444)
Supplement: S1 Table — (DOC) [file pone.0122444.s009.doc]

Table S1: Ensemble average RMSDs (in ppm) resulting from filtering strategies^a^

|  | **Top 10% by Rosetta Energy** | **Top 25% by Rosetta Energy** | **Top 50% by Rosetta Energy** | **Top 75% by Rosetta Energy** | **All** |
| --- | --- | --- | --- | --- | --- |
| PROSHIFT^b^ | 0.385 (22) | 0.381 (18) | 0.379 (26) | 0.378 (26) | 0.377 (26) |
| SHIFTX^c^ | 0.716 (18) | 0.713 (28) | 0.707 (23) | 0.707 (17) | 0.709 (13) |
| SHIFTX2^d^ | 0.722 (29) | 0.719 (26) | 0.718 (26) | 0.702 (26) | 0.717 (11) |
| SPARTA+^e^ | 0.718 (29) | 0.717 (20) | 0.711 (30) | 0.711 (24) | 0.718 (12) |
| # models in pool | 355 | 856 | 1,790 | 2,683 | 3,692 |

^a^ Ensemble size in parentheses

^b^ References^1-4^

^c^ References^2,4-7^

^d^ References^2,4,7-9^

^e^ References^9-11^
