# Supplementary material for: Integrating Solid-State NMR and Computational Modeling to Investigate the Structure and Dynamics of Membrane-Associated Ghrelin
Source: PLoS One. 2015 Mar 24;10(3):e0122444. doi: 10.1371/journal.pone.0122444 (PMC4372444; doi:10.1371/journal.pone.0122444)
Supplement: S2 Table — (DOC) [file pone.0122444.s010.doc]

Table S2: Overview of ghrelin peptide constructs and labeling schemes*

| GHR1: H2N- **G**SS(**n-octanoyl**)F**L** **S**PEHQ RVQQR KESKK PPAKL QPR -OH | M = 3398 Da |
| --- | --- |
| GHR2: H2N- G**S**S(n-octanoyl)FL S**PE**HQ RVQQR KESKK PPAKL QPR -OH | M = 3384 Da |
| GHR3: H2N- GS**S**(n-octanoyl)FL SPEH**Q** RVQQR KESKK P**P**AKL QPR -OH | M = 3385 Da |
| GHR4: H2N- GSS(n-octanoyl)**F**L SPEHQ R**V**QQR KESKK PP**A**KL QPR -OH | M = 3388 Da |
| GHR5: H2N- GSS(n-octanoyl)FL SPEHQ RV**Q**QR KE**S**KK **P**PAKL QPR -OH | M = 3385 Da |
| GHR6: H2N- GSS(n-octanoyl)FL SPEHQ RVQ**Q**R KESKK PPAKL Q**P**R -OH | M = 3379 Da |
| ∑: H2N- **GSS**(**n-octanoyl**)**FL SPE**H**Q** R**VQQ**R KE**S**KK **PPA**KL Q**P**R -OH |  |

* Several ghrelin peptides were synthesized having 17 of the 28 amino acids that were ^13^C/^15^N labeled. The peptides were allowed to bind to LUVs having a diameter of 100 nm and a composition of 80% DMPC-*d*_67_ and 20% DMPS-*d*_54_. Experiments were performed with 35 wt% of 10 mM MES buffer containing 10 mM NaCl at pH 6.
