# Supplementary material for: Integrating Solid-State NMR and Computational Modeling to Investigate the Structure and Dynamics of Membrane-Associated Ghrelin
Source: PLoS One. 2015 Mar 24;10(3):e0122444. doi: 10.1371/journal.pone.0122444 (PMC4372444; doi:10.1371/journal.pone.0122444)
Supplement: S3 Table — (DOC) [file pone.0122444.s011.doc]

Table S3: Detailed analysis of low-RMSD model from set of filtered models in top 10% by score

| **Res** | **Atom** | **CS_exp_^a,b^** | **CS_PROSHIFT_** | **| CS_PROSHIFT_ – CS_exp_ |c** | **CS_SPARTA+_** | **| CS_SPARTA+_ – CS_exp_ |** | **CS_SHIFTX_** | **| CS_SHIFTX_ – CS_exp_ |** | **CS_SHIFTX2_** | **| CS_SHIFTX2_– CS_exp_ |** |
| --- | --- | --- | --- | --- | --- | --- | --- | --- | --- | --- |
| Ser^2^ | C_O_ | 172.1 ± 0.2 | 173.0 | 0.2 | 176.5 | 1.1 | 174.9 | 0.7 | 176.28 | 1.0 |
| Ser^2^ | C_α_ | 55.6 ± 0.5 | 57.0 | 0.4 | 61.0 | 1.4 | 58.83 | 0.8 | 61.08 | 1.4 |
| Ser^2^ | C_β_ | 62.5 ± 0.6 | 62.3 | 0.0 | 63.0 | 0.1 | 62.98 | 0.1 | 63.06 | 0.1 |
| Ser^2^ | C_α_ | 53.6 ± 0.1 | 58.4 | 1.2 | 60.2 | 1.7 | 60.67 | 1.8 | 60.48 | 1.7 |
| Ser^2^ | C_β_ | 63.3 ± 0.2 | 61.4 | 0.5 | 62.6 | 0.2 | 63.78 | 0.1 | 62.65 | 0.2 |
| Ser^2^ | H_α_ | 4.5 ± 0.3 | 4.2 | 0.3 | 4.4 | 0.1 | 4.27 | 0.2 | 4.33 | 0.2 |
| Phe^4^ | C_O_ | 172.1 ± 0.2 | 174.5 | 0.6 | 176.2 | 1.0 | 175.45 | 0.8 | 176.27 | 1.0 |
| Phe^4^ | C_α_ | 55.8 ± 1.2 | 56.0 | 0.1 | 58.2 | 0.6 | 59.48 | 0.9 | 58.9 | 0.8 |
| Phe^4^ | C_β_ | 37.0 ± 0.8 | 38.0 | 0.3 | 39.0 | 0.5 | 38.85 | 0.5 | 39.36 | 0.6 |
| Leu^5^ | C_O_ | 174.8 ± 0.4 | 173.9 | 0.2 | 176.3 | 0.4 | 175.41 | 0.2 | 176.17 | 0.3 |
| Leu^5^ | C_α_ | 51.9 ± 0.2 | 51.5 | 0.1 | 53.6 | 0.4 | 53.3 | 0.4 | 54.2 | 0.6 |
| Leu^5^ | C_β_ | 40.7 ± 0.5 | 39.8 | 0.2 | 43.3 | 0.7 | 43.13 | 0.6 | 43.52 | 0.7 |
| Ser^6^ | C_O_ | 169.3 ± 0.5 | 172.2 | 0.7 | 172.7 | 0.8 | 172.64 | 0.8 | 173.32 | 1.0 |
| Ser^6^ | C_α_ | 54.2 ± 0.5 | 54.0 | 0.1 | 54.9 | 0.2 | 56.51 | 0.6 | 55.58 | 0.3 |
| Ser^6^ | C_β_ | 61.2 ± 0.6 | 62.8 | 0.4 | 64.1 | 0.7 | 63.58 | 0.6 | 63.85 | 0.7 |
| Pro^7^ | C_O_ | 174.9 ± 1.2 | 174.3 | 0.2 | 178.0 | 0.8 | 177.03 | 0.5 | 177.79 | 0.7 |
| Pro^7^ | C_α_ | 61.2 ± 0.6 | 61.3 | 0.0 | 62.3 | 0.3 | 62.01 | 0.2 | 62.11 | 0.2 |
| Pro^7^ | C_β_ | 30.8 ± 1.7 | 30.9 | 0.0 | 32.9 | 0.5 | 33.75 | 0.7 | 33.42 | 0.7 |
| Glu^8^ | C_O_ | 174.1 ± 0.2 | 176.0 | 0.5 | 178.9 | 1.2 | 178.78 | 1.2 | 178.75 | 1.2 |
| Glu^8^ | C_α_ | 54.3 ± 0.9 | 56.9 | 0.7 | 60.2 | 1.5 | 59.66 | 1.3 | 59.46 | 1.3 |
| Glu^8^ | C_β_ | 25.8 ± 0.9 | 27.4 | 0.4 | 29.0 | 0.8 | 29.09 | 0.8 | 29.23 | 0.9 |
| Gln^10^ | C_O_ | 177.3 ± 0.3 | 176.2 | 0.3 | 178.6 | 0.3 | 178.93 | 0.4 | 178.73 | 0.4 |
| Gln^10^ | C_α_ | 55.4 ± 0.4 | 57.4 | 0.5 | 59.3 | 1.0 | 58.88 | 0.9 | 59.11 | 0.9 |
| Gln^10^ | C_β_ | 27.0 ± 0.0 | 27.0 | 0.0 | 28.5 | 0.4 | 28.72 | 0.4 | 28.58 | 0.4 |
| Gln^10^ | H_α_ | 4.1 ± 0.3 | 3.9 | 0.2 | 3.8 | 0.3 | 3.93 | 0.2 | 3.98 | 0.2 |
| Val^12^ | C_O_ | 174.1 ± 0.2 | 175.5 | 0.3 | 177.0 | 0.7 | 177.75 | 0.9 | 178.15 | 1.0 |
| Val^12^ | C_α_ | 60.3 ± 0.9 | 63.9 | 0.9 | 65.7 | 1.3 | 66.1 | 1.5 | 65.84 | 1.4 |
| Val^12^ | C_β_ | 30.0 ± 0.3 | 28.8 | 0.3 | 31.5 | 0.4 | 31.63 | 0.4 | 31.75 | 0.4 |
| Gln^13^ | C_O_ | 173.5 ± 0.3 | 175.2 | 0.4 | 178.7 | 1.3 | 177.83 | 1.1 | 176.87 | 0.8 |
| Gln^13^ | C_α_ | 53.4 ± 0.2 | 56.3 | 0.7 | 57.1 | 0.9 | 58.16 | 1.2 | 57.74 | 1.1 |
| Gln^13^ | C_β_ | 27.0 ± 0.1 | 27.0 | 0.0 | 28.7 | 0.4 | 28.64 | 0.4 | 28.66 | 0.4 |
| Gln^13^ | H_α_ | 4.3 ± 0.3 | 4.1 | 0.2 | 4.1 | 0.2 | 4.11 | 0.2 | 4.14 | 0.2 |
| Gln^14^ | C_O_ | 173.5 | 173.9 | 0.1 | 176.3 | 0.7 | 176.43 | 0.7 | 176.36 | 0.7 |
| Gln^14^ | C_α_ | 53.4 ± 0.1 | 55.0 | 0.4 | 56.6 | 0.8 | 57.55 | 1.0 | 57.21 | 0.9 |
| Gln^14^ | C_β_ | 27.0 ± 0.1 | 26.5 | 0.1 | 29.0 | 0.5 | 29.58 | 0.7 | 29.14 | 0.5 |
| Gln^14^ | H_α_ | 4.3 | 4.3 | 0.0 | 4.1 | 0.2 | 4.07 | 0.2 | 4.19 | 0.1 |
| Ser^18^ | C_O_ | 171.8 ± 0.2 | 173.2 | 0.4 | 174.8 | 0.8 | 174.27 | 0.6 | 173.9 | 0.5 |
| Ser^18^ | C_α_ | 55.8 ± 0.1 | 56.4 | 0.2 | 57.9 | 0.5 | 57.89 | 0.5 | 58.23 | 0.6 |
| Ser^18^ | C_β_ | 61.3 ± 0.2 | 60.3 | 0.3 | 64.7 | 0.8 | 64.13 | 0.7 | 64.47 | 0.8 |
| Ser^18^ | H_α_ | 4.4 ± 0.3 | 4.4 | 0.0 | 4.5 | 0.0 | 4.42 | 0.0 | 4.41 | 0.0 |
| Pro^21^ | C_O_ | 177.7 ± 0.3 | 173.4 | 1.1 | 175.1 | 0.6 | 174.84 | 0.7 | 175.33 | 0.6 |
| Pro^21^ | C_α_ | 59.0 ± 0.1 | 59.1 | 0.0 | 61.8 | 0.7 | 61.79 | 0.7 | 62.32 | 0.8 |
| Pro^21^ | C_β_ | 28.3 ± 0.0 | 29.8 | 0.4 | 31.5 | 0.8 | 31.89 | 0.9 | 30.97 | 0.7 |
| Pro^21^ | H_α_ | 4.7 ± 0.3 | 4.6 | 0.1 | 4.4 | 0.3 | 4.42 | 0.3 | 4.6 | 0.1 |
| Pro^22^ | C_O_ | 173.7 ± 0.2 | 174.3 | 0.2 | 176.1 | 0.6 | 176.41 | 0.7 | 177.46 | 0.9 |
| Pro^22^ | C_α_ | 60.4 ± 0.2 | 60.7 | 0.1 | 62.6 | 0.5 | 62.68 | 0.6 | 62.72 | 0.6 |
| Pro^22^ | C_β_ | 29.4 ± 0.0 | 30.9 | 0.4 | 32.3 | 0.7 | 31.89 | 0.6 | 32.19 | 0.7 |
| Pro^22^ | H_α_ | 4.4 ± 0.3 | 4.5 | 0.1 | 4.1 | 0.4 | 4.21 | 0.2 | 4.42 | 0.0 |
| Ala^23^ | C_O_ | 175.5 ± 0.5 | 175.0 | 0.1 | 177.1 | 0.4 | 177.18 | 0.4 | 177.2 | 0.4 |
| Ala^23^ | C_α_ | 50.5 ± 0.6 | 50.2 | 0.1 | 51.4 | 0.2 | 51.51 | 0.3 | 51.62 | 0.3 |
| Ala^23^ | C_β_ | 17.0 ± 0.3 | 16.8 | 0.0 | 20.3 | 0.8 | 20.15 | 0.8 | 19.27 | 0.6 |
| Pro^27^ | C_O_ | 173.3 ± 0.0 | 175.0 | 0.4 | 176.0 | 0.7 | 176.67 | 0.8 | 177.01 | 0.9 |
| Pro^27^ | C_α_ | 60.8 ± 0.0 | 61.7 | 0.2 | 63.1 | 0.6 | 62.73 | 0.5 | 63.13 | 0.6 |
| Pro^27^ | C_β_ | 29.4 ± 0.1 | 30.2 | 0.2 | 32.4 | 0.7 | 32.32 | 0.7 | 31.98 | 0.6 |
| Pro^27^ | H_α_ | 4.4 ± 0.3 | 4.4 | 0.1 | 4.6 | 0.1 | 4.37 | 0.1 | 4.4 | 0.0 |
| **Average Deviation (± S.E.M.)** | | | **0.3 ± 0.04** | | **0.6 ± 0.05** | | **0.6 ± 0.05** | | **0.6 ± 0.05** | |
| **RMSD** | | | **0.4** | | **0.7** | | **0.7** | | **0.7** | |

^a^ All values in ppm

^b^ Experimental and predicted values not scaled. Difference values take scaling into account.

^c^ All CS differences (in | |) are scaled. Scaling = CS_carbon_ * 0.25
